# Intermittent Preventive Treatment of Malaria in Pregnancy with Mefloquine in HIV-Negative Women: A Multicentre Randomized Controlled Trial

**DOI:** 10.1371/journal.pmed.1001733

**Published:** 2014-09-23

**Authors:** Raquel González, Ghyslain Mombo-Ngoma, Smaïla Ouédraogo, Mwaka A. Kakolwa, Salim Abdulla, Manfred Accrombessi, John J. Aponte, Daisy Akerey-Diop, Arti Basra, Valérie Briand, Meskure Capan, Michel Cot, Abdunoor M. Kabanywanyi, Christian Kleine, Peter G. Kremsner, Eusebio Macete, Jean-Rodolphe Mackanga, Achille Massougbodgi, Alfredo Mayor, Arsenio Nhacolo, Golbahar Pahlavan, Michael Ramharter, María Rupérez, Esperança Sevene, Anifa Vala, Rella Zoleko-Manego, Clara Menéndez

**Affiliations:** 1 Barcelona Centre for International Health Research (CRESIB, Hospital Clínic-Universitat de Barcelona), ISGlobal, Barcelona Institute for Global Health, Barcelona, Spain; 2 Manhiça Health Research Center (CISM), Manhiça, Mozambique; 3 Centre de Recherches Médicales de Lambaréné (CERMEL), Albert Schweitzer Hospital, Lambaréné, Gabon; 4 Institute of Tropical Medicine, University of Tübingen, Tübingen, Germany; 5 Faculté des Sciences de la Santé (FSS), Université d'Aboméy Calavi, Cotonou, Benin; 6 Institut de Recherche pour le Développement (IRD), Paris, France; 7 Ifakara Health Institute (IHI), Dodoma, Tanzania; 8 Université René Descartes, Paris, France; 9 Department of Medicine I, Division of Infectious Diseases and Tropical Medicine, Medical University of Vienna, Vienna, Austria; 10 Ngounie Medical Research Centre, Fougamou, Gabon; National Institute of Child Health and Human Development, United States of America

## Abstract

**Background:**

Intermittent preventive treatment in pregnancy (IPTp) with sulfadoxine-pyrimethamine (SP) is recommended by WHO to prevent malaria in African pregnant women. The spread of SP parasite resistance has raised concerns regarding long-term use for IPT. Mefloquine (MQ) is the most promising of available alternatives to SP based on safety profile, long half-life, and high efficacy in Africa. We evaluated the safety and efficacy of MQ for IPTp compared to those of SP in HIV-negative women.

**Methods and Findings:**

A total of 4,749 pregnant women were enrolled in an open-label randomized clinical trial conducted in Benin, Gabon, Mozambique, and Tanzania comparing two-dose MQ or SP for IPTp and MQ tolerability of two different regimens. The study arms were: (1) SP, (2) single dose MQ (15 mg/kg), and (3) split-dose MQ in the context of long lasting insecticide treated nets. There was no difference on low birth weight prevalence (primary study outcome) between groups (360/2,778 [13.0%]) for MQ group and 177/1,398 (12.7%) for SP group; risk ratio [RR], 1.02 (95% CI 0.86–1.22; *p* = 0.80 in the ITT analysis). Women receiving MQ had reduced risks of parasitemia (63/1,372 [4.6%] in the SP group and 88/2,737 [3.2%] in the MQ group; RR, 0.70 [95% CI 0.51–0.96]; *p* = 0.03) and anemia at delivery (609/1,380 [44.1%] in the SP group and 1,110/2743 [40.5%] in the MQ group; RR, 0.92 [95% CI 0.85–0.99]; *p* = 0.03), and reduced incidence of clinical malaria (96/551.8 malaria episodes person/year [PYAR] in the SP group and 130/1,103.2 episodes PYAR in the MQ group; RR, 0.67 [95% CI 0.52–0.88]; *p* = 0.004) and all-cause outpatient attendances during pregnancy (850/557.8 outpatients visits PYAR in the SP group and 1,480/1,110.1 visits PYAR in the MQ group; RR, 0.86 [0.78–0.95]; *p* = 0.003). There were no differences in the prevalence of placental infection and adverse pregnancy outcomes between groups. Tolerability was poorer in the two MQ groups compared to SP. The most frequently reported related adverse events were dizziness (ranging from 33.9% to 35.5% after dose 1; and 16.0% to 20.8% after dose 2) and vomiting (30.2% to 31.7%, after dose 1 and 15.3% to 17.4% after dose 2) with similar proportions in the full and split MQ arms. The open-label design is a limitation of the study that affects mainly the safety assessment.

**Conclusions:**

Women taking MQ IPTp (15 mg/kg) in the context of long lasting insecticide treated nets had similar prevalence rates of low birth weight as those taking SP IPTp. MQ recipients had less clinical malaria than SP recipients, and the pregnancy outcomes and safety profile were similar. MQ had poorer tolerability even when splitting the dose over two days. These results do not support a change in the current IPTp policy.

**Trial registration:**

ClinicalTrials.gov NCT 00811421; Pan African Clinical Trials Registry PACTR 2010020001429343

*Please see later in the article for the Editors' Summary*

## Abstract 2

Clara Menéndez and colleagues conducted an open-label randomized controlled trial in HIV-negative pregnant women in Benin, Gabon, Mozambique, and Tanzania to evaluate the safety and efficacy of mefloquine compared to sulfadoxine-pyrimethamine for intermittent preventative therapy for malaria.

*Please see later in the article for the Editors' Summary*

## Editors' Summary

**Background:**

Half the world's population is at risk of malaria, a mosquito-borne parasitic disease that kills about 600,000 people every year. Most of these deaths occur among young children in sub-Saharan Africa but pregnant women and their unborn children living in Africa are also very vulnerable to malaria. Infection with malaria during pregnancy can cause severe maternal anemia (reduced red blood cell numbers), stillbirths, and pre-term and low-birthweight babies, and is responsible for the deaths of many African babies and women. To prevent this loss of life, the World Health Organization (WHO) recommends a three-pronged approach—the delivery to pregnant women of the antimalarial drug sulfadoxine-pyrimethamine (SP) at each scheduled antenatal care visit given at least one month apart (intermittent preventive treatment in pregnancy; IPTp), the use of insecticide treated bed nets to protect pregnant women from the bites of infected mosquitoes, and effective case management of pregnant women with malarial illness.

**Why Was This Study Done?:**

IPTp with SP reduces the delivery of low-birth-weight babies and neonatal deaths but malaria parasites are becoming resistant to SP. Thus, other antimalarial drugs need to be evaluated for use in IPTp. Suitable drugs need to remain in the body for a long time to maximize their prophylactic (preventative) effect, they need to be given as a single dose at antenatal clinic visits to ensure compliance, and they must not harm the unborn child. In this open-label, randomized controlled trial (RCT), the researchers compare the efficacy and safety of IPTp with SP and mefloquine (MQ, an antimalarial drug that matches these criteria) in HIV-negative women living in Africa. The study also compares the tolerability of two MQ regimens. RCTs compare outcomes in groups of people chosen to receive different interventions through the play of chance; in open-label RCTs, both the researchers and the study participants know which treatment is being administered. IPTp with SP is only recommended for HIV-negative women because SP interacts with cotrimoxazole, which is routinely given to HIV-positive individuals to prevent infections.

**What Did the Researchers Do and Find?:**

The researchers assigned 4,749 pregnant women in Benin, Gabon, Mozambique, and Tanzania to one of three study groups. Participants in the SP and MQ groups received two doses of SP or MQ, respectively, administered at least one month apart. Participants in the split-dose MQ group received each MQ dose as half doses given on consecutive days. The prevalence of low-birth-weight deliveries (the study's primary outcome; the prevalence of a condition is the proportion of a population with that condition) was similar in the SP group and in the combined MQ groups. However, compared to women who received SP, women who received MQ had a lower risk of parasitemia (parasites in the blood), a lower risk of anemia at delivery, fewer episodes of clinical malaria, and fewer outpatient attendances. The prevalence of placental infection with malaria parasites and of adverse pregnancy outcomes such as stillbirth was similar in all the study groups. Finally, the tolerability of IPTp was poorer in the two MQ intervention groups than in the SP group, but similar proportions of adverse events (mainly dizziness and vomiting) were reported for the two MQ dosing regimens.

**What Do These Findings Mean?:**

These findings indicate that HIV-negative African women taking MQ for IPTp had a similar risk of a low-birth-weight delivery (the study's primary outcome) and lower risk of malaria illness during pregnancy than women taking SP for IPTp. Because the study did not have a no-IPTp arm (for ethical reasons), these findings provide no information about the efficacy or safety or either MQ or SP *per se*; these findings only indicate that MQ is no more efficacious than SP in the prevention of low-birth-weight babies. Moreover, because the study was open-label, the accuracy of the findings related to the tolerability and safety of MQ compared to SP may be limited because of biases in the assessment of safety outcomes. Given that the MQ dose used here for IPTp was associated with poorer tolerability than that of SP, these findings do not support the use of MQ instead of SP for IPTp.

**Additional Information:**

Please access these websites via the online version of this summary at http://dx.doi.org/10.1371/journal.pmed.1001733.

- A related *PLOS Medicine* Research Article by Raquel González and colleagues examines IPTp-MQ in HIV-infected women receiving cotrimoxazole prophylaxis
- This study is further discussed in a *PLOS Medicine* Perspective by Richard Steketee.
- Information is available from the World Health Organization on malaria (in several languages) and on malaria in pregnancy; information on IPTp and the updated WHO policy recommendation on IPTp with SP are available; the 2013 World Malaria Report provides details of the current global malaria situation
- The US Centers for Disease Control and Prevention also provides information on malaria; a personal story about malaria in pregnancy is available
- Information is available from the Roll Back Malaria Partnership on all aspects of global malaria control, including information on malaria in pregnancy
- The Malaria in Pregnancy Consortium is undertaking research into the prevention and treatment of malaria in pregnancy
- MedlinePlus provides links to additional information on malaria (in English and Spanish)

## Introduction

As the scourge of malaria continues, special considerations regarding the management of the infection in the most vulnerable groups are needed to achieve maximum safety and efficacy of control strategies. Owing to not yet well established physiological reasons, pregnant women are more susceptible to the effects of malaria infection with increased associated morbidity and mortality both in the mother and her newborn [1]–[4]. Thus, pregnant women are a vulnerable group for malaria who require particular attention [5], which is especially relevant in the African region where nearly 30 million pregnancies occur every year in areas where there is stable transmission of *Plasmodium falciparum*, the most deleterious of the human malaria parasites [3]. Because of this concern, pregnant women in Africa are currently the only population group in whom malaria preventive measures are routinely implemented. These measures rely on the use of long-lasting insecticide treated nets (LLITNs) and the administration of intermittent preventive treatment in pregnancy with sulfadoxine-pyrimethamine (IPTp-SP) [6]. Until the last World Health Organization (WHO) revision of the guidelines, it was recommended that women should receive at least two doses of IPTp-SP starting from the second trimester and at least one month apart to prevent malaria during pregnancy [6],[7]. These guidelines apply only to HIV negative women, while for HIV positive women IPT-SP is contraindicated to avoid safety interactions with cotrimoxazole prophylaxis [7],[8].

IPTp-SP has been shown to reduce low birth weight (LBW) deliveries and maternal morbidity [1],[9]. Because it is delivered through an existing health infrastructure such as the antenatal care (ANC) clinic scheme and due to the low cost of SP, IPTp remains a cost-effective intervention even in areas of relatively low malaria transmission and reduced efficacy levels of the drug due to parasite resistance [10]. However, drug resistance can evolve rapidly and a reduction in the efficacy of SP would reduce its beneficial impact on clinical delivery outcomes and worsen the cost-effectiveness of the intervention [11]. It has been shown that within certain parameters improving the antimalarial's efficacy would ameliorate the cost-effectiveness of the intervention despite an increase in its cost [10]. Thus, the evaluation of alternative antimalarial drugs to SP for IPTp is needed for optimal health decision-making especially in resource-limited countries.

The decision process on the best candidate to replace SP for IPTp needs to consider that a potential alternative drug should have at least three main attributes, namely: have a long half-life to maximize the prophylactic effect, be administered in single dose to ensure compliance, and have an acceptable reproductive toxicity profile [12],[13]. Of all the available antimalarial drugs, mefloquine (MQ), from the arylaminoalcohols group, is currently the one that matches these criteria. Contrary to the situation in parts of Southeast Asia, MQ retains high antimalarial activity in Africa as evidenced by both in vitro and in vivo studies [14]–[17]. MQ is among the very few antimalarials considered safe throughout pregnancy to be recommended by the WHO and the US Centers for Disease Control (CDC) for chemoprophylaxis for pregnant women of all gestational ages travelling in malaria endemic regions. It has recently been reclassified as pregnancy category B by the US Food and Drug Administration (FDA) (“Animal reproduction studies have failed to demonstrate a risk to the foetus and there are no adequate and well-controlled studies in pregnant women”) [16],[18]. Regarding drug tolerability and safety, MQ has been associated with mild and transient vomiting and dizziness as well as rare neurological and psychological severe adverse effects [19]. Evidence from studies in patients with uncomplicated malaria from Southeast Asia showed that MQ tolerability is improved by splitting the total dose over two days of administration [20]. However, it is unknown whether these results could be extrapolated when MQ is administered to asymptomatic or un-infected pregnant women. One concern in relation to the use of MQ in pregnancy has been the potential association of the drug with an increased risk of stillbirths. This finding was reported in a retrospective analysis among 208 Karen women who received MQ for malaria treatment [21]. This finding was not confirmed in studies including a larger prospective clinical trial of MQ prophylaxis in Malawian pregnant women, but the impact of MQ on birth outcomes remains controversial [22].

The information to date regarding MQ as IPTp is limited to two trials carried out in Benin, which have provided encouraging results [23],[24]. In light of the lack of available alternatives to replace SP it is necessary to confirm that MQ constitutes an adequate alternative for IPTp in a large study. In order to evaluate the safety and efficacy of MQ as IPTp in comparison to SP, in HIV negative women, a randomized controlled trial was conducted in four sub-Saharan countries in the context of LLITN use. The study also assessed the tolerability of MQ when administered as a split dose over two days.

## Methods

### Ethics Statement and Participants' Safety

The study protocol and informed consent forms were reviewed and approved by the Ethics Committees from the Hospital Clínic of Barcelona (Spain), the Comité Consultatif de Déontologie et d'Éthique (CCDE) from the Institut de Recherche pour le Développement (IRD, in France), and all local regulatory authorities and National Ethics Review Committees from each malaria endemic country participating in the study (Table S1). The trial was conducted under the provisions of the Declaration of Helsinki and in accordance with Good Clinical Practices guidelines set up by the WHO and by the International Conference on Harmonization. An independent Data Safety Monitoring Board (DSMB) was created prior to the beginning of the trial and regularly reviewed and monitored the safety data collected. The trial was registered prior to the enrolment of the first participant in both the ClinicalTrials.gov (NCT0081121) and in the Pan African Clinical Trials (PACTR2010020001429343) registries.

### Study Area and Population

The study was conducted between 2009 and 2013 in four sub-Saharan countries: Benin (Allada, Sékou, and Attogon), Gabon (Lambaréné and Fougamou), Tanzania (Makole and Chamwino), and Mozambique (Manhiça and Maragra). The characteristics of each site are shown in Table S2.

### Study Design

The study was designed as an open-label, randomized, three-arm trial to compare two-dose MQ with two-dose SP for IPTp, and to compare the tolerability of two different MQ administration regimens in the context of LLITN use. The three study arms were: (1) IPTp with SP, (2) IPTp with MQ (15 mg/kg) given once as a full dose, and (3) IPTp with MQ (15 mg/kg) split over two days. The primary endpoint of the study was the prevalence of LBW babies (<2,500 g). On the basis of previous estimations in the study sites [9],[25], of a LBW prevalence of 12% in the context of IPTp-SP and LLITN use and an estimated 25% reduction to 9% in the MQ group, 1,257 women in the SP arm and 2,514 women equally split between the two MQ arms were needed to show superiority of MQ compared to SP in reducing LBW rates, at the 5% two-sided level of significance with 80% statistical power (Texts S1 and S2).

### Enrolment and Interventions

Pregnant women of all gravidities attending an ANC clinic for the first time and who had not received IPTp during their current pregnancy were invited to participate in the study after provision of informed consent. Inclusion criteria were: permanent residence in the study area, gestational age ≤28 weeks, negative HIV-testing at recruitment, absence of history of allergy to sulfa drugs or MQ, absence of history of severe renal, hepatic, psychiatric, or neurological disease, and of MQ or halofantrine treatment in the preceding 4 weeks. Gestational age was determined from fundal height measurement by bimanual palpation. Women not meeting inclusion criteria received standard ANC following national guidelines. Hemoglobin (Hb), HIV test and the syphilis rapid plasma reagin test (RPR) were assessed at the first antenatal visit as per local standard procedures. In Mozambique and Tanzania, HIV-infected women were invited to participate in a placebo-controlled trial evaluating MQ IPTp in women on daily cotrimoxazole prophylaxis [26]. The allocation of the participants to the study arms was done centrally by randomization stratified by country according to a 1∶1∶1 scheme. The sponsor's institution biostatistician produced the computer-generated randomization list for each recruiting site. Treatment allocation for each participant was concealed in opaque sealed envelopes that were opened only after recruitment. Study participants were assigned a unique study number linked to the allocated treatment group. All participants received a LLITN (PermaNet, Vestergaard Fransen) at enrolment as part of the study intervention.

Following physical examination, recruited women with gestational age ≥13 weeks received their first dose of IPTp (either SP or MQ) under supervision. Women allocated to the SP group received standard IPTp (three tablets of the fixed combination therapy containing 500 mg of sulfadoxine and 25 mg of pyrimethamine, Malastop, Sterop), whereas participants allocated to the MQ groups received 15 mg/kg of the drug (Lariam, Roche, tablets of 250 mg of MQ base). The number of tablets was calculated according to body weight, thus a woman weighing 70 kg would receive four and a quarter tablets. The maximum dosage would not exceed 1,500 mg of MQ base corresponding to six tablets. For women allocated to the MQ split dose group, the 15 mg/kg dose was divided into two halves and administered over two consecutive days with the second half dose administered either at the ANC clinic or at home (by study personnel). All study participants were observed for 60 minutes following IPT administration. Women who vomited within the first 30 minutes were provided a second full IPT dose and those vomiting 30–60 minutes after drug intake were given a half replacement dose. Home visits by field workers were done two days after IPTp administration to assess drug tolerability and correct LLITN use. The second IPTp-SP/MQ administration was given at least one month later than the first one.

### Follow-up

Women were encouraged to attend the ANC clinic whenever they had any health complaint. Health care was free of charge and in general there was little availability of antimalarial drugs over the counter at all sites. A health facility-based passive surveillance system was established at each site to capture unscheduled visits of the study participants during the study follow-up. At each unscheduled visit, a standardized questionnaire was completed documenting signs and symptoms. Blood smears were prepared for malaria parasite examination and hemoglobin was measured if there were current or reported symptoms and/or signs suggestive of malaria. Clinical malaria episodes were treated with oral quinine or artemether-lumefantrine in the first and subsequent trimesters, respectively, for uncomplicated malaria, and with parenteral quinine for severe malaria. Solicited and unsolicited adverse events (AEs) were assessed. The former was done by directed questioning of malaria related signs and symptoms during unscheduled visits, whereas the latter were assessed through open questioning during scheduled visits. Women who were withdrawn from the study received routine ANC treatment.

At delivery, women's peripheral blood, cord blood, and placental (biopsy and impression smears) samples were collected for hematological and parasitological evaluation. Newborns were weighed (weekly calibrated scales, either digital or three beam balances), and their gestational age at birth evaluated using the Ballard's score [27]. Newborn weights not captured at birth but within the first week of life were estimated using a linear regression model (Figure S1) [28]. One month after the end of pregnancy, a capillary blood sample from the mother was collected for malaria parasite determination. LLITN use was assessed at each study visit by questions about use the preceding night.

### Laboratory Methods

At enrolment, HIV and syphilis serostatus were assessed at each site according to local standard procedures using rapid diagnostic tests (Table S2). Hemoglobin was determined using mobile devices in capillary blood sample (HemoCue [www.eurotrol.com] and Hemocontrol [www.ekfdiagnostics.com]). Thick and thin blood films were stained and read for *Plasmodium* species detection according to standard, quality-controlled procedures [29],[30]. Tissue samples were collected from the maternal side of the placenta and placed into 10% neutral buffered formalin. Biopsies were processed, stained, and examined following standard procedures [31]. Impression smears from the placental blood were stained with Giemsa and read following a standardized protocol [32],[33].

### Data Management, Statistical Methods, and Definitions

The quality of the data recorded in the study source documents and case report forms (CRFs) were monitored regularly following Good Clinical Practices principles by the trials' clinical monitor before their shipment to the centralized database in Manhiça, Mozambique. Data were double-entered using the OpenClinica Enterprise software for clinical data management (www.openclinica.com). The analysis was done on the modified Intention to Treat (ITT) cohort that included all recruited women who met the inclusion criteria and had data on the specific outcomes, and the According to Protocol (ATP) cohort that included all women who had received the two doses of IPTp according to the pre-specified schedule, had delivered singletons, and whose babies' weight (including stillbirths) had been recorded. The safety cohort was defined as all recruited women who had received at least one dose of IPTp and whose data for analysis were available. The analysis of safety and tolerability was made on the safety cohort. The ITT analyses were adjusted by country. The analyses in the ATP cohort were adjusted by baseline covariates (seasonality, gestational age, gravidity, anemia, literacy, and middle upper arm circumference). To include seasonality in the adjusted analysis, the duration of recruitment was divided into eight periods, and the interaction terms between the periods of recruitment and country were included in the model, which allows modelling the effect of period in each country independently. Proportion of low birth weight babies (<2,500 g at birth) were compared between groups using a modified binomial regression [34]. Only birth weights captured during the first week of life were included in the analysis. The interpretation of the statistical analysis for efficacy followed a sequential approach [35]. First, based on the confidence intervals, non-inferiority between the proportion of LBW in the MQ (combined MQ full and split dose groups) and the SP groups was evaluated assuming a 25% reduction in LBW prevalence as non-inferiority margin. If non-inferiority between MQ and SP was achieved, then a superiority interpretation comparing the groups was planned. The statistical analysis plan is available (Text S3).

Malaria infection was defined as the presence of asexual *P. falciparum* parasites of any density in a blood smear. A clinical malaria episode was defined as the latter plus any sign and/or symptom suggestive of malaria including: fever (axillary temperature ≥37.5°C) in the last 24 hours, and/or pallor and/or arthromyalgias and/or headache and/or history of convulsions [36]. The incidence of all clinical malaria episodes was compared between groups using a negative binomial regression allowing for interdependence between episodes within the same subject, excluding from the time at risk the 28 days after a malaria episode. Failure curves were produced using the Kaplan-Meier methodology. Placental infection was defined as the presence of parasites with or without pigment in the histological examination, or in the impression smear [31]–[33]. Anemia was defined as a Hb level <11 g/dl and severe anemia as Hb<7 g/dl. Immediate tolerability to study drugs was assessed as observed vomiting within one hour of drug administration. An AE was defined as any untoward medical occurrence in a study participant, to whom the study drug was administered, including occurrences, which are not necessarily caused or related to that drug. Serious adverse events (SAEs) were defined as an AE that met any of the following criteria: (1) results in death, (2) is life-threatening, (3) requires hospitalization (or prolongation of existing hospitalization), (4) results in disability/incapacity, (5) is a congenital anomaly, or (6) any event of special interest (including miscarriage and stillbirths of women not admitted to hospital) [37]. The proportions of women with an AE or a SAE were presented by treatment group with 95% confidence intervals and *p*-values were calculated by Fisher-exact text. For safety and tolerability outcomes it was considered that there was no evidence of significant difference between treatment groups if the 95% confidence intervals overlapped. Data analysis was performed using Stata statistical software version 13 (Stata Corp.).

## Results

### Baseline Characteristics of Study Participants

**Figure 1:**
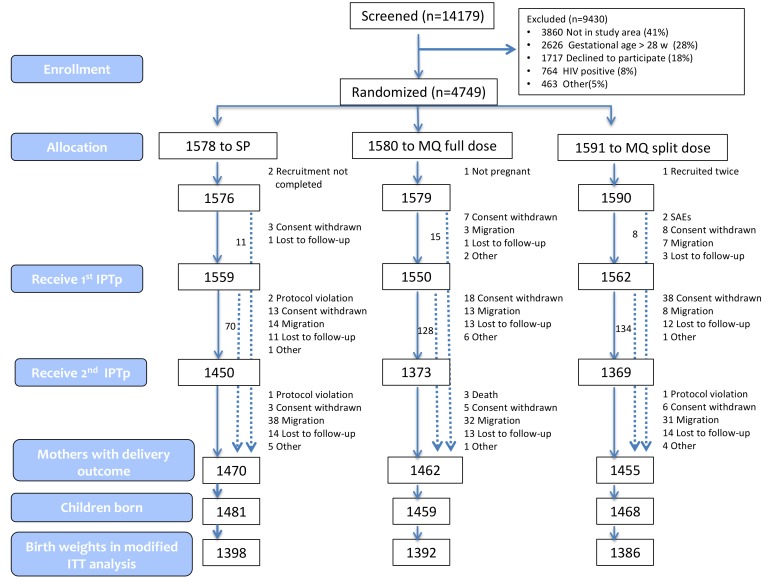
Trial profile (modified ITT cohort).

Figures 1 and S2 show the trial profile in the ITT and ATP cohorts, respectively. Overall, 4,749 pregnant women were randomized to receive IPTp (1,578 were allocated to SP, 1,580 to MQ full dose, and 1,591 to MQ split dose). Four women were not included in the ITT cohort: two did not finalize recruitment process, one was not pregnant, and one was recruited twice and only the first enrolment was included in the analysis. The main reasons for no enrolment into the trial were no permanent residence in the study area (41%) and gestational age>28 weeks (28%). The overall refusal rate for trial participation was 18%. Baseline characteristics were similar for women in the three treatment groups (Table 1). Syphilis prevalence was between 1% and 2% and anemia prevalence between 58% and 60%. Mean gestational age was 21 (standard deviation [SD] 7) weeks at the first IPTp dose and 26 (SD 6) weeks at the second IPTp dose. Median time between first and second dose was 35 (interquartile range [IQR] 12) days, and median time between the last dose and delivery was 94 days (IQR 48).

**Table 1:** Baseline characteristics.

Characteristics	SP	MQ Full Dose	MQ Split Dose
*N* Participants	1,576	1,579	1,590
Country^a^			
Benin	394	391	397
Gabon	391	394	395
Mozambique	392	395	396
Tanzania	399	399	402
Age (years)^b^	24.8 (6.3) [1,576]	24.7 (6.3) [1,579]	24.5 (6.0) [1,590]
Gravidity^a^			
Primigravidae	460 (29)	458 (29)	460 (29)
1–3 previous pregnancies	778 (49)	786 (50)	826 (52)
4 or more pregnancies	338 (21)	335 (21)	304 (19)
Weight (kg)^b^	59.9 (11.1) [1,576]	59.8 (11.0) [1,579]	59.6 (11.3) [1,590]
Height (cm)^b^	158.1 (8.0) [1,575]	158.3 (6.0) [1,578]	157.5 (8.5) [1,588]
MUAC index (cm)^b^	26.4 (3.5) [1,570]	26.5 (3.6) [1,574]	26.4 (3.6) [1,587]
Gestational age (weeks)^c^	21.0 (7.0) [1,575]	21.0 (7.0) [1,579]	21.0 (7.0) [1,590]
Gestational age in categories^a^			
First trimester	133 (8)	143 (9)	123 (8)
Second trimester	1,113 (71)	1,095 (69)	1,124 (71)
Third trimester	329 (21)	341 (22)	343 (22)
Literate^a^ (can read and/or write)	1,101 (70)	1,107 (70)	1,093 (69)
Syphilis test positive^a^	20 (1)	19 (1)	30 (2)
Hemoglobin (g/dl)^b^	10.6 (1.5) [1,572]	10.6 (1.5) [1,569]	10.5 (1.5) [1,585]
Overall anemia at baseline (Hb<11 g/dl)^a^	946 (60)	914 (58)	952 (60)

ITT cohort.

a *n* (column percentage).

b Arithmetic mean (SD) [*n*].

c Median (IQR) [*n*].

MUAC, middle upper arm circumference.

### Primary Endpoint

**Figure 2:**
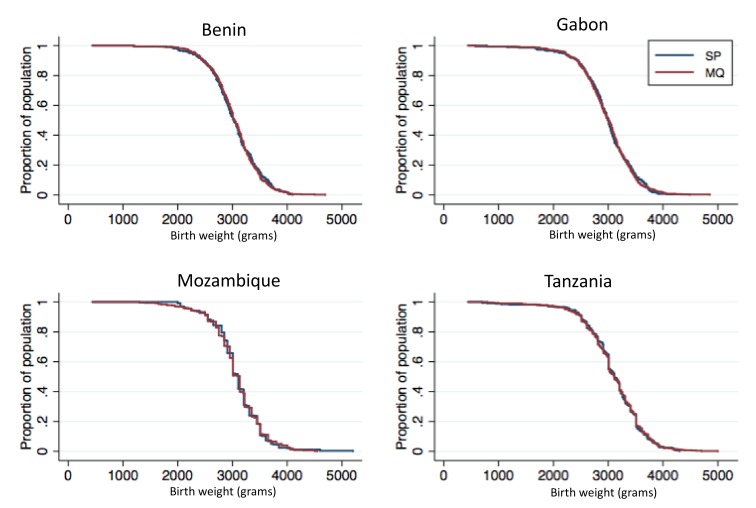
Birth weight distribution by study country and IPTp group. Newborn weights not captured at birth but within the first week of life were estimated using a linear regression model.

A total of 4,176 birth weights were collected and analyzed in the ITT cohort (81.4% were captured at birth; overall, 2.7% from stillbirths, 3.7% from twins), whereas 3,435 birth weights were analyzed in the ATP cohort (83.7% captured at birth; 2.5% from stillbirths, none from twins). There were no significant differences between the MQ and SP groups in either the prevalence of LBW infants (13.0% in the overall MQ group and 12.7% in the SP group, risk ratio [RR], 1.02 [95% CI 0.86–1.22; *p* = 0.80]) or in mean birth weight (Tables 2 and 3). On the basis of the confidence interval, non-inferiority could be accepted between the MQ and the SP groups at the pre-specified 25% margin for reduction in LBW prevalence in the ITT analysis, but not in the ATP analysis (Table 2). No difference in the prevalence of LBW was observed either between the full and split MQ dose (RR, 1.04 [95% CI 0.84–1.28]). The results were similar in the ATP adjusted (RR, 1.03 [95% CI 0.84–1.26]) and unadjusted (RR, 1.05 [95% CI 0.85–1.29]) analyses. Figure 2 shows the birth weight distribution by country and treatment group.

**Table 2:** Low birth weight (<2,500 g at birth) rates by treatment group and country.

Treatment Group and Country	SP		MQ		RR	95% CI	*p*-Value
	n/N	Percent	n/N	Percent			
Overall prevalence of LBW							
ITT	177/1,398	12.7	360/2,778	13.0	1.02	(0.86–1.22)	0.80
ATP	128/1,289	9.9	221/2,146	10.3	1.03	(0.84–1.26)	0.80
Benin							
ITT	47/349	13.5	110/703	15.6	1.16	(0.82–1.64)	0.39
ATP	33/322	10.2	70/629	11.1	1.06	(0.72–1.57)	0.77
Gabon							
ITT	54/331	16.3	112/652	17.2	1.05	(0.77–1.44)	0.75
ATP	36/291	12.4	52/384	13.5	1.04	(0.70–1.54)	0.85
Mozambique							
ITT	37/360	10.3	66/712	9.3	0.90	(0.60–1.36)	0.62
ATP	28/342	8.2	44/507	8.7	0.99	(0.64–1.55)	0.98
Tanzania							
ITT	39/358	10.9	72/711	10.1	0.93	(0.63–1.36)	0.71
ATP	31/334	9.3	55/626	8.8	0.95	(0.63–1.45)	0.83

ITT analysis adjusted by country. Interaction country×treatment: χ^2^: 1.22 with 3 degrees of freedom *p* = 0.766.

ATP analysis adjusted by baseline variables (country, seasonality, gestational age, gravidity, anemia, literacy, and middle upper arm circumference [MUAC]). Interaction country×treatment: χ^2^: 0.31 with 3 degrees of freedom *p* = 0.959.

**Table 3:** Malaria related outcomes at delivery.

Finding by Analysis	SP		MQ		RR or Difference	95% CI	*p*-Value
	*n*/*N*	Percent	*n*/*N*	Percent			
Birth weight, mean (SD);							
ITT	3,001.5 (517.8)		2,997.4 (535.5)		−4.1^a^	(−39.2 to 31.1)	0.82
ATP	3,036.7 (493.3)		3,037.3 (495.0)		2.4^a^	(−31.2 to 36.1)	0.89
Maternal parasitemia by OM)							
ITT	63/1,372	4.6	88/2,737	3.2	0.70	(0.51–0.96)	0.03
ATP	59/1,255	4.7	68/2,100	3.2	0.65	(0.46–0.91)	0.01
Parasitemia density (slides positive by OM), geometric mean [IQR]							
ITT	3,141 [1,032–11,763]		4,689 [1,049–35,902]		1.51^b^	(0.67–3.40)	0.32
ATP	3,018 [1,032–11,405]		5,468 [1,158–49,176]		2.14^b^	(0.88–5.19)	0.10
Placental infection (histology or smear)							
ITT	72/1,281	5.6	119/2,568	4.6	0.83	(0.63–1.10)	0.19
ATP	70/1,192	5.9	103/1,996	5.2	0.83	(0.62–1.11)	0.22
Maternal anemia (Hb<11 g/dl)							
ITT	609/1,380	44.1	1,110/2,743	40.5	0.92	(0.85–0.99)	0.03
ATP	543/1,258	43.2	833/2,104	39.6	0.95	(0.88–1.03)	0.24
Severe maternal anemia (Hb<7 g/dl)							
ITT	15/1,380	1.1	15/2,743	0.5	0.50	(0.25–1.02)	0.06
ATP	8/1,258	0.6	12/2,104	0.6	1.04	(0.43–2.51)	0.93
Maternal Hb, mean (SD)[*n*]							
ITT	11.0 (1.6) [1,380]		11.1 (1.5) [2,743]		0.15^a^	(0.05–0.25)	0.003
ATP	11.0 (1.5) [1,258]		11.2 (1.5) [2,104]		0.10^a^	(−0.00 to 0.20)	0.06
Cord blood parasitemia by OM							
ITT	4/1,337	0.3	6/2,667	0.2	0.74	(0.21–2.62)	0.64
ATP^c^	3/1,219	0.2	5/2,060	0.2	0.92	(0.21–4.01)	0.92
Cord blood anemia (Hb<12.5 g/dl)							
ITT	170/1,334	12.7	353/2,672	13.2	1.03	(0.87–1.22)	0.71
ATP	150/1,218	12.3	246/2,061	11.9	1.02	(0.84–1.22)	0.86
Maternal parasitemia by OM 1 month after delivery							
ITT	21/1,149	1.8	42/2,281	1.8	1.01	(0.60–1.69)	0.98
ATP	21/1,052	2.0	32/1,788	1.8	0.86	(0.49–1.50)	0.60

a Arithmetic difference.

b Proportional difference; ATP analysis adjusted by baseline variables (country, seasonality, gestational age, gravidity, anemia, literacy, and middle upper arm circumference [MUAC]).

c ATP analysis adjusted only by country.

OM, observed microscopy.

### Secondary Endpoints

The risk of maternal peripheral malaria parasitemia at delivery was 30% lower in women who received MQ compared to those who received SP (RR, 0.70 [95% CI 0.51–0.96]; *p* = 0.03). The risk of overall maternal anemia (Hb<11 g/dl) at delivery was also lower (44.1% in the SP group versus 40.5% in the MQ group [RR, 0.92 (95% CI 0.85–0.99)]; *p* = 0.03) and the mean Hb was higher in women who received IPTp-MQ compared to those in the SP group, although these differences were only significant in the ITT analysis (Table 3). The frequency of severe anemia was also lower in women receiving MQ although the difference did not reach statistical significance (Table 3).

**Figure 3:**
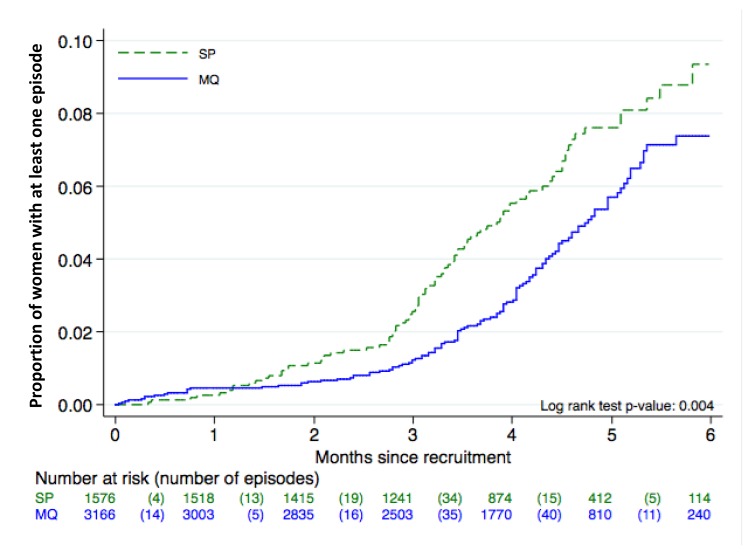
Time to first episode of clinical malaria. ITT cohort. Kaplan Meier graph.

There were no differences between groups in the prevalence of placental infection, neonatal parasitemia, neonatal anemia, or maternal peripheral parasitemia one month after delivery (Table 3). Results stratified by country can be found in Tables S3, S4, S5 and placental histology results by treatment in Table S6. The incidence of clinical malaria episodes and of all-cause outpatient attendances during pregnancy were significantly lower in women receiving IPTp-MQ compared to those receiving IPTp-SP (RR, 0.67 [95% CI 0.52–0.88]; *p* = 0.004 and RR, 0.86 [0.78–0.95]; *p* = 0.003, respectively) (Figure 3; Table 4). The incidence of all-cause hospital admissions was lower in the MQ group although not statistically significantly different (RR, 0.88 [0.68–1.14]; *p* = 0.35) (Table 4). The overall reported use of LLITN at delivery was of 90% and of 96% one month after the end of pregnancy, with no difference between study groups.

**Table 4:** Incidence of clinical malaria, outpatients visits, and hospital admissions.

Incidences	SP *N*/PYAR	Incidence^a^	MQ *N*/PYAR	Incidence^a^	Relative Rate	95% CI	*p*-Value
Clinical malaria							
ITT	96/551.8	0.17	130/1,103.2	0.12	0.67	(0.52–0.88)	0.004
ATP	80/471.4	0.17	86/788.0	0.11	0.64	(0.47–0.87)	0.004
Outpatients visits							
ITT	850/557.8	1.52	1,480/1,110.1	1.33	0.86	(0.78–0.95)	0.003
ATP	709/476.3	1.49	1,030/792.5	1.30	0.88	(0.79–0.98)	0.02
All-cause hospital admissions							
ITT	106/557.8	0.19	186/1,110.1	0.17	0.88	(0.68–1.14)	0.35
ATP	67/476.3	0.14	99/792.5	0.13	0.85	(0.61–1.18)	0.33
Non-obstetric hospital admission							
ITT	91/557.8	0.16	166/1,110.1	0.15	0.92	(0.69–1.22)	0.55
ATP	61/476.3	0.13	84/792.5	0.11	0.79	(0.55–1.13)	0.20

a Episodes person/year; ATP analysis adjusted by baseline variables (country, seasonality, gestational age, gravidity, anemia, literacy, and middle upper arm circumference [MUAC]); ITT analysis adjusted by country.

### Safety

There was no difference in the prevalence of adverse pregnancy outcomes (including miscarriages, stillbirths, and congenital malformations) between groups (Table 5). The number of SAEs, including maternal and neonatal deaths, was also similar among the three study arms. The number of women who had SAEs considered as drug-related by the site investigator was higher in the MQ groups: one in the SP group (0.1%; a miscarriage), 11 in the MQ full-dose group (0.7%; one urinary tract infection, one generalized urticaria, one stillbirth, one premature delivery, two miscarriages, and five vomiting episodes), and ten in the MQ split-dose group (0.6%; two miscarriages, two stillbirths, three preterm delivery, one malaria, and three vomiting episodes). Serious adverse pregnancy outcomes considered as drug-related by the study investigators were carefully reviewed by the trial's independent DSMB, which concluded that a causal relationship between the drug and these SAEs could not be established. No serious neurological AEs were reported among study participants. The frequency of non-serious reported sleeping disorders was higher in the MQ group (79/3,113, 2.5%; [95% CI 2.0–3.2]) than in the SP group (12/1,561, 0.8% [95% CI 0.4–1.3]). Two women with unknown psychiatric antecedents attempted suicide in the SP group.

**Table 5:** Adverse pregnancy outcomes and serious adverse events by study arm (safety cohort).

Adverse Outcomes and SAEs	SP			MQ Full Dose			MQ Split Dose			*p*-Value
	*n*	Percent	95% CI	*n*	Percent	95% CI	*n*	Percent	95% CI	
**Adverse pregnancy outcomes**										
Miscarriages^a^	12	0.77	(0.40–1.34)	14	0.90	(0.49–1.51)	11	0.70	(0.35–1.26)	0.78
Stillbirths^b^	44	2.82	(2.06–3.77)	54	3.48	(2.63–4.52)	47	3.01	(2.22–3.98)	0.56
Congenital malformations	15	1.02	(0.57–1.67)	14	0.97	(0.53–1.61)	11	0.75	(0.38–1.34)	0.75
Prematurity^c^^,,^ d	56	4.94	(3.75–6.37)	56	5.02	(3.81–6.47)	62	5.49	(4.24–6.99)	0.82
**SAEs**										
Any SAE	140	8.97	(7.60–10.50)	151	9.74	(8.31–11.32)	124	7.94	(6.65–9.39)	0.21
SAEs related to medication	1	0.06	(0.00–0.36)	11	0.71	(0.35–1.27)	10	0.64	(0.31–1.17)	0.006
Maternal deaths	4	0.26	(0.07–0.65)	6	0.39	(0.14–0.84)	3	0.19	(0.04–0.56)	0.51
Neonatal deaths	31	2.04	(1.39–2.89)	31	2.07	(1.41–2.93)	31	2.04	(1.39–2.88)	1.00

a Miscarriage: termination of pregnancy and expulsion of an embryo or of a foetus prior to 20 complete weeks of gestation and/or a birth weight less than 500 g.

b Stillbirth: foetal death that occurs after 20 complete weeks of gestation.

c Prematurity: birth before the beginning of the 37th week (assessed by the Ballard score).

d Excluding incomplete data on Ballard score.

### Tolerability

**Figure 4:**
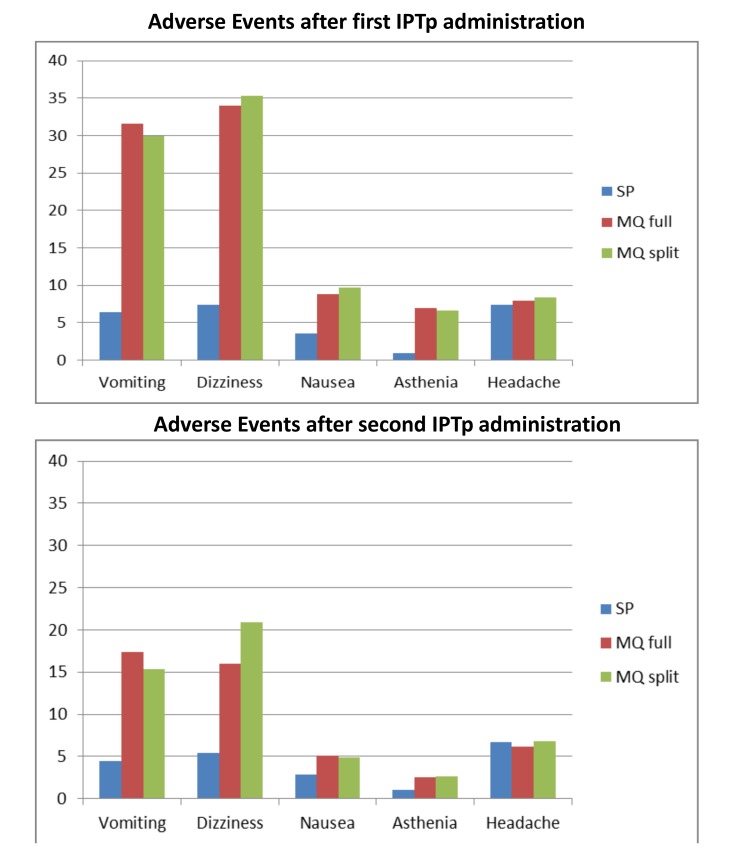
Reported medication-related adverse events.

The immediate tolerability of IPTp was poorer in the two MQ groups as compared to the SP group, with no difference between the full and split-dose groups (Table 6). The most frequently reported related AEs following the first MQ administration were dizziness (33.9% and 35.5% in the full and split-dose groups, respectively) and vomiting (31.7% and 30.1% in the full and split dose groups, respectively) (Table 7). The majority of these AEs started within 48 hours after drug administration. The mean duration of the aforementioned AEs was one day (IQR 2) and over 70% were classified as mild (Table 8). The prevalence of dizziness and vomiting related to MQ was reduced at the second IPTp administration compared to the first one (Figure 4; Table 7).

**Table 6:** Immediate tolerability (safety cohort).

Adverse Events	SP			MQ Full Dose			MQ 1st Split Dose			MQ 2nd Split Dose		
	*n*	Percent	95% CI	*n*	Percent	95% CI	*n*	Percent	95% CI	*n*	Percent	95% CI
**1st IPTp administration**												
Vomiting within 30 min	0	0.00	(0.00–0.24)	16	1.05	(0.60–1.69)	6	0.39	(0.14–0.85)	13	0.89	(0.48–1.52)
Vomiting within 60 min	1	0.06	(0.00–0.36)	12	0.78	(0.41–1.37)	2	0.13	(0.02–0.47)	6	0.41	(0.15–0.90)
Vomiting replacement dose	0	0.00	(0.00–0.24)	9	0.59	(0.27–1.11)	0	0.00	(0.00–0.24)	3	0.21	(0.04–0.06)
**2nd IPTp administration**												
Vomiting within 30 min	1	0.07	(0.00–0.38)	10	0.73	(0.35–1.35)	4	0.29	(0.08–0.75)	3	0.24	(0.05–0,70)
Vomiting within 60 min	0	0.00	(0.00–0.25)	10	0.73	(0.35–1.35)	1	0.07	(0.00–0.41)	2	0.16	(0.02–0.57)
Vomiting replacement dose	1	0.07	(0.00–0.38)	3	0.22	(0.05–0.64)	1	0.07	(0.00–0.41)	1	0.08	(0.00–0.44)

**Table 7:** Most frequent medication related adverse events (safety cohort).

Adverse Events	SP			MQ Full Dose			MQ Split Dose		
	*n*	Percent	95% CI	*n*	Percent	95% CI	*n*	Percent	95% CI
**1st IPTp administration**									
Dizziness	115	7.38	(6.13–8.79)	526	33.94	(31.58–36.35)	554	35.47	(32.09–37.90)
Vomiting	100	6.41	(5.25–7.75)	491	31.68	(29.37–34.06)	471	30.15	(27.88–32.50)
Headache	115	7.38	(6.13–8.79)	123	7.94	(6.64–9.39)	131	8.39	(7.06–9.87)
Nausea	55	3.53	(2.67–4.57)	136	8.77	(7.41–10.29)	152	9.73	(8.31–11.31)
Asthenia	14	0.90	(0.49–1.50)	107	6.90	(5.69–8.28)	104	6.66	(5.47–8.01)
**2nd IPTp administration**									
Dizziness	78	5.38	(4.28–6.67)	221	16.10	(14.19–18.15)	284	20.75	(18.63–22.99)
Vomiting	65	4.48	(3.48–5.68)	239	17.41	(15.44–19.52)	209	15.27	(13.40–17.28)
Headache	97	6.69	(5.46–8.10)	84	6.12	(4.91–7.52)	93	6.79	(5.52–8.26)
Nausea	41	2.83	(2.04–3.82)	70	5.10	(4.00–6.40)	67	4.89	(3.81–6.17)
Asthenia	15	1.03	(0.58–1.70)	35	2.55	(1.78–3.53)	37	2.70	(1.91–3.71)

**Table 8:** Severity of reported vomiting and dizziness by treatment group.

Grade of Severity	SP		MQ Full		MQ Split	
	*n*	Percent	*n*	Percent	*n*	Percent
**Vomiting related to medication**						
Mild^a^	145	86.31	511	68.68	497	71.10
Moderate^b^	22	13.10	194	26.08	186	26.61
Severe^c^	1	0.60	39	5.24	16	2.29
**Dizziness related to medication**						
Mild	176	88.44	559	72.98	660	76.57
Moderate	21	10.55	175	22.85	185	21.46
Severe	2	1.01	32	4.18	17	1.97

a Mild: awareness of sign or symptom, but easily tolerated.

b Moderate: discomfort enough to cause interference with usual activity.

c Severe: incapacitating with inability to work or perform usual activity or patients at risk of death at the time of the event.

Table 9 shows the reported AEs by system organ class and treatment group and Table 10 the list of SAEs related to the drug.

**Table 9:** Adverse events by system organ class and treatment group.

Adverse Events	SP *N* = 1,561			MQ Full *N* = 1,551			MQ Split *N* = 1,562		
	*n*	Percent	(95% CI)	*n*	Percent	(95% CI)	*n*	Percent	(95% CI)
Blood and lymphatic system disorders	270	17.30	(15.45–19.27)	245	15.80	(14.02–17.71)	249	15.94	(14.16–17.85)
Cardiac disorders	16	1.02	(0.59–1.66)	23	1.48	(0.94–2.22)	25	1.60	(1.04–2.35)
Congenital, familial, and genetic disorders	0	0.00	(0.00–0.24)	2	0.13	(0.02–0.47)	0	0.00	(0.00–0.24)
Ear and labyrinth disorders	7	0.45	(0.18–0.92)	8	0.52	(0.22–1.01)	12	0.77	(0.40–1.34)
Eye disorders	6	0.38	(0.14–0.83)	2	0.13	(0.02–0.47)	9	0.58	(0.26–1.09)
Gastrointestinal disorders	360	23.06	(20.99–25.23)	757	48.81	(46.29–51.33)	715	45.77	(43.28–48.28)
General disorders and administration site conditions	590	37.80	(35.38–40.25)	903	58.22	(55.72–60.69)	948	60.69	(58.22–63.12)
Immune system disorders	8	0.51	(0.22–1.01)	4	0.26	(0.07–0.66)	2	0.13	(0.02–0.46)
Infections and infestations	166	10.63	(9.15–12.27)	111	7.16	(5.92–8.55)	124	7.94	(6.65–9.39)
Injury, poisoning, and procedural complications	1	0.06	(0.00–0.36)	4	0.26	(0.07–0.66)	0	0.00	(0.00–0.24)
Musculoskeletal and connective tissue disorders	77	4.93	(3.91–6.13)	79	5.09	(4.05–6.31)	87	5.57	(4.48–6.83)
Neoplasms benign, malignant and unspecified (incl cysts and polyps)	1	0.06	(0.00–0.36)	0	0.00	(0.00–0.24)	0	0.00	(0.00–0.24)
Nervous system disorders	22	1.41	(0.89–2.13)	46	2.97	(2.18–3.94)	54	3.46	(2.61–4.49)
Pregnancy, puerperium, and perinatal conditions	158	10.12	(8.67–11.73)	159	10.25	(8.79–11.87)	152	9.73	(8.31–11.31)
Psychiatric disorders	2	0.13	(0.02–0.46)	0	0.00	(0.00–0.24)	0	0.00	(0.00–0.24)
Renal and urinary disorders	60	3.84	(2.95–4.92)	50	3.22	(2.40–4.23)	46	2.94	(2.16–3.91)
Reproductive system and breast disorders	268	17.17	(15.33–19.13)	221	14.25	(12.55–16.09)	237	15.17	(13.43–17.05)
Respiratory, thoracic, and mediastinal disorders	164	10.51	(9.03–12.13)	122	7.87	(6.57–9.32)	156	9.99	(8.54–11.58)
Skin and subcutaneous tissue disorders	26	1.67	(1.09–2.43)	24	1.55	(0.99–2.29)	16	1.02	(0.59–1.66)
Vascular disorders	23	1.47	(0.94–2.20)	11	0.71	(0.35–1.27)	14	0.90	(0.49–1.50)

N, number of participants with at least one event.

**Table 10:** List of possibly/probably related SAEs.

Number	Treatment	Causality	MEDdra_code
1	MQ Full	Possible	Urinary tract infection
2	MQ Split	Possible	Spontaneous abortion
3	MQ Split	Possible	Stillbirth
4	MQ Split	Possible	Stillbirth
5	MQ Full	Possible	Generalized urticarial
6	MQ Full	Possible	Stillbirth
7	MQ Full	Possible	Other preterm infants, 500–749 g
8	MQ Split	Possible	Other preterm infants, 500–749 g
9	MQ Full	Possible	Spontaneous abortion
10	MQ Full	Probable	Vomiting
11	MQ Full	Probable	Vomiting
12	MQ Split	Probable	Vomiting
13	MQ Split	Probable	Malaria
14	MQ Split	Probable	Vomiting
15	MQ Full	Probable	Vomiting
16	MQ Full	Probable	Vomiting
17	SP	Possible	Spontaneous abortion
18	MQ Split	Probable	Vomiting
19	MQ Full	Probable	Vomiting
20	MQ Full	Possible	Spontaneous abortion
21	MQ Split	Possible	Other preterm infants, 500–749 g
22	MQ Split	Possible	Spontaneous abortion
23	MQ Split	Possible	Other preterm infants, 500–749 g

### Adherence with IPTp

The second IPTp administration was not given to 7% (111/1,559), 11% (178/1,550), and 12% (193/1,562) of women who had received the first administration of SP, MQ full, and split-dose, respectively. In addition, in the MQ split-dose group, 7% (101/1,562) and 8% (108/1,369) of the women did not receive the second half dose at the first and second IPTp administrations, respectively. The proportion of women who had a related AE within two weeks after receiving the first IPTp administration and who did not receive the second IPTp administration was 7% (23/293), 16% (116/721), and 14% (109/769) in the SP, MQ full, and split dose groups, respectively.

## Discussion

This multicentre open-label randomized trial that compared two doses of IPTp with MQ (15 mg/kg dose) versus SP in HIV-negative pregnant women using LLITNs found no differences in the prevalence of LBW between the two intervention groups. However, the prevalences of maternal parasitemia and anemia at delivery were significantly lower in women receiving MQ compared to SP recipients. The study also found that the incidence of clinical malaria and all-cause outpatient visits during pregnancy was reduced in the MQ group. On the other hand, the tolerability of MQ was poorer compared to that of SP especially for the AEs that have been typically related with this drug such as dizziness and vomiting.

The lack of beneficial effect of MQ as compared to SP in reducing the prevalence of LBW might be explained by the fact that all women were protected by efficacious malaria control strategies [38], together with a decreased malaria transmission during the study period in some study sites. These factors resulted in a reduced exposure to the parasite reflected by the low prevalence of peripheral parasitemia at delivery and placental infection in all intervention groups. These circumstances may have decreased the contribution of malaria (which is one of the many factors affecting birth weight in endemic areas) to LBW, compromising the statistical power to detect differences between groups. Some previous chemoprophylaxis and IPT trials in pregnant women have documented a lack of effect on birth weight of these malaria preventive strategies [9],[23],[39]. Another potential explanation for diluting the difference in birth weight outcomes might be that women were on average two months without the protection of an antimalarial since the last IPTp administration until delivery. This possibility would support the addition of more IPTp administrations as it is now more clearly recommended [7]. Since LBW is only one of the deleterious consequences of malaria in pregnancy and is prone to multiple confounding, it can be argued that other study outcomes might be better suited to reflect the efficacy of malaria control strategies in pregnant women [7]. This consideration suggests the need to shift the endpoint in the assessment of the impact of malaria control measures in pregnancy to more upstream malaria-related outcomes than birth weight, such as parasite prevalence and incidence of clinical episodes. In this study there were significant differences in the prevalence of parasitemia and overall anemia at delivery, which were lower in women who had received MQ compared to those who had received SP. Importantly, the incidence of clinical malaria episodes and overall outpatient visits during pregnancy were also significantly reduced in the two MQ arms. These two outcomes are frequently overlooked in the evaluation of malaria control strategies in pregnant women in the assumption that the public health benefit should be only or mainly reflected on the effects on the newborn. Differences in the magnitude of reduction in the incidence of clinical malaria and outpatient visits have also been reported in malaria prevention trials in children [40]. Interestingly, the figure showing the incidence of clinical malaria during pregnancy indicates that the risk of malaria rises after the second IPTp administration, and that a third IPTp administration would be beneficial to improve protection against the infection, which supports the new policy for IPTp [7].

A finding that has generated much concern and debate in relation with the use of MQ in pregnancy was the report from a retrospective analysis in Thailand documenting an increased risk of stillbirths in 208 pregnant women treated with MQ [21]. This observation was not confirmed in larger randomized trials and descriptive studies [22],[41],[42]. In the present study, the safety profile of IPTp-MQ in terms of the number of SAEs and adverse pregnancy outcomes was similar to that of SP. The observed discrepancy between the investigator's assessment of the drug relatedness of serious adverse pregnancy outcomes and the final judgment made by the trial's DSMB is most likely due to the open label design of the study, which may have influenced the assignment of causality made by the clinical investigator.

Given the lack of an appropriately validated tool for rural African populations, a detailed screening of neurological and psychological problems was not carried out as part of the study. Although there were no reports of depression or other serious neurological problems, it cannot be ruled out that they may have occurred. The only serious adverse psychiatric events reported in the study were suicide attempts in two women who had received SP. The other frequently reported AE associated with MQ exposure among individuals travelling in malaria endemic regions on weekly chemoprophylaxis [43]–[46] is sleeping disorders. In this study, 82 women in the MQ groups and 12 in the SP group reported sleeping disorders (such as insomnia and bad dreams). As with the above-mentioned related AEs, it is difficult to disentangle whether these reports indeed reflect a side effect, or alternatively can be explained by a potential bias due to the open trial design [47]. Previous studies using the same MQ dose (15 mg/kg) have found the same trend although the differences between treatments were not statistically significant [23].

In agreement with a recent IPTp trial, MQ presented a poorer tolerability than SP with higher frequencies of related AEs such as dizziness and vomiting [23]. While the frequency of other AEs not previously related to MQ such as headache were similar after both IPTp administrations, the frequency of dizziness, vomiting, nausea, and weakness decreased after subsequent MQ doses. This finding could be due to a selection of the women more susceptible to experience dizziness and vomiting not receiving the second MQ administration. However a reduced frequency of related AEs with subsequent doses has been observed in previous chemoprophylaxis and IPT trials with MQ in pregnancy, as well as in reports from non-pregnant individuals travelling in malaria endemic regions indicating that a true tolerance effect might play a role [23],[45],[48].

Unlike previous studies investigating different MQ regimens, splitting the administration of MQ over two consecutive days did not translate into a better tolerability of the drug, although the antimalarial efficacy was maintained [20]. In addition, adherence with IPTp-MQ was considerably lower in those women assigned to the split dose. This finding strongly emphasizes that future candidate drugs for IPTp should be evaluated as single dose administration with observed intake to ensure optimal adherence in otherwise asymptomatic pregnant women.

The prevalence of syphilis was very low in the study participants, which may reflect an improved effectiveness of ANC syphilis control programs [49], and it is possibly accompanied by a reduction in the prevalence of other sexually transmitted infections in pregnancy. This finding may be helpful in the discussion and evaluation of alternatives to SP with more complex drug combinations that have the objective of concomitantly treating sexually transmitted diseases while preventing malaria [50].

In this study, and following the same rationale as for SP, the dosage of 15 mg/kg of MQ was chosen as it is recommended for malaria treatment in areas of low parasite resistance [23]. A dosage of MQ of 125 mg used in a previous weekly chemoprophylaxis study in Karen pregnant women showed comparable tolerability to the placebo group [39]. It is possible that a lower dosage than that used in this study would have been better tolerated maintaining similar antimalarial efficacy.

The main limitation of this study is its open-label design, which may have led to biases in the assessment of outcomes, especially those of safety assessing adverse effects commonly ascribed to the study drugs [51]. However, given the differences in scheme and dosage between treatments, a double-blind design was considered to be difficult and not acceptable for the participants given the large quantity of tablets the women would have needed to take.

From the study results, it can be concluded that women taking two IPTp administrations of MQ at the treatment dosage of 15 mg/kg, and in the context of high LLITN use, had similar prevalence rates of LBW as women taking SP. MQ recipients had less clinical malaria than SP recipients and the pregnancy outcomes and safety profile were similar. MQ tolerability was poorer than that of SP even when splitting the dose over two days. These results do not support a change in the current recommended IPTp policy [7],[52].

## Supporting Information

Figure S1 **Estimation of newborn weights not captured at birth using a regression model.**
(TIF)

Figure S2 **Trial profile (ATP cohort).**
(TIF)

Table S1 **Local regulatory authorities and national ethical review boards.**
(DOC)

Table S2 **Characteristics of MiPPAD study sites.**
(DOC)

Table S3 **Maternal parasitemia at delivery by country.**
(DOCX)

Table S4 **Maternal anemia at delivery by country.**
(DOCX)

Table S5 **Placental infection by country.**
(DOCX)

Table S6 **Placental histology by treatment.**
(DOCX)

Table S7 **Results showing the Number Needed to Treat (NNT) or Harm (NNH) by outcome.**
(DOCX)

Text S1 **MiPPAD study protocol.**
(PDF)

Text S2 **CONSORT checklist.**
(DOC)

Text S3 **Statistical analysis plan.**
(PDF)

Text S4 **ClinicalTrials.gov registration receipt.**
(PDF)

## Abbreviations

AE: adverse event
ANC: antenatal care
ATP: according to protocol
Hb: hemoglobin
IPTp: intermittent preventive treatment in pregnancy
IQR: interquartile range
ITT: intention to treat
LBW: low birth weight
LLITN: long lasting insecticide treated nets
MQ: mefloquine
PYAR: person/year
RR: risk ratio
SAE: serious adverse event
SD: standard deviation
SP: sulfadoxine-pyrimethamine
