# Supplementary figures and images for: Intermittent Preventive Treatment of Malaria in Pregnancy with Mefloquine in HIV-Negative Women: A Multicentre Randomized Controlled Trial
Source: PLoS Med. 2014 Sep 23;11(9):e1001733. doi: 10.1371/journal.pmed.1001733 (PMC4172436; doi:10.1371/journal.pmed.1001733)

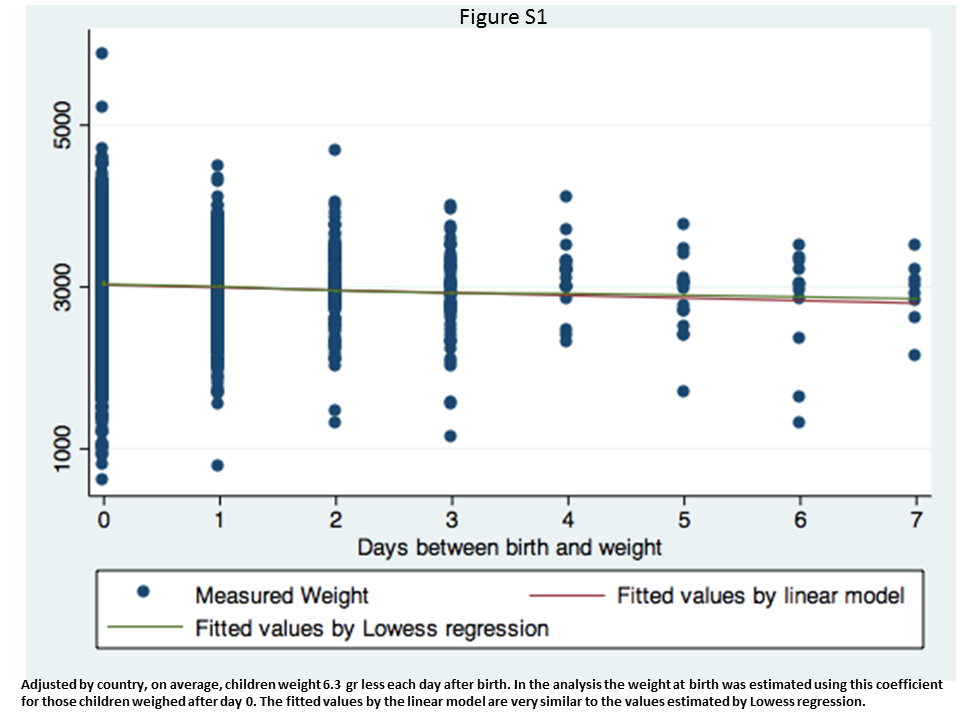

Supplement: Figure S1 — Estimation of newborn weights not captured at birth using a regression model. (TIF) [file pmed.1001733.s001.tif]

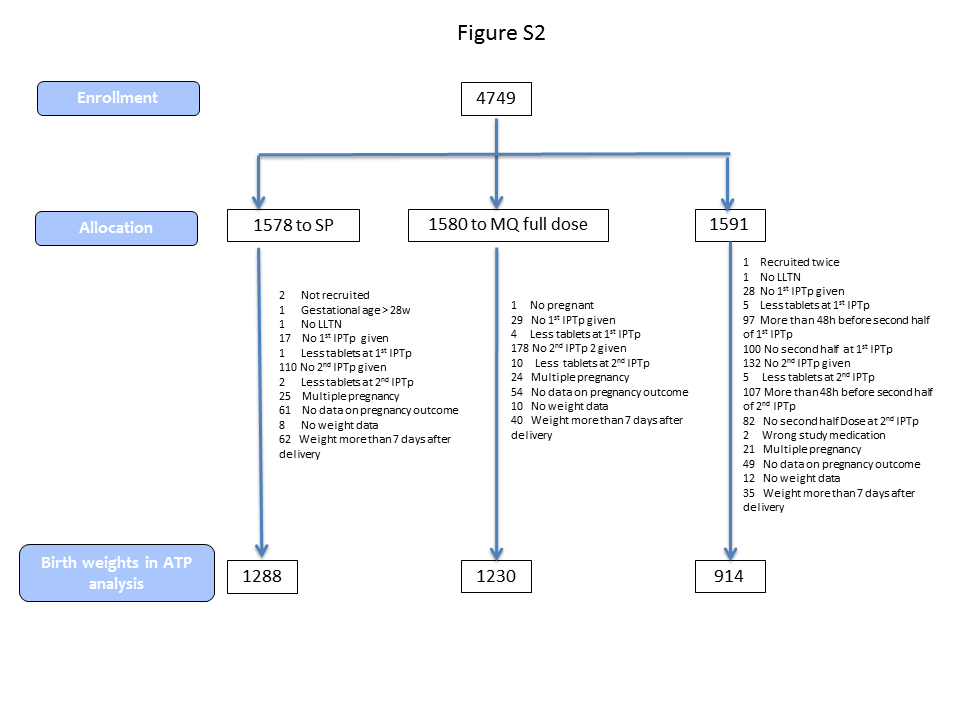

Supplement: Figure S2 — Trial profile (ATP cohort). (TIF) [file pmed.1001733.s002.tif]
