# Supplementary material for: Intermittent Preventive Treatment of Malaria in Pregnancy with Mefloquine in HIV-Negative Women: A Multicentre Randomized Controlled Trial
Source: PLoS Med. 2014 Sep 23;11(9):e1001733. doi: 10.1371/journal.pmed.1001733 (PMC4172436; doi:10.1371/journal.pmed.1001733)
Supplement: Table S1 — Local regulatory authorities and national ethical review boards. (DOC) [file pmed.1001733.s003.doc]

**Table S1. Local regulatory authorities and national ethical review boards**

| **Country** | **Review Board** |
| --- | --- |
| **Benin** | - Comité d’ Ethique de l’Université Abomey Calavi |
| **Gabon** | - Comité d’Ethique Régional Indépendant de Lambaréne |
| **Mozambique** | - Comité Nacional de Bioética para a Saúde (Ministério da Saúde) - Departamento Farmacéutico (Ministério da Saúde) |
| **Tanzania** | - Institutional Review Board - National Institute for Medical Research Review Board - Tanzania Food and Drug Association |
