# Supplementary material for: Intermittent Preventive Treatment of Malaria in Pregnancy with Mefloquine in HIV-Negative Women: A Multicentre Randomized Controlled Trial
Source: PLoS Med. 2014 Sep 23;11(9):e1001733. doi: 10.1371/journal.pmed.1001733 (PMC4172436; doi:10.1371/journal.pmed.1001733)
Supplement: Table S2 — Characteristics of MiPPAD study sites. (DOC) [file pmed.1001733.s004.doc]

**Table S2. Characteristics of MiPPAD study sites**

| **Country** | **Benin** | **Gabon** | **Mozambique** | **Tanzania** |
| --- | --- | --- | --- | --- |
| **Sites** | - Allada - Sékou - Attogon | - Lambaréné - Fougamou | - Manhiça - Maragra | - Makole - Chamwino |
| **Malaria Transmission** | Hyperendemic | Hyperendemic | Mesoendemic | Mesoendemic |
| **High season** | Apr-Jul | Oct-May | Sep-Mar | Jun-Aug |
| ***P. falciparum* infection** | >90% | >90% | >90% | >90% |
| **SP resistance markers** | 85%^1^ | >80%^2^ | 12%^3^ | <25%^4^ |
| **HIV tests** | - Determine (Alere Organics, France) - SD Bioline (Umhlanga, South Africa) | - Determine (Abbot Lab, USA) - Immunocomb (Scanbur, Denmark) | - Determine (Abbot Lab, USA) - Unigold (TM HIV, Trinity Biotech, Ireland) | - Determine (Abbott Lab, USA) - SD Bioline HIV (Standard Diagnostics, Korea) |
| **Syphilis tests** | - RPR (Biotec, UK) | - Abbott Determine Rapid Syphilis TP | - SD Bioline Syphilis 3.0 (Standard Diagnostics, Korea) | - SD Bio line (Abon Biopharm, Hangzhuo, Co. Ltd) |

^1^Prevalence of triple mutations in pregnant women, data from 2006 ^2^ Prevalence of quintuple mutations in pregnant women, data from 2011; ^3^Prevalence of quintuple mutations in pregnant women, data from 2006; ^4^ Prevalence of triple mutations in children, data from 2001.
