# Supplementary material for: Intermittent Preventive Treatment of Malaria in Pregnancy with Mefloquine in HIV-Negative Women: A Multicentre Randomized Controlled Trial
Source: PLoS Med. 2014 Sep 23;11(9):e1001733. doi: 10.1371/journal.pmed.1001733 (PMC4172436; doi:10.1371/journal.pmed.1001733)
Supplement: Table S3 — Maternal parasitemia at delivery by country. (DOCX) [file pmed.1001733.s005.docx]

| Country | **SP** | | **MQ** | | **RR^1^** | **95%CI** | **p-value** |
| --- | --- | --- | --- | --- | --- | --- | --- |
|  | **n/N** | **%** | **n/N** | **%** |  |  |  |
| Benin | 34/328 | 10.4 | 50/661 | 7.6 | 0.73 | (0.48; 1.11) | 0.137 |
| Gabon | 19/329 | 5.8 | 25/634 | 3.9 | 0.68 | (0.38; 1.22) | 0.199 |
| Mozambique | 10/364 | 2.7 | 13/737 | 1.8 | 0.64 | (0.28; 1.45) | 0.287 |
| Tanzania | 0/351 | 0.00 | 0/705 | 0.00 | - | - | - |

Table S3. Maternal parasitaemia at delivery by treatment and country (ITT)

^1^Relative Risk. ITT analysis adjusted by country. Interaction Country x Treatment: χ^2^ :4.29 with 3 degrees of freedom p=0.232.
