# Supplementary material for: Intermittent Preventive Treatment of Malaria in Pregnancy with Mefloquine in HIV-Negative Women: A Multicentre Randomized Controlled Trial
Source: PLoS Med. 2014 Sep 23;11(9):e1001733. doi: 10.1371/journal.pmed.1001733 (PMC4172436; doi:10.1371/journal.pmed.1001733)
Supplement: Table S4 — Maternal anemia at delivery by country. (DOCX) [file pmed.1001733.s006.docx]

Table S4. Maternal anemia (Hb <11 g/dl) at delivery by treatment and country (ITT)

| **Country** | **SP** | | **MQ** | | **RR^1^** | **95%CI** | **p-value** |
| --- | --- | --- | --- | --- | --- | --- | --- |
|  | **n/N** | **%** | **n/N** | **%** |  |  |  |
| Benin | 137/333 | 41.1 | 256/664 | 38.6 | 0.94 | (0.80; 1.10) | 0.428 |
| Gabon | 171/331 | 51.7 | 314/637 | 49.3 | 0.95 | (0.84; 1.09) | 0.482 |
| Mozambique | 160/363 | 44.1 | 286/736 | 38.9 | 0.88 | (0.76; 1.02) | 0.093 |
| Tanzania | 141/353 | 39.9 | 254/706 | 36.0 | 0.90 | (0.77; 1.06) | 0.204 |

^1^Relative Risk. ITT analysis adjusted by country. Interaction Country x Treatment: χ^2^ :0.74 with 3 degrees of freedom p=0.864.
