# Supplementary material for: Intermittent Preventive Treatment of Malaria in Pregnancy with Mefloquine in HIV-Negative Women: A Multicentre Randomized Controlled Trial
Source: PLoS Med. 2014 Sep 23;11(9):e1001733. doi: 10.1371/journal.pmed.1001733 (PMC4172436; doi:10.1371/journal.pmed.1001733)
Supplement: Table S5 — Placental infection by country. (DOCX) [file pmed.1001733.s007.docx]

Table S5. Placental infection by treatment and country (ITT)

| **Country** | **SP** | | **MQ** | | **RR^1^** | **95%CI** | **p-value** |
| --- | --- | --- | --- | --- | --- | --- | --- |
|  | **n/N** | **%** | **n/N** | **%** |  |  |  |
| Benin | 42/289 | 14.5 | 62/578 | 10.7 | 0.74 | (0.51; 1.06) | 0.104 |
| Gabon | 15/313 | 4.8 | 30/611 | 4.9 | 1.02 | (0.56; 1.88) | 0.938 |
| Mozambique | 10/333 | 3.0 | 14/682 | 2.1 | 0.68 | (0.31; 1.52) | 0.352 |
| Tanzania | 5/346 | 1.4 | 13/697 | 1.9 | 1.29 | (0.46; 3.59) | 0.625 |

^1^Relative Risk. ITT analysis adjusted by country. Interaction Country x Treatment: χ^2^ :1.79 with 3 degrees of freedom p=0.617.
