# Supplementary material for: Intermittent Preventive Treatment of Malaria in Pregnancy with Mefloquine in HIV-Negative Women: A Multicentre Randomized Controlled Trial
Source: PLoS Med. 2014 Sep 23;11(9):e1001733. doi: 10.1371/journal.pmed.1001733 (PMC4172436; doi:10.1371/journal.pmed.1001733)
Supplement: Table S6 — Placental histology by treatment. (DOCX) [file pmed.1001733.s008.docx]

**Table S6. Placental histology results by treatment**

| Placental Histology | **SP**  **n %** | | **MQ**  **n %** | | **p-value*** |
| --- | --- | --- | --- | --- | --- |
| ITT  Acute infection | 8 | 0.6 | 32 | 1.3 | 0.112 |
| Chronic infection | 40 | 3.2 | 58 | 2.3 |  |
| Past infection | 100 | 8.1 | 190 | 7.6 |  |
| Not infected | 1094 | 88.1 | 2222 | 88.8 |  |
| ATP  Acute infection | 7 | 0.6 | 26 | 1.3 |  |
| Chronic infection | 39 | 3.4 | 52 | 2.7 | 0.094 |
| Past infection | 94 | 8.1 | 135 | 6.9 |  |
| Not infected | 1014 | 87.9 | 1731 | 89.0 |  |

* Pearson Chi square
