# Supplementary material for: Intermittent Preventive Treatment of Malaria in Pregnancy with Mefloquine in HIV-Negative Women: A Multicentre Randomized Controlled Trial
Source: PLoS Med. 2014 Sep 23;11(9):e1001733. doi: 10.1371/journal.pmed.1001733 (PMC4172436; doi:10.1371/journal.pmed.1001733)
Supplement: Table S7 — Results showing the Number Needed to Treat (NNT) or Harm (NNH) by outcome. (DOCX) [file pmed.1001733.s009.docx]

**Table S7. Results showing the Number Needed to Treat (NNT) or Harm (NNH) by outcome**

|  | **SP**  **n/N %** | | **MQ**  **n/N %** | | **Risk**  **Difference** | **95% CI** | **NNH^1^ or NNT^2^** |
| --- | --- | --- | --- | --- | --- | --- | --- |
| Prevalence of LBW  ITT  ATP | 177/1398  128/1289 | 12.7  9.9 | 360/2778  221/2146 | 13.0  10.3 | 0.0030  0.0037 | -0.0185; 0.0244  -0.0171; 0.0245 | 335.5^1^  271.7^1^ |
| Maternal parasitemia at delivery  ITT  ATP | 63/1372  59/1255 | 4.6  4.7 | 88/2737  68/2100 | 3.2  3.2 | -0.0138  -0.0146 | -0.0267;-0.0009  -0.0286;-0.0007 | -72.6^2^  -68.3^2^ |
| Placental infection (Histology or smear)  ITT  ATP | 72/1281  70/1192 | 5.6  5.9 | 119/2568  103/1996 | 4.6  5.2 | -0.0099  -0.0071 | -0.0249; 0.0051  -0.0236;0.0094 | -101.4^2^  -140.4^2^ |
| Maternal anemia at delivery (Hb<11 g/dl)  ITT  ATP | 609/1380  543/1258 | 44.1  43.2 | 1110/2743  833/2104 | 40.5  39.6 | -0.0366  -0.0357 | -0.0686;-0.0046  -0.0702;-0.0013 | -27.3^2^  -28.0^2^ |

^1^NNH: Number needed to Harm. ^2^NNT: Number needed to Treat.
