# Supplementary material for: Intermittent Preventive Treatment of Malaria in Pregnancy with Mefloquine in HIV-Negative Women: A Multicentre Randomized Controlled Trial
Source: PLoS Med. 2014 Sep 23;11(9):e1001733. doi: 10.1371/journal.pmed.1001733 (PMC4172436; doi:10.1371/journal.pmed.1001733)
Supplement: Text S1 — MiPPAD study protocol. (PDF) [file pmed.1001733.s010.pdf]

# EVALUATION OF THE SAFETY AND EFFICACY OF MEFLOQUINE AS INTERMITTENT PREVENTIVE TREATMENT FOR MALARIA IN PREGNANCY

**EDCTP- IP.07.31080.002**

***Malaria in Pregnancy Preventive Alternative Drugs***
***MiPPAD***

**NCT 00811421**


Coordinator**: Clara Menéndez, MD, PhD**

**Barcelona Centre for International Health Research (CRESIB)**

Hospital Clínic
Universitat de Barcelona
C/Rosselló 132
08036 Barcelona
Spain


*Confidential*

| | |
|---|---|
| **Protocol No.:** | **Protocol EDCTP- IP.07.31080.002** |
| **Date:** | 15th July 2011 |
| **Version:** | Version 04 |
| **Previous versions** | Version 03, 1st October 2009 |
| **Title:** | Evaluation of the safety and efficacy of mefloquine Intermittent Preventive Treatment **for Malaria** in Pregnancy (IPTp). |
| **Countries :** | Benin, Gabon, Kenya,Tanzania, Mozambique |
| **Sites:** | Allada (Benin)<br>Lambaréné (Gabon)<br>Kisumu (Kenya)<br>*Dodoma* (Tanzania)<br>Manhiça (Mozambique) |
| **Study drug** | Mefloquine<br>Sulfadoxine-pyrimethamine<br>Cotrimoxazole |
| **Sponsor:** | Fundació Clínic per a la Recerca Biomèdica (FCRB) |
| **Clinical Trial registration #** | NCT 00811421 |
| **Co-ordinating Investigator:** | Clara Menéndez |
| **Address:** | Barcelona Centre for International Health Research<br>C/Rosselló 132, 4ª<br>08036 Barcelona- Spain |
| **Telephone:** | +34 93 2275400 |
| **Fax:** | +34 93 227 1850 |
| **Email:** | Menendez@clinic.ub.es |

## Signatures:

**Dr Clara Menéndez**
**Coordinator**

# Institutions involved in the study

## 1. African partners:

Achille Massougbodji, **Smaïla Ouedraogo**
Faculté des Sciences de la Santé (FSS), Université d'Abomey Calavi.
Cotonou, Benin

Ayola Akim Adegnika, Selidji Todagbe Agnandji, Ghyslain Mombo-Ngoma
Medical Research Unit (MRU), Albert Schweitzer Hospital
Lambaréné, Gabon

Peter Ouma, Mary Hamel
Kenya Medical Research Institute (KEMRI)/ Center for Global Health Research
Kisumu, Kenya

Hassan Mshinda, Abdunoor Mulokozi Kabanywanyi, *Mwaka Athmani*
Ifakara Health Institute (IHI)
*Dodoma*, Tanzania

Eusebio Macete, Esperança Sevene
Centro de Investigaçao em Saúde da Manhiça (CISM)
Manhiça, Mozambique

## 2. European partners

Michel Cot, Valérie Briand
Institut de Recherche pour le Développement (IRD)
Paris, France

Michael Ramharter, Peter G Kremsner
Institute of Tropical Medicine, University of Tübingen
Tübingen, Germany

Clara Menéndez, Raquel González, *John J. Aponte, Alfredo Mayor*
Centre de Recerca en Salut Internacional de Barcelona (CRESIB)
Hospital Clínic- Fundació Clínic per a la Recerca Biomèdica
Barcelona, Spain

## 3. Other collaborators

Vienna School of Clinical Research (VSCR), Austria

Meghna Desai, Laurence Slutsker
US Centers for Disease Control and Prevention

Malaria in Pregnancy (MiP) Consortium

# Table of Contents

LIST OF ABBREVIATIONS ........ 6
PROTOCOL SYNOPSIS ........ 7
SCHEMATIC OF STUDY DESIGN ........ 8
1 BACKGROUND ........ 9
2 JUSTIFICATION ........ 11
3 HYPOTHESES ........ 11
4 OBJECTIVES ........ 11
5 DESIGN AND METHODOLOGY ........ 12
*5.1 Trial comparing IPTp-SP versus IPTp-MQ in HIV non infected women receiving LLITNS ........ 12*
Study sites: ........ 12
Study population: ........ 12
Trial design: ........ 13
Primary endpoint: ........ 13
Secondary endpoints: ........ 13
Sample size ........ 13
Selection of the participants ........ 14
Procedures ........ 14
*5.2 Trial comparing IPTp-MQ versus IPTp- placebo in HIV infected women receiving CTX and LLITNs ........ 22*
Study sites ........ 22
Study population ........ 22
Trial design ........ 22
Primary endpoint ........ 22
Secondary endpoints ........ 22
Sample size ........ 23
Selection of the participants ........ 23
Procedures ........ 23
*5.3 Ancillary studies: ........ 32*
*5.4 Withdrawal criteria ........ 32*
*5.5 Protocol deviation/ exception/ violation ........ 32*
6 LABORATORY PROCEDURES ........ 33
*6.1 Parasitological and haematological determinations ........ 33*
*6.2 Histological analysis of the placenta ........ 33*
*6.3 Detection of HIV and quantitative determination of viral load ........ 33*
*6.4 Immunological determinations related to HIV status ........ 33*
7 SAFETY ........ 34
*7.1 Adverse Events ........ 34*
*7.2 Severity, relationship of event to study drug, and outcome ........ 35*
*7.3 Definition of Serious Adverse Event (SAE): ........ 36*
*7.4 Monitoring of AEs ........ 36*
*7.5 Tolerability/acceptability ........ 36*
*7.6 Data Safety Monitoring Board (DSMB) ........ 36*
8 DATA MANAGEMENT ........ 37
*8.1 Case Report Forms ........ 37*
*8.2 Quality assurance and quality control ........ 37*
*8.3 Site monitoring ........ 38*
*8.4 Data collection ........ 38*
*8.5 Database management ........ 39*
9 DATA ANALYSIS ........ 39
*9.1 Populations for analysis ........ 39*
*9.2 Trial Profile ........ 39*
*9.3 Patient demographics/other baseline characteristics ........ 40*
*9.4 Statistical models and methods of analysis: ........ 40*
*9.5 Safety ........ 41*
*9.6 Tolerability ........ 41*
10 INVESTIGATOR RESPONSIBILITY ........ 41
11 ETHICAL CONSIDERATIONS ........ 42
12 TIMEFRAME ........ 44
13 ADMINISTRATIVE PROCEDURES ........ 44

14 REFERENCES ........ 46
15 APPENDICES ........ 49
*Appendix 1. Additional Information on Study drugs* ........ *49*
*Appendix 2. Informed Consent form (Trial 1)* ........ *55*
*Appendix 3. National Guidelines for Malaria Treatment in Pregnant women* ........ *61*
*Appendix 4. Ballard Examination* ........ *62*
*Appendix 5. Psychomotor Development test* ........ *63*
*Appendix 6. National Guidelines of Malaria treatment for Infants* ........ *66*
*Appendix 7. Informed Consent Form (Trial 2)* ........ *67*
*Appendix 8. National polities for prevention of HIV MTCT* ........ *73*
*Appendix 9. SAEs reporting system* ........ *74*
*Appendix 10. Definitions* ........ *76*
*Appendix 11. World Medical Declaration of Helsinki* ........ *78*
*Appendix 12. Trials Flow* ........ *82*

# List of abbreviations

| | |
|---|---|
| ACTs | Artemisin–based combination therapies |
| AE | Adverse event |
| AIDS | Acquired Immunodeficiency Syndrome |
| AL | Artemether-Lumefantrine |
| AS | Artesunate |
| AZT | ZIdovudine |
| CDC | Centers for Disease Control and Prevention |
| CRF | Case Report Form |
| CRESIB | Barcelona Centre for International Health Research |
| CTX | Cotrimoxazole |
| DHA | Dihydroartemisin-Piperaquine |
| DSS | Demographic Surveillance System |
| EC | Ethics Committee |
| EDCTP | European and Developing Countries Trial Partnership |
| EPI | Expanded Program on Immunization |
| FCRB | Fundació Clínic per a la Recerca Biomèdica |
| Hb | Haemoglobin |
| HIV | Human Immunodeficiency Virus |
| IRB | Insitutional Review Board |
| ITNs | Insecticide Treated Nets |
| IPTp | Intermittent Preventive Treatment in pregnancy |
| LLITNs | Long Lasting Insecticide Treated Nets |
| MiP | Malaria in Pregnancy |
| MQ | Mefloquine |
| MTCT | Mother to child Transmission |
| MUAC | Middle Upper Arm Circumference |
| NVP | Nevirapine |
| PCR | Polymerase Chain Reaction |
| RPR | Rapid Plasma Reagin test (syphilis) |
| SAE | Severe Adverse Event |
| SOP | Standard Operating Procedures |
| SP | Sulfadoxine-pyrimethamine |
| 3TC | Lamivudine |
| WHO | World Health Organization |

# Protocol synopsis

The current recommendation by the World Health Organization (WHO) to prevent malaria infection in pregnancy in areas of stable malaria transmission relies on:

i) Prompt and effective case management of malaria illness
ii) The use of intermittent preventive treatment (IPTp) with at least 2 treatment doses of sulfadoxine-pyrimethamine (SP) and
iii) The use of insecticide treated nets (ITNs).

However, the spread of parasite resistance to SP, particularly in eastern Africa, and the significant overlap in some regions of malaria transmission and high prevalence of HIV infection, have raised concerns about the medium and long-term use of SP for IPTp.

HIV infection increases susceptibility to malaria and may reduce the efficacy of interventions. The evaluation of alternative antimalarials for IPTp is thus urgently needed also involving HIV infected women.

Of all the current available alternative antimalarial drugs, mefloquine (MQ) is the one that offers the most comparative advantages to SP.

A randomized multicenter trial will be conducted in 4 sites in Africa (Benin, Gabon, Tanzania and Mozambique) in order to compare the safety and efficacy of SP *versus* MQ as IPTp in the context of ITNs. In addition, **MQ tolerability will be also evaluated by comparing the administration of MQ as a single intake with its administration as split dose in two days.** In total **4716** pregnant women will be enrolled at the antenatal clinic (ANC) and will be followed until the infant is one year old.

Besides, in those countries were HIV prevalence in pregnant women is >10%, MQ- IPTp will be compared to Placebo-IPTp in HIV infected pregnant women receiving cotrimoxazole (CTX) prophylaxis. This trial will be double blinded and will be carried out in Kenya, Tanzania and Mozambique. It will involve 1070 pregnant women that will be followed until the infant is 2 months old.

# Schematic of Study Design

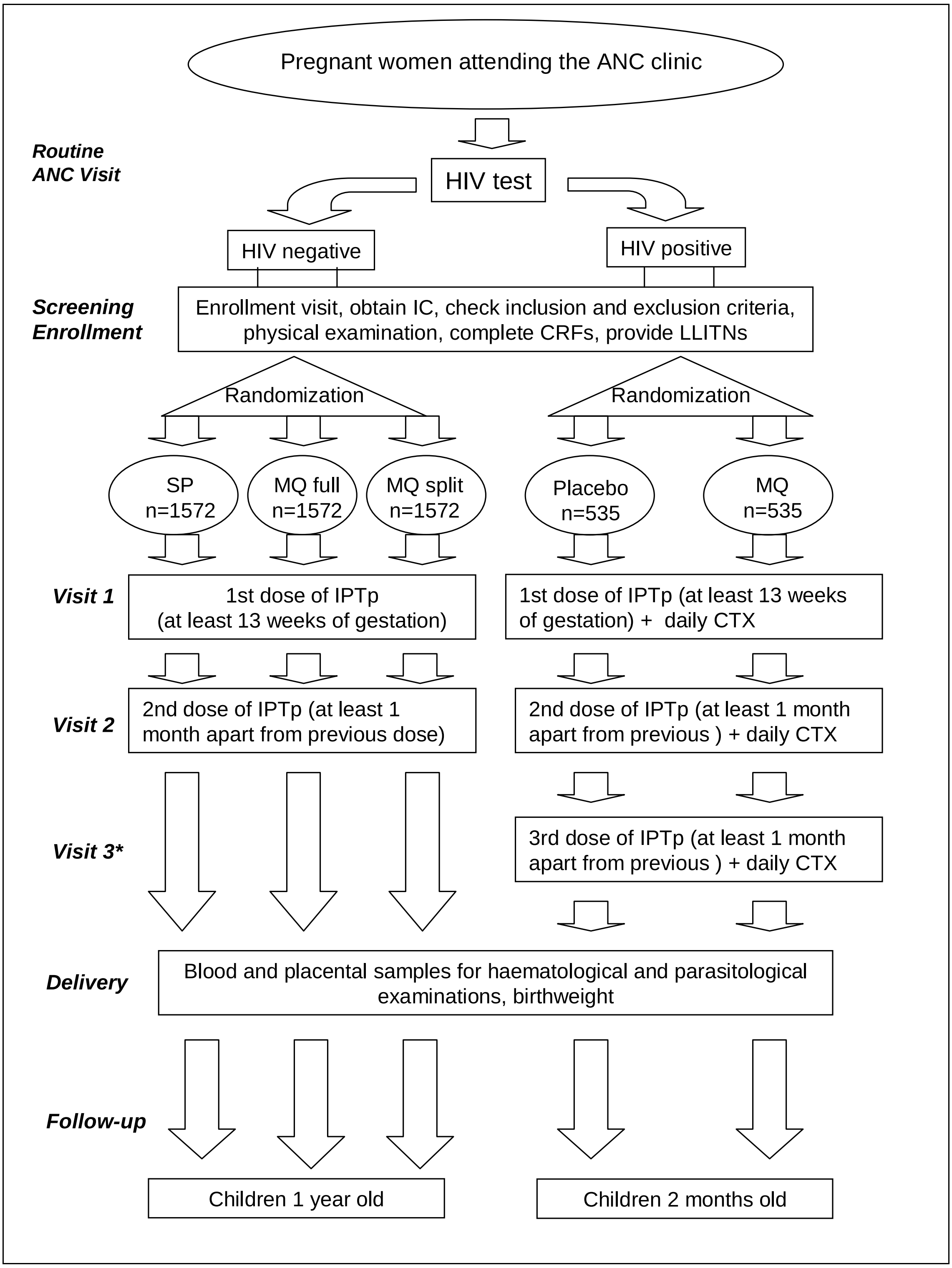


* *Visit 3 is only planned in HIV infected women.*

# 1 Background

Every year 50 million women become pregnant in areas where malaria is endemic, half of whom live in Africa[1]. In most of these areas malaria transmission is stable and *P.falciparum* predominates[2]. Primigravidae women and to a lesser extent secundigravidae have the highest risk for malaria infection[3,4]. These infections are associated with maternal anaemia, low birth weight and premature delivery[,5,6,7,8]. Recent studies suggest that even clinical episodes and maternal deaths due to malaria may be more common in sub-Saharan Africa than have been recognized[9,10,11]. Prevention of infection remains the best and most effective way to minimize negative health impacts for pregnant women and their infants.

For many years malaria prevention in pregnancy consisted of regular chemoprophylaxis with chloroquine, and more recently on the administration of intermittent preventive treatment (IPTp) with sulphadoxine- pyrimethamine (SP) and the use of Insecticide Treated Nets (ITNs)[1]. Both IPTp and ITNs have shown to be efficacious in reducing the harmful effects of malaria during pregnancy[12,13,14,15] and current recommendations for malaria prevention in African pregnant women rely on both IPTp and ITNs. Although an increasing number of African countries are adopting this recommendation, the coverage of these two strategies is still unacceptably low and far from the Abuja Declaration of 2000- in which regional leaders committed to achieving 60% coverage of pregnant women at risk for malaria with available control tools by 2005[1]. Thus, malaria continues to exert a huge toll on pregnant women and their newborns in endemic areas of sub-Saharan Africa.

The currently recommended regimen for IPTp is at least 2 treatment doses of SP given from the 2nd trimester onwards, given at least one month apart[1]. Despite its wide recommendation, several knowledge gaps remain. The spread of parasite resistance to SP, particularly in eastern Africa, and the significant overlap in some regions of malaria transmission and high HIV infection prevalence have raised concerns about the use of SP for IPTp. For instance, SP is not recommended to HIV-positive women receiving cotrimoxazole prophylaxis or antiretroviral drugs due to fear of an increased incidence of adverse drug reactions[15]. Thus, the evaluation of alternative antimalarials for IPTp is urgently needed.

Although the information regarding the effect of combining ITNs and IPTp is still very limited, recent studies suggest a synergistic effect on some parameters (anaemia, peripheral parasitaemia, and rate of clinical malaria) by combining both interventions[16,17]. The extended benefit provided to pregnant women using ITNs includes protecting infants after birth, suggesting that ITNs should be considered as an essential malaria intervention tool in stable transmission areas. This implies that in these areas preventive or case management studies in pregnant women should be carried out in the context of deployed ITNs as part of the study.

Many African pregnant women are infected with HIV, which increases susceptibility to malaria, reduces the efficacy of malaria interventions and complicates the use of antimalarials due to potential drug interactions. Current HIV/AIDS control guidelines recommend not giving IPTp with SP to pregnant HIV-positive women who are on cotrimoxazole (CTX) prophylaxis, for fear of increased risk of adverse events[15]. This recommendation is further supported by the idea that CTX is effective in preventing malaria in pregnant, HIV-positive women. However, the evidence of the antimalarial effect of CTX relies only on studies carried out in HIV-negative children and in HIV-positive non-pregnant adults who also received ITNs[18,19]. It is still unknown whether CTX is effective in preventing the adverse effects of malaria in pregnancy in HIV-positive women, whom interestingly have an increased risk for malaria infection and disease[20].

*Alternative antimalarial candidates to SP*
Several promising candidates are already available, some of which are, or have been used, for prophylaxis in pregnant women and have sufficient safety data to warrant their evaluation for IPTp. Mefloquine (MQ) is the most promising candidate. Of all the current alternative antimalarials for IPTp, MQ is the one that offers the most comparative advantages because:

a) it has a **long half- life** (median between 14 and 28 days at curative doses and between 12 and 17 days at prophylactic doses ) which increases its efficacy in IPTp.
b) it can be given as a **single dose**, which is very relevant for compliance.
c) **Resistance** is **rare** in Africa; in spite of the demonstration of plasmodial strains resistant to MQ[21,22] and of a clinical failure rate of 22% in a single study in Malawi[23], it proved to be highly efficacious to cure malaria in most parts of Africa. Recent studies indicate clinical efficacy rates by day 28 of 97.5% in Beninese children[24] and in Sudanese pregnant women[25] and of 99% in travellers returning from West Africa[26].
d) it has an **acceptable reprotoxicity** profile in animal studies
e) it is **recommended** for chemoprophylaxis for **pregnant** women travellers of all gestational ages by the WHO and the CDC, although limited information exists on its safety during the first trimester[27,28]. In 2001 inFrance, MQ received an official clearance (AMM) to be used at curative or prophylactic doses at any gestational age.

One concern could be the association of MQ with an increased risk of stillbirths found in a retrospective study among 208 Karen women who received MQ to treat malaria episodes[29]. However, this finding was not confirmed in a larger prospective trial of MQ prophylaxis in Malawian pregnant women[30] and remains unexplained. MQ is well tolerated at prophylactic doses (5 mg/kg/wk), although side effects (mainly dizziness and gastrointestinal symptoms), have been described when used for treatment. There is scarce information on the tolerability of MQ given in treatment doses (20-25 mg/kg) to pregnant, malaria asymptomatic women[31,32].

A trial comparing MQ with SP for IPTp **has recently been conducted** among pregnant women in Benin under the coordination of one of the partners of this project[33,34]. Although this trial slightly differed on some design aspects with the currently proposed multicenter project, **it has provided important information. The preliminary results from the study in Benin show that a therapeutic dose of MQ of 15 mg/kg is as efficacious as SP in preventing the effect of malaria in pregnancy in reducing brithweight. Moreover, MQ was found to be more efficacious than SP in preventing placental malaria infection. On the other hand, women that received MQ presented higher frequency of minor side effects than those who received SP: 28% vomiting and 26% dizziness, compared to 6% and 7% in the SP group respectively. Consequently, the tolerability of different dose regimens of MQ should be assessed before a potential decision in changing the policy of IPTp is made.**

**MQ tolerability could be improved by splitting the total dose of MQ in two days of administration[35]. Evidence from studies in patients with acute malaria from South East Asia shows that MQ at 25 mg/kg split over 3 days is better tolerated than 25 mg/kg split over 2 days, which in turn is better tolerated than a single dose of 25 mg/kg[32]. Interestingly, splitting the MQ dose increases the area under the curve (AUC) by about 50% for monotherapy, probably resulting from better oral bioavailability in the patients[36]. It is unknown whether these same results would apply when MQ is administered to asymptomatic infected or not infected pregnant women.**

**In the proposed trial the efficacy of the current regimen of IPTp with SP will be compared with that of MQ, which will be administered at the recommended treatment dose of 15 mg/kg. In order to provide information on the best regimen of MQ as IPTp, MQ tolerability will be assessed by comparing a single treatment course (full dose) with a split dose over two days.**

Currently, the WHO recommends treating all pregnant women with a 3-dose regimen of IPTp with SP in settings where HIV prevalence among pregnant women is greater than 10%. Thus, in this study 3 doses of IPTp (MQ or Placebo) will be administered to HIV infected women in accordance with international health guidelines.

# 2 Justification

Current prevention tools such as use of ITNs and IPTp with SP are available but face a number of important limitations. With the increase in resistance to SP in many African contexts, the evaluation of alternative antimalarials for IPTp is urgently needed.

The safety profile of any drug to be used for IPTp needs to be established because it will be used in all pregnant women, regardless of the presence of malaria infection. The drug needs to be well tolerated and easy to use to maximise compliance, and affordable. Although the precise mode of action of IPTp is not fully understood, the duration of post treatment prophylaxis is likely to be an important determinant of efficacy requiring drugs with long half-lives. These issues must be further examined and understood before policy recommendations can be made.

Therefore, clinical trials to test IPTp drug alternatives will make a significant contribution to rational policy formation for malaria prevention in this vulnerable group.

# 3 Hypotheses

1. In the view of increasing resistance of *P falciparum* to sulfadoxine-pyrimethamine (SP) and in the presence of ITNs, mefloquine (MQ) is more efficacious than SP for IPTp in the prevention of malaria in pregnancy
2. MQ-IPTp is as safe as SP-IPTp
3. **MQ given as a split dose is better tolerated than administered as full dose and has equal efficacy as IPTp**
4. In HIV infected pregnant women, the addition of MQ-IPTp to cotrimoxazole (CTX) prophylaxis is <u>more efficacious **than**</u> cotrimoxazole alone in the prevention of malaria in pregnancy
5. HIV-infected pregnant women receiving CTX and IPTp-MQ present a better immunologic profile (lower HIV viral load and higher CD4 counts) than those receiving CTX and IPTp-placebo at delivery

# 4 Objectives

*Overall objective*:
To evaluate the safety and efficacy of alternative antimalarial drugs to SP for IPTp in the context of ITN use in different malaria endemic settings in Africa.

*Primary objectives:*
- To compare the safety, tolerability and efficacy of MQ to SP as IPTp for the prevention of malaria in pregnancy for the mother and her infant.
- To determine the safety and efficacy of IPTp with mefloquine among HIV infected women receiving CTX prophylaxis for opportunistic infections.

*Secondary objectives:*
- **To compare MQ tolerability given as full dose with a split dose administered over 2 days**
- To evaluate the efficacy of CTX in the prevention of malaria infection in pregnant women.
- To compare immune status of HIV infected women receiving CTX + IPTp-MQ to those receiving CTX + IPTp-placebo.
- To assess the safety of study drugs in the development of infants.

# 5 Design and methodology

In order to comply with the objectives of the project, 2 trials will be conducted. See Figure 1.

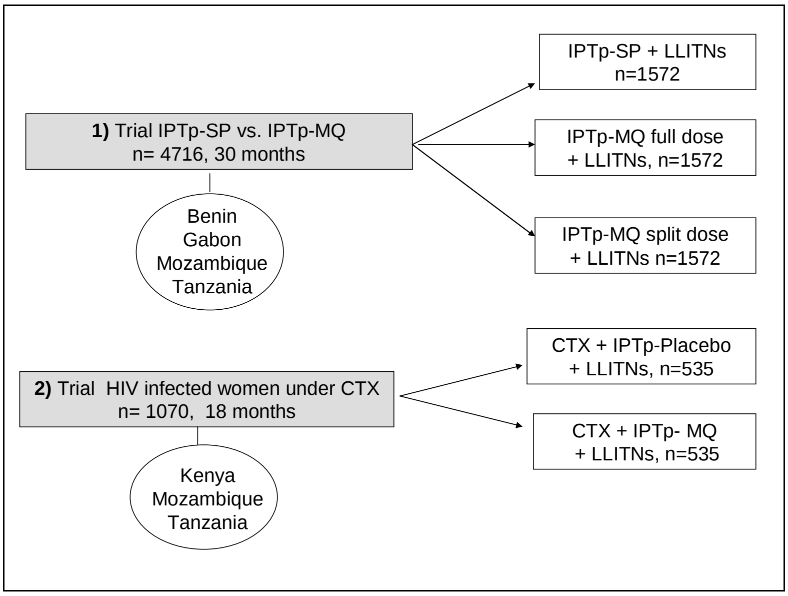


**Figure 1. Diagram of the studies**

## 5.1 Trial comparing IPTp-SP *versus* IPTp-MQ in HIV non infected women receiving LLITNS

**Study sites:**
This trial will be conducted in **four sites** in Benin, Gabon, Tanzania and Mozambique. It will thus involve regions from Western, Eastern, Central and Southern **sub-Saharan Africa** where malaria transmission is stable but displays distinctly varying characteristics according to the site. *P falciparum* accounts for more than 95% of malaria infections in all four sites, and the level of SP resistance varies from moderate (12.5-25%, treatment failure at day 14) to intermediate (25-49%)[24]
.

**Table 1: Malaria epidemiology in the study countries**

| Country | Site | Malaria Transmission | High season | EIR | *P.falciparum* infection |
|---|---|---|---|---|---|
| Benin | Allada | Hyperendemic | Apr-Jul<br>Sep-Nov | 51-100 | > 90% |
| Gabon | Lambaréné | Hyperendemic | Oct-May | 21-50 | >90% |
| Mozambique | Manhiça | Mesoendemic | Sep-Mar | 21-50 | >90% |
| Tanzania | Dodoma | Mesoendemic | Jun-Aug | 21-50 | >90% |

EIR: Entomological Inoculation Rate

**Study population:**
The participants of the study will be HIV-negative pregnant women attending the ANC services in the study areas.

**Trial design:**
This is a randomized open-label **superiority** 3 arms trial to compare 2-dose MQ versus 2-dose SP for IPTp in the prevention of the adverse effects of malaria during pregnancy **and to compare MQ tolerability of 2 different MQ administration regimens. The three arms of the study will be:**

1. **IPTp with SP**
2. **IPTp with MQ given as full dose**
3. **IPTp with MQ given as an split dose**

**Primary endpoint:**
Prevalence of low birth weight (LBW) babies (<2500 g).

**Secondary endpoints:**

1. Peripheral maternal parasitaemia at delivery (microscopic and submicroscopic*)
2. Prevalence of *P falciparum* parasitaemia in cord blood (microscopic and submicroscopic*)
3. Prevalence of placental *P. falciparum* infection (histology and blood smear)
4. Mean maternal haemoglobin rate (in g/dL)
5. Prevalence of maternal anaemia at delivery (< 11g Hb/dL)
6. Prevalence of severe maternal anaemia at delivery (< 7g Hb/dL)
7. Prevalence of *neonatal* anemia (*Hb<12.5 g/dL in case of cord blood, and Hb <13 g/dL in case of peripheral blood* )
8. Mean birth weight (in grams)
9. Prevalence of prematurity
10. **Number of stillbirths**
11. **Number of miscarriages**
12. **Frequency** of congenital malformations.
13. Mean gestational age *at birth (assessed by Ballard score in the newborn)*
14. **Incidence of vomiting**
15. **Incidence of dizziness**
16. **Frequency** of drug adverse reactions
17. Incidence of clinical malaria during pregnancy
18. Incidence of overall admissions/outpatient attendances
19. Neonatal and infant mortality rate
20. Incidence of malaria in the first year of life
21. *Peripheral maternal parasitaemia 1 month after end of pregnancy (microscopic and submicroscopic*)*

**Only in a subsample of participants*

**Sample size**
***Assumptions for Primary Endpoint: SP vs MQ***
Assuming a prevalence of LBW (primary outcome) of 12% in the context of IPTp with SP and ITN use, **1257** women will be needed **in the SP group and 2514 in the MQ groups (1257 participants in each MQ group) to detect 25% lower prevalence of LBW** in the MQ group (9%) with a 80% power and a 0,05 significance level using a two-sided Chi-square test. **Efficacy (defined by the proportion of LBW at delivery) of MQ is assumed to be the same in both groups receiving the drug (either as a split as a full dose).**

***Assumptions for MQ tolerability evaluation: MQ full dose vs MQ split dose***
**Assuming a proportion of vomiting of 30% in the MQ full dose group, a sample size of 1257 women in the MQ single dose group and 1257 women in the MQ split dose group will achieve 80% power to detect a 6% reduction in vomiting between the two groups at a significance level of 0,05.**

To allow for 20% losses to follow-up, **1572 pregnant women will be recruited per study arm among the 4 sites (1179 participants/ site). The total sample size to be recruited is 4716 pregnant women.**

**Selection of the participants**

*Inclusion criteria*
All pregnant women attending the ANC services at the 4 sites will be included into the study and will be asked to sign the informed consent if they meet the following inclusion criteria:

- Permanent resident in the area
- Gestational age at the first antenatal visit ≤ 28 weeks
- Agreement to deliver in the study site's maternity(ies) wards

*Exclusion criteria:*
Women with any of the following criteria will not be admitted to the study:

- Residence outside the study area or planning to move out in the following 18 months from enrollment
- Gestational age at the first antenatal visit > 28 weeks of pregnancy
- Known history of allergy to sulfa drugs or mefloquine
- Known history of severe renal, hepatic, psychiatric or neurological disease
- MQ or halofantrine treatment in the preceding 4 weeks
- HIV infection
- Participating in other **intervention** studies

**Procedures**

***Visit 1: Screening visit/ Enrollment/ Administration of 1st IPTp dose:***

***a) Screening***
All pregnant women attending the ANC services *for the 1st time and/or who have not received IPTp during their current pregnancy will* be screened for participation in the trial. In case a woman fails to be enrolled (screening failures), the reason for not being enrolled will be recorded in the Screening Log. Women of all parities will be included in order to assess the usefulness of IPTp administration to women at higher risk (primiparas) and at lower risk (secundi- & multi-pars) of MiP.

***b) HIV counseling/testing***
HIV status will be assessed after voluntary counseling and testing according with the national guidelines for HIV/AIDS prevention in place. Those women found to be HIV positive will not be eligible for the trial and will be offered administration of ARVs for the prevention of mother to child transmission of HIV (depending on the national preventive guidelines).

In Tanzania and Mozambique, HIV-positive women will be invited to participate in the trial comparing IPT-MQ *versus* IPT-placebo in HIV infected women receiving CTX (please see section 5.2).

***c) Informed Consent***
A signed enrollment informed consent (or thumb-printed whenever the woman is illiterate) will be obtained before any tests or evaluations related to the study eligibility are carried out. Depending on national IRB local policies, if the woman is under 18 years of age, she will sign the assent form and a legal guardian of the woman will sign the informed consent. The informed consent will cover the woman and the newborn infant (Appendix 2).

***d) Randomization***
After the study details are explained and informed consent is signed, pregnant women will be given a study number and randomized to either SP, MQ **full dose or MQ split dose group.** Each participant will be uniquely identified in the study by a combination of her site code and subject number.

The site code will be assigned centrally by the coordinator partner (CRESIB) and the patient number by the investigator. At each site consecutive study numbers will be assigned to the participants. An identification card containing the individual study number and basic demographic information will be given to the participant in order to facilitate identification at all study contacts.

Study numbers will be allocated randomly to one of the **three** treatments in blocks of varying size. A list of study numbers and allocated treatments will be produced. This list will be handed over to and kept by the study DSMB until the code is open for the analysis. Opaque bags or blisters with the study treatments will be labeled with the patients unique study number according to the randomization list. Both the randomization list and the labeling of the study medication will be done by the CRESIB.

***e) Demographic data and medical history***
The woman's demographic and obstetric data (including the date of last menses) plus the history of any allergies to the study drugs will be recorded in a specific designed standardized case report form (CRF).

***f) Physical and clinical examination***
The physical examination of the women will include the following assessments: weight, height, gestational age by bimanual palpation and MUAC.

If the gestational age at the time of recruitment coincides with the fundus being palpable (at least 13 weeks of gestation), the woman will be given the first dose of IPTp. Otherwise, she will be invited to come back coinciding with the next antenatal appointment to receive the intervention.

If women **passively report** to be sick during the ANC visit, they will be referred to the clinical services as **per routine system in place**. In case of malaria parasitaemia or anaemia, they will be treated following national guidelines (Appendix 3) and specific study SOPs.

***g) Laboratory tests***
A sample of capillary blood (1,5 ml) will be collected for confirmatory HIV test, blood hemoglobin determination and RPR test (as per routine antenatal care). The test results will be recorded on the recruitment standardized questionnaire.

In addition, 5 ml of blood may be taken in a sub-sample of study participants for ancillary studies (please refer to section 5.3 for more information on ancillary studies).

***h) Administration of IPTp***
Following physical examination, recruited women with more than 13 weeks of gestational age will receive the 1st dose of IPTp (either SP or MQ) under supervision.

The women allocated to the SP group will receive 3 tablets of the fixed combination therapy containing 500 mg of sulfadoxine and 25 mg of pyrimetamine. Whereas study participants allocated to the MQ groups will receive 15mg/kg of MQ as IPtp. Each MQ tablet contains 250 mg of mefloquine base. The number of MQ tablets to be given to the woman will be administered according to the maternal weight at the time *of 1st IPTp administration* (i.e. a woman weighting 60 kg will receive 3 MQ tablets and a half).*The number of MQ tablets administered to women*

*weighing more than 100 kg shall not exceed the maximum dosage limit of 1, 5 g which corresponds* ***to 6 tablets.***
**Women allocated to MQ full dose group will receive the full IPTp dose on one time. Whereas those allocated to the MQ split dose group will be administered MQ tablets in 2 days. The second half of the MQ dose will be administered at home** *(or ANC)* **by study personnel, who will be observing the woman for 30 minutes, to record immediate tolerability.**

**All** women will be observed for **60** minutes after administration of the IPT. Those women vomiting within the first 30 minutes will be given a second full IPT dose; those vomiting from 30-60 minutes will be given an additional half dose.

The tolerability of the study drugs will also be assessed 2 days later after intake by home visits of study personnel. Any medical sign or symptom will be then recorded.

The second dose of SP/MQ will be given coinciding with the next ANC **visit at least one month apart from the previous dose.**

***i) Concomitant medications***
In some study sites, as per national guidelines women may receive Mebendazole (an antiparasitic drug given at prophylactic doses) and ferrous sulphate for anemia prevention, which dose depends on Hb values. *From the 1st ANC visit, all study women should receive ferrous sulphate-folic acid supplements for prevention of anemia in pregnancy as for national guidelines. It should be ensured that the dose of folic acid given* ***is not greater than 1,5 mg/day*** *to avoid interference with the antifolate effect of SP and facilitate malaria parasitemia.* Any concomitant treatment (including antibiotics or other antimalarial drugs) will be recorded in the study participant CRF.

***j) Long Lasting Insecticide Treated Nets***
Regardless of gestational age at time of recruitment, all women will receive a long lasting insecticide treated net (LLITN) at the time of enrollment and details about its use will be explained.

***k) Follow-up***
Women will be given an appointment to attend the second ANC visit at least 1 month after the first one. They will also be asked to come to the study facilities in case of any illness. **Women will be visited at home the following day of recruitment to confirm residence status, assess drug tolerability and the correct use of the net.**

## ***Visit 2: Administration of 2nd dose of IPTp***

***a) Physical and Clinical Examination***
**Maternal weight will be measured and the gestational age assessed. Only if women passively report to be sick during the ANC visit, they will be referred to the clinical services as per routine system in place. In case of malaria parasitaemia or anaemia, they will be treated following national guidelines (Appendix 3) and the specific study SOPs.**

Five milliliters of blood may be taken in a sub-sample of study participants for ancillary studies (please refer to section 5.3 for more information on ancillary studies).

***b) Administration of IPTp***
Following physical examination, women will receive the 2nd dose of IPTp under supervision.The women allocated to the SP group will receive 3 tablets of the fixed combination therapy containing 500 mg of sulfadoxine and 25 mg of pyrimetamine.

The women allocated to the MQ groups will receive the same total amount of MQ given at the 1st ANC visit (according to the maternal weight at the time of recruitment considering 15mg/kg). For

example, a woman weighting 60 kg at enrollment will receive 3 MQ tablets and a half as second IPTp dose.

**Women allocated to MQ full dose group will receive the full IPTp dose on one time. Whereas those allocated to the MQ split dose group will be administered MQ tablets in 2 days. The second half of the MQ dose will be administered at home *(or ANC)* by study personnel, who will be observing the woman for 30 minutes, to record immediate tolerability.**

**All** women will be observed for **60** minutes after administration of the IPT. Those women vomiting within the first 30 minutes will be given a second full IPT dose; those vomiting from 30-60 minutes will be given an additional half dose.

In addition the tolerability of the study drugs will also be assessed 1 day after intake by home visits of study personnel. Any medical sign or symptom will be then recorded.

***c) Medical History***

Any concomitant treatment or history of treatment since the last visit will be recorded in the study CRF. In addition, information on adverse events related to study drugs will be collected.

***d) Follow up: household visits***

**Women will be visited at home after the 2nd ANC visit to assess drugs tolerability and the correct use of the net. In addition, a household visit will be scheduled 2 weeks before the estimated delivery date in order to remind the women to deliver at the study health facilities.**

### *Visit 3: Delivery/End of pregnancy/ Newborn assessments*

***a) Maternal Clinical Examination and assessments***

At the time of delivery or miscarriage, study women will be identified and the outcome of pregnancy recorded into the CRF.

**Blood pressure will be measured** and 5 milliliters (ml) of venous blood will be collected for haematological determinations and parasitological examination (including PCR filter paper) and for ancillary studies (the latest only on a sub-sample of study participants (please refer to section 5.3 for more information on ancillary studies).

***b) Newborn assessments***

A 5-8 ml blood sample for haematological determinations and parasitological examination (including PCR filter paper) will be collected from the umbilical vein once the baby's umbilical cord has been cut and clamped.

The newborn will be physically examined, weighed and measured and his/her gestational age assessed by the Ballard's method (Appendix 4). Date of birth, sex of the infant, axillary temperature and any observed congenital abnormalities will be recorded into a specific designed questionnaire.

***c) Placental samples***

A placental biopsy will be taken and placed in 10% neutral buffered formalin to prevent clotting and kept in a cool box until its transport to the site laboratory.

SOPs will be written to detail the procedures for placental samples processing and storage. Placenta samples will be processed at the site laboratory (please refer to section 6 for more details).

***d) Follow up***
Newborns will be given a study number independent from the mother's study number in order to be uniquely identified. Both mother and child study numbers will be recorded in the CRF. The newborn study number will be a combination of the site code and participant number.

The site code will be assigned by CRESIB and the patient number by the investigator. At each site the first newborn will be assigned a participant number, and consecutive numbers will be assigned to subsequent newborn.

The mother will be asked to come back to the study health facilities after 1 month of delivery, coinciding with the 1$^{st}$ visit of the child to the EPI. Whenever possible, a photo picture of the mother with the baby will be taken and attached to the identification card in order to facilitate identification at all study contacts.

***e) Home deliveries***
Women who deliver at home will be visited within one week of delivery and details of the delivery outcome will be recorded. **Newborn's weights captured 1 to 7 days after birth will be "adjusted" to estimate birth weight using a statistical factor used in previous studies conducted among African newborns**[37] Women will be invited to visit the study health facilities with their babies. At this visit a capillary blood sample (heel prick) will be taken, a thick blood smear and filter paper collected from both mother and infant and the weight of the baby measured.

***f) Multiple births***
In case the outcome of the pregnancy is multiple births, the first born infant will be assigned the first available consecutive study number. His/her sibling(s) will receive the following consecutive number. Newborns will be followed according to the described procedures of the study until they are 1 year old.

***g) Miscarriage and other pregnancy outcomes***
If the outcome of the pregnancy is a miscarriage or stillbirth, women will be visited at the study facilities and haematological and parasitological tests will be performed.

***Visit 4: One month after deliver***

**Study participants will be invited to attend the study health facility one month after delivery coinciding with the first scheduled EPI visit of the infant. Women will be clinically examined: axillary temperature will be measured and a capillary blood sample collected for haematological (Hb) determination and parasitological (thick blood smear and filter paper) examination. In case of malaria parasitaemia or anaemia, they will be treated following national guidelines.**

***Unscheduled visits:***

Study participants reporting sick at the health facilities will be seen by study personnel. Every unscheduled visit of the woman from enrollment until 2 months after delivery will be recorded in the CRF.

***a) Physical and clinical examination***
Gestational age, **blood pressure** and axillary temperature will be measured. Patients with fever (≥37,5ºC) or history of fever in the last 24 hours, **headache or arthromyalgias** or who appear pale[11] will have a thick and thin blood film done and an Hb test performed. In case of malaria parasitaemia or anaemia, they will be treated following national guidelines (Appendix 3).

***b) Other medications***

Any concomitant treatment (including antibiotics or other antimalarial drugs) or history of treatment since the last visit will be recorded in the study CRF. In addition, information on adverse events related to study drugs will be collected.

***Outline of the maternal visits***

From enrollment until delivery, pregnant women will be involved in study procedures according to the following schedule.

**Table 2. Maternal visits and procedures schedule**

| **Study procedure** | **Visit 1 Enrollment** | **Visit 2** | **Visit 3 Delivery** | **Visit 4 1 month** | **Unscheduled visits** | **Household visits** |
|---|---|---|---|---|---|---|
| Inclusion/ Exclusion criteria check | X | | | | | |
| Written informed consent | X | | | | | |
| Demographics/ Medical history | X | | | | X | |
| Record of concomitant medication / Adverse events | X | X | X | **X** | X | |
| Physical examination/clinical | X | | | | X | |
| Gestational age | X | X | X | | X | |
| Temperature | | | | **X** | X | |
| **Blood pressure** | | | X | | X | |
| Weight | X | X | | | X | |
| Height | X | | | | | |
| MUAC | X | | | | | |
| IPTp administration | X | X | | | | |
| RPR test | X | | | | | |
| HIV test | X | | | | | |
| Blood smear | * | * | X | **X** | * | |
| Haemoglobin test | X | | X | **X** | | |
| Peripheral venous blood (mother) | | | X | | | |
| Cord blood sample | | | X | | | |
| Placental biopsy | | | X | | | |
| Placental impression smears | | | X | | | |
| **Drug tolerability assessment** | X | X | | | | X |
| LLITNs | X | † | † | † | † | † |
| Ancillary studies | ‡ | ‡ | ‡ | | | |

* Only in women passively reporting sick AND presenting with malaria related signs/symptoms (fever (≥37,5º C) or having history of fever in the past 24 hours, arthromyalgias or headache), as per national management guidelines † Assessment of compliance with LLITNs use ‡ Blood sample may be taken in a sub-sample of study participants for ancillary studies (please refer to section 5.3 for more information on ancillary studies). *Filter paper will be collected at unscheduled visits (fingerprick), at delivery (from venous blood, cord blood and placental blood) and at the scheduled visit 1 month after delivery (fringerprick).*
*The allowed to timeframe for conducting the scheduled visits is shown in the trial flow (refer to Appendix 12).*

## *Follow up of study children*

Children born from study participants will be followed up until 12 months of age. Mothers will be asked to bring their child to the study health facilities at month 1 (or coinciding with the first EPI visit), **9 and 12** after birth.

***a) Site visits assessments****:*
At month 1 (or coinciding with 1st EPI visit), 9 and 12 after birth, study children will be physically examined by study personnel. The psychomotor and neurological development of the infant will be assessed following a simplified protocol (Appendix 5). Weight, height and axillary temperature will be measured and recorded.

A capillary blood sample will be taken from infants with fever (axillary temperature ≥37,5ºC) or history of fever in the last 24 hours, or appearing pale, for malaria parasitemia examination and haematological determination. In case of malaria parasitaemia or anaemia, they will be treated following national guidelines (Appendix 6).

In addition, 5 ml of blood may be taken only in a sub-sample of study participants for ancillary studies (please refer to section 5.3 for more information on ancillary studies).

***b) Household visits***
Study participants will be visited at home **in case they do not attend the scheduled visits at 9th and 12th month after birth**. Compliance with the use of LLITNs the previous night will also be recorded.

**c) *Unscheduled visit***
Throughout the 1st year of follow-up, the study infants reporting sick at the health facilities will be seen by study personnel. They will be physically examined. Weight, height, and axillary temperature will be measured and recorded.

Patients with fever (axillary temperature ≥37,5ºC) or history of fever in the last 24 hours or who appear pale will have a thick and thin blood film done. Blood hemoglobin will also be determined. In case of malaria parasitaemia or anaemia, they will be treated following national guidelines (Appendix 6)

## ***Outline of the infants visits***

From delivery until month 12 of age, study infants will be involved in study procedures according to the following schedule.

**Table 3. Infants visits and procedures schedule**

| Age | Birth | 1m§ | 9 m* | 12 m* | Unscheduled visits |
|---|---|---|---|---|---|
| Medical history | X | X | X | | X |
| Physical examination | X | X | X | X | X |
| Psychomotor development assessment | X | X | X | X | |
| MUAC | | X | X | X | |
| Weight | X | X | X | X | X |
| Height | X | X | X | X | X |
| Temperature | X | X | X | X | X |
| Blood smear | X | † | † | † | † |
| Haemoglobin test | X | † | † | † | † |
| Ancillary studies | | ‡ | ‡ | ‡ | |

m: month * Household visits in case they do not attend the scheduled visits at study health facilities; † Only if fever (≥37,5º C) or history of fever in the past 24 hours or signs suggestive of malaria ‡Blood sample may be taken in a sub-sample of study participants for ancillary studies (please refer to section 5.3 for more information on ancillary studies) § First visit will be scheduled 1 month after birth or coinciding with first EPI visit.

*The allowed to timeframe for conducting the scheduled visits is shown in the trial flow (refer to Appendix 12).*

Study infants that will not attend the scheduled site visits will be visited at home by study personnel. Demographic events such as migrations and deaths will be documented through the DSS (whenever in place in the study site). As the minimum, all the study mothers and her children will be visited at home 12 months after delivery for final assessments of status.

### 5.2 Trial comparing IPTp-MQ versus IPTp- placebo in HIV infected women receiving CTX and LLITNs

**Study sites**

This trial will be conducted in **3 sites** from south eastern sub-Saharan Africa (Kenya, Mozambique and Tanzania), where HIV prevalence in pregnant women ranges from 10 to 30%[38].

**Table 4: Malaria and HIV epidemiology in the study countries**

| Country/ Site | Malaria Transmission | High season | EIR | *P.falciparum* infection | HIV Prevalence* |
|---|---|---|---|---|---|
| Kenya / Kisumu | Holoendemic | May-Jul | 21-50 | >90% | *25%* |
| Mozambique/ Manhiça | Mesoendemic | Sep-Mar | 21-50 | >90% | *29%* |
| Tanzania / *Dodoma* | *Mesoendemic* | Jun-Aug | *21-50* | >90% | ***3.3%*** |

* Estimated HIV prevalence in pregnant women at the study sites *in 2011*.
EIR: Entomological Inoculation Rate

**Study population**

The participants of the study will be HIV positive pregnant women attending the ANC services in the study areas.

**Trial design**

This is a randomized double-blind **superiority** clinical trial **to compare the efficacy of MQ as I**PTp with placebo-IPTp in HIV-infected pregnant women receiving CTX prophylaxis.

**Primary endpoint**

Peripheral *maternal* parasitaemia at delivery (*microscopic and submicroscopic*)

**Secondary endpoints**

1. **Prevalence of *P falciparum* parasitaemia in cord blood (microscopic and submicroscopic*)**
2. Prevalence of placental *P. falciparum* infection (histology and blood smear)
3. **Mean maternal haemoglobin rate (in g/dL)**
4. *Prevalence of* maternal anaemia at delivery *(<11 g/dL*)
5. *Prevalence of severe maternal anaemia at delivery (<7 g/dL)*
6. Mean of CD4 counts and viral load at delivery
7. Prevalence of neonatal anemia (*Hb<12,5 g/dL in case of cord blood, and Hb<13g/dLin case of peripheral blood)*
8. **Mean birth weight (in grams)**
9. Prevalence of low birth weight babies (<2500 g)
10. Prevalence of prematurity
11. **Number of stillbirths**
12. *Number of miscarriages*
13. **Frequency of congenital malformations**
14. Mean gestational age *at birth (assessed by Ballard Score in the newborn*)
15. Incidence of vomiting
16. Incidence of dizziness
17. **Frequency of drug adverse reactions**
18. Incidence of clinical malaria during pregnancy
19. **Incidence of overall admissions/outpatient attendances**

20. Neonatal mortality
21. Perinatal mortality
22. *Peripheral maternal parasitemia 1 month after end of pregnancy (microscopic and submicroscopic*)*

**Only in a subsample of participants*

**Sample size**
Assuming a prevalence of peripheral parasitaemia at delivery of 15% with CTX prophylaxis, it is estimated that 453 women per arm will be required **to detect a decrease of 6,2% or more in the prevalence of peripheral parasitemia in the CTX+IPTp-MQ group (prevalence of 8,7%), with 80% power and 0,05% significance level using a two-sided Chi-square test.**

To allow for 15% losses to follow-up, **535** HIV infected pregnant women will be recruited **per study arm**.

**Selection of the participants**

*Inclusion criteria*:
All pregnant women attending the ANC services at the 3 sites will be **included into the study and will be asked to sign the informed consent** if they meet the following inclusion criteria:

- Permanent resident in the area
- Gestational age at the first antenatal visit ≤ 28 weeks
- HIV seropositive (after voluntary counseling and testing)
- Agreement to deliver in the study site's maternity(ies) wards

*Exclusion criteria:*
Women with any of the following criteria will not be admitted to the study:

- Residence outside the study area or planning to move out in the following 10 months from enrollment
- Gestational age at the first antenatal visit > 28 weeks of pregnancy
- Known history of allergy to CTX or MQ
- Known history of severe renal, hepatic, psychiatric or neurological disease
- MQ or halofantrine treatment in the preceding 4 weeks
- **Participating in other intervention studies**

**Procedures**

**Visit 1: Screening visit/ Enrollment/ Administration of 1st IPTp dose:**

***a) Screening***
All pregnant women attending the ANC services *for the 1st time and/or who have not received IPTp during their current pregnancy* will be screened for participation in the trial. In case a woman fails to be enrolled (screening failures), the reason for not being enrolled will be recorded in the Screening Log. Women of all parities will be included in order to assess the usefulness of IPTp administration to women at higher risk (primiparas) and at lower risk (secundi- & multi-pars) of MiP.

***b) HIV counseling/testing***

HIV status will be assessed after voluntary counseling and testing according to the national guidelines for HIV/AIDS prevention in place. The women will be initially tested with a HIV rapid test and positive results will be confirmed with a second rapid test as recommended. Only those women found to be HIV positive will be eligible for the trial. Apart from being invited to participate in the study, women will also be offered the administration of ARV for the prevention of mother to child transmission of HIV (depending on the national preventive guidelines).

***c) Informed Consent***

A signed enrollment informed consent (or thumb-printed whenever the woman is illiterate) will be obtained before any tests or evaluations related to the study eligibility are carried out. Depending on national IRB local policies, if the woman is under 18 years of age, she will sign the assent form and a legal guardian of the woman will sign the informed consent. The informed consent will cover the woman and the new born infant (Appendix 7).

***d) Randomization***

After the study details are explained and informed consent is signed, pregnant women will be given a study number and randomized to either CTX + IPTp-placebo or CTX + IPTp-MQ. Each participant will be uniquely identified in the study by a combination of her site code and participant number.

The site code will be assigned by CRESIB and the patient number by the investigator. A randomization list of blocks of varying size and stratified by study site will be provided by the Biostatistics Unit of the CRESIB.

At each site the first patient will be assigned a patient number, and consecutive numbers will be assigned to subsequent patients. An identification card containing the individual study number and basic demographic information will be given to the participant in order to facilitate identification at all study contacts.

Each subject number will be related to a treatment number which assigns them to one of the IPT arms. The investigator will enter the randomization number on the CRF.

***e) Demographic data and medical history***

The woman's demographic and obstetric data (including the date of last menses) plus the history of any allergies to the study drugs will be recorded in a specific CRF.

***f) Physical and clinical examination***

The physical examination of the woman will include the following assessments: weight, height, gestational age by bimanual palpation and MUAC.

If the gestational age at the time of recruitment coincides with the fundus being palpable (at least 13 weeks of gestation), the woman will be given the first dose of IPT. Otherwise, she will be invited to come back coinciding with the next antenatal appointment to receive the intervention.

**If women passively report to be sick during the ANC visit, they will be referred to the clinical services as per routine system in place. In case of malaria parasitaemia or anaemia, they will be treated following national guidelines (Appendix 3) and the specific study SOPs.**

***g) Laboratory tests***

At enrollment, the following blood tests will be performed (and its results recorded):

- Blood hemoglobin
- RPR test (as per routine antenatal care)
- CD4 cell counts (from fresh maternal blood)
- HIV viral load

In addition, 5 ml of blood may be taken in a sub-sample of study participants for ancillary studies (please refer to section 5.3 for more information on ancillary studies).

For more details on laboratory procedures please refer to section 6.

***h) Administration of IPTp***

Following physical examination, recruited women with more than 13 weeks of gestational age will receive the 1st dose of IPTp (either MQ or Placebo) under supervision.

The trial is a double-blinded study. Both preventive treatments (IPTp-MQ and IPTp-Placebo) will be identically packaged and the pharmacist or independent study site personnel will prepare the medication to the participant after randomization. The investigator and the study participant will remain blinded. *The number of IPTp tablets will be based on bodyweight assuming that each tablet contains 250 mg of active principle (MQ or placebo). The number of MQ tablets administered to women weighing more than 100 kg shall not exceed the maximum dosage limit of 1,5 g which corresponds* ***to 6 tablets.***

**All** women will be observed for **60** minutes after administration of the IPT. Those women vomiting within the first 30 minutes will be given a second full IPT dose; those vomiting from 30-60 minutes will be given an additional half dose.

In addition the tolerability of the study drugs will also be assessed 2 days later after intake by home visits of study personnel. Any medical sign or symptom will be then recorded.

The second dose of IPTp will be given coinciding with the next ANC **visit at least one month apart from the previous dose.**

***i) Administration of CTX***

Cotrimoxazole (a fixed combination drug containing 800 mg of trimetoprim and 160 mg of sulfametoxazole) will be taken daily from enrollment until delivery. **Women will be asked to visit the ANC monthly to receive CTX tablets for the whole month. At these visits, adherence to CTX prophylaxis will be assessed.**

***j) Concomitant medications***

Any concomitant treatment (including antibiotics and other antimalarial drug) will be recorded in the study CRF. Recommended and available treatment for HIV/AIDS will be given by the clinicians involved in the study if indicated, according to the national HIV/AIDS control guidelines.

***k) Long lasting insecticide treated nets***

Regardless of gestational age at the time of recruitment, all women will receive a long lasting insecticide treated net (LLITN) at the time of enrollment and details about its use will be explained.

***l) Follow-up and Household visits***

**Women will be given an appointment to attend the second ANC visit 1 month after the first one. The second IPTp dose should be given at least 4 weeks apart from the 1st one. Study participants will be asked to visit the study facilities in case of any illness. Women will be visited at home the day after recruitment to confirm residence status, assess drugs tolerability and the correct use of the net. Adherence to CTX prophylaxis and compliance with the LLITNs use will be assessed monthly. Participants that will not attend the scheduled site visits will be visited at home by study personnel.**

## ***Visit 2: Administration of 2nd dose of IPTp***

***a) Physical and clinical examination***
Maternal **weight will be measured and gestational age will be assessed. If women passively report to be sick during the ANC visit, they will be referred to the clinical services as per routine system in place. In case of malaria parasitaemia or anaemia, they will be treated following national guidelines (Appendix 3) and the specific study SOPs.**

In addition, 5 ml of blood may be taken in a sub-sample of study participants for ancillary studies (please refer to section 5.3 for more information on ancillary studies).

***b) Administration of IPTp***
Following physical examination, women will receive the 2nd dose of IPTp (either MQ or Placebo) under supervision.

Both preventive treatments will be identically packaged and the pharmacist or independent study site personnel will prepare the medication for the participant after randomization. The investigator and the study participant should remain blinded.

**All** women will be observed for **60** minutes after administration of the IPT. Those women vomiting within the first 30 minutes will be given a second full IPT dose; those vomiting from 30-60 minutes will be given an additional half dose.

In addition the tolerability of the study drugs will also be assessed 2 days later after intake by home visits of study personnel. Any medical sign or symptom will be then recorded.

***c) Medical history***
Any concomitant treatment or history of treatment since the last visit will be recorded in the study CRF. In addition, information on adverse events related to study drugs will be collected.

## ***Visit 3: Administration of 3rd dose of IPTp***

***a) Physical and clinical examination***
**Maternal weight will be measured and gestational age will be assessed. If women passively report to be sick during the ANC visit, they will be referred to the clinical services as per routine system in place. In case of malaria parasitaemia or anaemia, they will be treated following national guidelines (Appendix 3) and the specific study SOPs.**

In addition, 5 ml of blood may be taken in a sub-sample of study participants for ancillary studies (please refer to section 5.3 for more information on ancillary studies).

***b) Administration of IPTp***
Following physical examination, women will receive the 3rd dose of IPTp (either MQ or Placebo) under supervision.

Both preventive treatments will be identically packaged and the pharmacist or independent study site personnel will prepare the medication for the participant after randomization. The investigator and the study participant should remain blinded.

**All** women will be observed for **60** minutes after administration of the IPT. Those women vomiting within the first 30 minutes will be given a second full IPT dose; those vomiting from 30-60 minutes will be given an additional half dose.

In addition the tolerability of the study drugs will also be assessed 2 days later after intake by home visits of study personnel. Any medical sign or symptom will be then recorded.

***c) Medical history***
Any concomitant treatment or history of treatment since the last visit will be recorded in the study CRF. In addition, information on adverse events related to study drugs will be collected.

According to national HIV/AIDS control policies, Nevirapine (and other ARVs) will be given to the pregnant women for the prevention of mother to child transmission (MTCT) of HIV (Appendix 8). Women will be instructed to take the Nevirapine dose when contractions begin.

***d) Follow up***
**Women will be asked to visit the ANC monthly and to deliver at the study health facilities.** Adherence to CTX prophylaxis, as well as compliance with the LLITNs use will be assessed monthly at the scheduled visits. The use of ARV for HIV MTCT prevention will also be recorded. **Participants that will not attend the scheduled site visits will be visited at home by study personnel. In addition, a household visit will be scheduled 2 weeks before the estimated delivery date in order to remind the women to deliver at the study health facilities.**

### ***Visit 4: Delivery/ Newborn assessments***

***a) Maternal clinical examination and assessments***
At the time of delivery or miscarriage, study women will be identified and the outcome of pregnancy recorded into the CRF.

**Blood pressure will be measured** and 5 five milliliters (ml) of venous blood will be collected for haematological determinations and parasitological examination (including PCR filter paper) and for ancillary studies (the latest only on a sub-sample of study participants, please refer to section 5.3 for more information on ancillary studies).

HIV viral load and CD4 count will also be determined at the time of delivery (1 ml blood sample will be drawn). Adherence to ARVs will be monitored.

According to national HIV/AIDS control guidelines, the mother will be referred to the health services for further clinical follow up of herself and her child.

***b) Newborn assessments***
A 5-8 ml blood sample for haematological determinations and parasitological examination (including PCR filter paper) will be collected from the umbilical vein once the baby's umbilical cord has been cut and clamped.

The newborn will be physically examined, weighed and measured and his/her gestational age assessed by the Ballard method (Appendix 4). Date of birth, sex of the infant, axillary temperature and any observed congenital abnormalities will be recorded into a specific designed CRF.

According to national guidelines for HIV MTCT prevention, ARVs will be administered to the newborn before discharge.

***c) Placental samples***
The placental biopsy will be placed in 10% neutral buffered formalin to prevent clotting and kept in a cool box until its transport to site's laboratory.

SOPs will be written to detail the procedures for placental samples processing and storage. Placenta samples will be processed at the site laboratory (please refer to section 6 for more details).

***d) Follow up***

Newborns will be given a study number independent from the mother's study number in order to be uniquely identified. Both mother and child study numbers will be recorded in the CRF. The newborn study number will be a combination of the site number and participant number.

The site code will be assigned by CRESIB and the participant number by the investigator. At each site the first newborn will be assigned a participant number, and consecutive numbers will be assigned to subsequent newborns.

The mother will be asked to come back to the study health facilities after 1 month of delivery, coinciding with the 1st routine immunization visit of the newborn.

***e) Home deliveries***

Women who deliver at home will be visited within one week of delivery and details of the delivery outcome will be recorded. **Newborn's weights captured 1 to 7 days after birth will be "adjusted" to estimate birth weight using a statistical factor used in previous studies conducted among African newborns[37] .**Women will be asked to visit the study health facilities with their babies. At this visit a capillary blood sample (heel prick) will be taken, a thick blood smear and filter paper collected from both mother and infant and the weight of the baby measured. The newborn's gestational age will be assessed following Ballard's method.

Women delivering at home will be invited to attend the study health facilities to determine HIV viral load and CD4 count after delivery.

***f) Multiple births***

In case the outcome of the pregnancy is multiple births, the first born infant will be assigned the first available consecutive study number. His/her sibling(s) will receive the following consecutive number. Newborns will be followed according to the described procedures of the study until they are 2 months old.

***g) Miscarriage and other pregnancy outcomes***

If the outcome of the pregnancy is a miscarriage or stillbirth, women will be visited at the study facilities and haematological and parasitological tests will be performed.

***Visit 5: One month after delivery***

**Study participants will be invited to attend the study health facility one month after delivery coinciding with the first scheduled EPI visit of the infant. Women will be clinically examined: axillary temperature will be measured and a capillary blood sample collected for haematological (Hb) determination and parasitological (thick blood smear and filter paper) examination. In case of malaria parasitaemia or anaemia, they will be treated following national guidelines. Adherence to ARVs for prevention of HIV MTCT will also be assessed**.

***Unscheduled visits:***

Study participants reporting sick at the health facilities will be seen by study personnel. Every unscheduled visit of the woman from enrollment until 2 months after delivery will be recorded into a CRF.

***a) Physical and clinical examination***

Gestational age, **blood pressure** and axillary temperature will be measured. Patients with fever (≥37,5ºC) or history of fever in the last 24 hours, **headache or arthromyalgias** or who appear pale

will have a thick and thin blood film done and an haematocrit collected. In case of malaria parasitaemia or anaemia, they will be treated following national guidelines (Appendix 3).

***b) Other medications***
Any concomitant treatment or history of treatment since the last visit will be recorded in the study CRF. In addition, information on adverse events related to study drugs will be collected.

***Outline of the maternal visits (HIV infected women):***

From enrollment until delivery, pregnant women will be involved in the study procedures according to the following schedule.

**Table 5. Maternal visits and procedures schedule**

| **Study procedure** | **Visit 1 Enrollment** | **Visit 2** | **Visit 3** | **Visit 4 Delivery** | **Visit 5 1 month** | **Unscheduled visits** | **Household visits** |
|---|---|---|---|---|---|---|---|
| Inclusion/ Exclusion criteria check | X | | | | | | |
| Written informed consent | X | | | | | | |
| Demographics/ Medical history | X | | | | | X | |
| Record of concomitant medication / Adverse events | X | X | X | X | X | X | |
| Physical examination/clinical | X | | | | | X | |
| Gestational age | X | X | X | X | | X | |
| Temperature | | | | | X | X | |
| **Blood Pressure** | | | | X | | X | |
| Weight | X | X | X | | | X | |
| Height | X | | | | | | |
| MUAC | X | | | | | | |
| IPTp administration | X | X | X | | | | |
| CTX administration | X | * | * | * | | * | * |
| RPR test | X | | | | | | |
| HIV test | X | | | | | | |
| CD4 count | X | | | X | | | |
| Viral load | X | | | X | | | |
| Blood smear | † | † | † | X | X | † | |
| Haemoglobin test | X | | | X | X | | |
| Peripheral venous blood (mother) | | | | X | | | |
| Cord blood sample | | | | X | | | |
| Placental biopsy | | | | X | | | |
| Placental impression smears | | | | X | | | |
| **Drug tolerability assessment** | X | X | X | | | | X |
| LLITNs | X | ‡ | ‡ | ‡ | ‡ | ‡ | ‡ |
| Ancillary studies | § | § | § | § | | | |

* CTX adherence should be assessed at each scheduled visit.
† Only in women passively reporting sick AND presenting with malaria related signs/symptoms (fever (≥37,5º C) or having history of fever in the past 24 hours, arthromyalgias or headache), as per national management guidelines ‡ Assessment of compliance with LLITNs use § Blood sample may be taken in a sub-sample of study participants for ancillary studies (please refer to section 5.3 for more information on ancillary studies). *Filter paper will be collected at unscheduled visits (fingerprick), at delivery (from venous blood, cord blood and placental blood) and at the unscheduled visit 1 month after delivery (fringerprick).*

*The allowed to timeframe for conducting the scheduled visits is shown in the trial flow (refer to Appendix 12).*

## ***Follow up of study children:***

Children born from study participants will be followed up until 2 months of age. Mothers will be asked to bring their child to the study facilities at month 1 (or coinciding with the 1st EPI visit) and 2 after birth.

***a) Site visits assessments:***
At month 1 (or coinciding with 1st EPI visit) and 2 after birth, study children will be physically examined by study personnel. The psychomotor and neurological development of the infant will be assessed following a simplified protocol (Appendix 5). Weight, height and axillary temperature will be measured and recorded.

A capillary blood sample will be taken from infants with fever (axillary temperature ≥37,5ºC) or history of fever in the last 24 hours, or appearing pale, for malaria parasitemia examination and haematological determination. In case of malaria parasitaemia or anaemia, they will be treated following national guidelines (Appendix 6).

***b) HIV exposure***
One month after birth, HIV DNA will be evaluated in the infant from dried blood spot on filter paper (for PCR). The mothers will be encouraged to bring their children at month 18 after birth to repeat the test according to national guidelines.

Study infants will be followed until 2 months of age. As per routine healthcare national programs HIV-exposed infants should receive CTX prophylaxis starting four to six weeks after birth (and maintained until HIV infection can be excluded, month 18). Children will receive ARV when needed, according to national guidelines.

***c) Household visits***
Study infants that will not attend the scheduled site visits will be visited at home by study personnel. The psychomotor development of the infants will be assessed following a simplified protocol and recorded into the CRF. Compliance with the use of LLITNs the previous night will also be assessed.

Demographic events such as migrations and deaths will be documented through the DSS (whenever in place in the study site)

***d) Unscheduled visit***
Throughout the 2 months follow-up, the study infants reporting sick at the health facilities will be seen by project personnel. They will be physically examined. Weight, height, and axillary temperature will be measured and recorded.

Patients with fever (axillary temperature ≥37,5ºC) or history of fever in the last 24 hours or who appear pale will have a thick and thin blood film done. Blood hemoglobin will also be determined. In case of malaria parasitaemia or anaemia, they will be treated following national guidelines (Appendix 6).

***<u>Outline of the infants visits:</u>***

From delivery until month 2, study infants will be involved in the study procedures according to the following schedule.

**Table 6. Infants visits and study procedures schedule**

| **Study procedure** | **Birth** | **1 m*** | **2 m** | **Unscheduled visits** |
|---|---|---|---|---|
| Medical history | X | X | X | X |
| Physical examination | X | X | X | X |
| Psychomotor development assessment | X | X | X | |
| Weight | X | X | X | X |
| Height | X | X | X | X |
| Temperature | X | X | X | X |
| Blood smear | X | † | † | † |
| Haemoglobin test | X | † | † | † |
| HIV PCR$^{\pm}$ | | X | | |
| Ancillary studies | | ‡ | ‡ | |

m: month; * First visit will be scheduled 1 month after birth or coinciding with 1$^{st}$ EPI visit † Only if fever (≥37,5º C) or history of fever in the past 24 hours or signs suggestive of malaria. $^{\pm}$ HIV PCR test should be repeated at month 18 after birth. ‡ Blood sample may be taken in a sub-sample of study participants for ancillary studies (please refer to section 5.3 for more information on ancillary studies).

*<u>The allowed to timeframe for conducting the scheduled visits is shown in the trial flow (refer to Appendix 12).</u>*

Study infants that will not attend the scheduled site visits will be visited at home by study personnel. Demographic events such as migrations and deaths will be documented through the DSS (whenever in place in the study site). As the minimum, all the study mothers and her children will be visited at home 2 months after delivery for final assessments of status.

### 5.3 Ancillary studies:

This project will work under the umbrella of the MiP consortium where "Cross-cutting" activities are planned to be conducted among the different projects.

These include molecular, immunological and histopathological ancillary studies. In brief, the objectives of the ancillary studies will be to assess:

- the impact of interventions on immunity to malaria in pregnancy
- the interaction between interventions and drug resistance and the importance of malaria in pregnancy
- the impact of interventions on histological evidence of malaria

Specific study protocols will be developed and after obtaining ethical clearance, specific blood samples may be collected in a sub-sample of the study participants according to the previous schedule.

### 5.4 Withdrawal criteria

Women will be **withdrawn** from the study if they meet any of the following criteria:

- withdraws from informed consent
- migrates out of the study area for more than three months *during her pregnancy and does not deliver at the study health facilities (end of pregnancy data are lacking)*
- does not come to the postpartum visit (drop out)

A *treatment withdrawal* will be any pregnant women presenting *an adverse effect due to the intervention and which severity contraindicates an additional dose according to the study clinician*. In this case the woman will continue taking part in all study procedures and follow up except that she will not receive further interventions.

All participants will be followed up until the end of the follow up unless meeting the study withdrawal criteria.

### 5.5 Protocol deviation/ exception/ violation

A ***protocol deviation*** is any alteration/modification to the IRB-approved protocol. The protocol includes the detailed protocol, protocol summary, consent form, recruitment materials, questionnaires, and any other information relating to the research study.

A ***protocol exception*** is any temporary protocol deviation that is approved by the IRB prior to its initiation, e.g., enrollment of a subject who does meet the eligibility criteria.

A ***protocol violation*** is any exception/variance in the conduct of the research that is not approved by the IRB prior to implementation and is not consistent with the protocol that has been reviewed and approved by the IRB. Protocol violations also include non-compliance with Good Clinical Practices (GCP), or protocol-specific Standard Operating Procedures (SOP) requirements. The noncompliance may be either on the part of the participant, the investigator or the study site staff.

Protocol violations can be:

*Major*: a violation that may impact participant's rights, safety and welfare, may alter risk to participants or as determined by the IRB.

*Minor*: a violation that does not impact participant safety or substantially alter risk to participants as determined by the IRB.

A protocol violation may also occur when an event happens that does not allow for accurate interpretation of preventive treatment efficacy.

As a result of violation, corrective actions are to be developed by the site and implemented promptly. The project will follow GCP guidelines for recording and reporting protocol violations.

# 6 Laboratory procedures

## 6.1 Parasitological and haematological determinations

Thick and thin blood smears will be stained with Giemsa's stain and examined following standard procedures. Blood haemoglobin will be determined following local SOPs.

Maternal and cord blood will be separated and the plasma, white and red cells stored frozen.

Standard Operating Procedures (SOP) will be written to detail the procedures for sample processing and storage.

## 6.2 Histological analysis of the placenta

A tissue sample (approximately 2 $cm^3$) will be collected from the maternal surface of the placenta in an off-center position, a quarter of the distance between the umbilical cord and the edge, from an area without signs of infarction[39]. The biopsies will be immediately placed in 25 mL of 10% neutral buffered formalin and kept at 4ºC until processed and embedded in paraffin wax by standard techniques[40].

Paraffin sections 4um thick from the placental tissue, will be stained with hematoxylin and eosin, Giemsa's stain and the periodic acid-Schiff technique.

Placentas will be classified histologically as: i) not infected, ii) active infection and iii) past infection, depending on the presence or absence of parasites, pigment or both.

Impression smears will be prepared from the placental blood for parasitological examination. Blood from the placenta will also be collected onto filter paper for PCR determination of malaria parasites.

Standard Operating Procedures (SOP) will be written to detail the procedures for sample processing and storage.

## 6.3 Detection of HIV and quantitative determination of viral load

Quantitative PCR HIV viral load will be determined for mothers from the blood samples drawn at enrollment and delivery. Vertical transmission of HIV will be determined by qualitative DNA PCR performed on samples drawn from infants at 1 month and 12-18 months.

## 6.4 Immunological determinations related to HIV status

CD4 counts will be determined using CD3, CD8 and CD4 fluorochrome labelled antibodies on an aliquot of whole blood[41]. The exact count is obtained on a two platform system using the whole

white blood cell count obtained by hemogram analysis and the count of the CD4 subset of CD3 cells obtained by flow cytometry on a Becton Dickinson FACSCalibur[42].

# 7 Safety

The study is designed as a cohort study in which participants (pregnant women) will be followed up prospectively. Exposure of pregnant women to antimalarials (and malaria) carries risks (and benefits) to both mother and developing foetus. However, all the antimalarials proposed for use in these trials have previously been used in 2nd and 3rd trimester of pregnancies.

Pharmacovigilance systems will be established or improved to carry out intensive monitoring in order to adequately assess the safety of the study drugs. Safety will be evaluated in the mother, the foetus and the infant.

Adverse events will be monitored throughout the study to assess the general safety and tolerability of the preventive treatments.

## 7.1 Adverse Events

A Standard Operating Procedure (SOP) will be written to outline for research staff the procedures for recording, reporting and management of Adverse Events (AE) and Serious Adverse Events (SAEs). CRESIB will ensure that AEs and SAEs are reported in compliance with International Conference on Harmonisation (ICH) Good Clinical Practice (GCP) guidelines.

*Definition of an Adverse Event (AE)*[43]*:*
Any untoward medical occurrence in a subject to whom a medicinal product has been administered, including occurrences which are not necessarily caused by or related to that product.

An AE can therefore be any unfavorable and unintended sign (including a clinically significant abnormal finding), symptom or disease temporally associated with the use of a medicinal product, whether or not considered related to the medicinal product.

The occurrence of an adverse event may come to the attention of study personnel during study visits, home visits, telephone calls, and interviews or by a study recipient presenting for medical care, or upon review by a study monitor.

All AEs should be treated appropriately. Treatment may include one or more of the following:
- no action taken (i.e. further observation only)
- study drug dosage adjusted/temporarily interrupted
- study drug permanently discontinued due to this adverse event
- concomitant medication given
- non-drug therapy given
- patient hospitalized/patient’s hospitalization prolonged.

The action taken to treat the adverse event will be recorded on the AE form. Once an adverse event is detected, it will be followed until its resolution or until it is judged to be permanent, and assessment will be made at each visit (or more frequently, if necessary) of any changes in severity, the suspected relationship to the study drug, the interventions required to treat it, and the outcome.

Information about common side effects already known about the study drug can be found in the Appendix 1. This information will be included in the patient informed consent and will be discussed with the patient during the study as needed.

### 7.2 Severity, relationship of event to study drug, and outcome

The **severity** of a clinical adverse event is to be scored according to the following scale:

| Grade | Severity | Description |
|---|---|---|
| 1 | Mild | Awareness of sign or symptom, but easily tolerated |
| 2 | Moderate | Discomfort enough to cause interference with usual activity |
| 3 | Severe | Incapacitating with inability to work or perform usual activity |
| 4 | Life-threatening | Patients at risk of death at the time of the event |

In addition, all adverse events will be assessed and documented by the investigator using a toxicity table that will be developed as SOP. All grade 3-4 toxicities are considered serious. Changes in the severity of an AE should be documented to allow an assessment of the duration of the event at each level of severity. Adverse events characterized as intermittent will provide documentation of onset and duration of each episode.

The **relationship** of an adverse event to study drug is to be assessed according to the following definitions:

*Definitely unrelated*:
It should be reserved for those events which occur prior to test drug administration (e.g., washout or single-blind placebo) or for those events which cannot be even remotely related to study participation (e.g. injury caused by a third party).

*Unlikely:*
There is no reasonable temporal association between the study drug and the suspected event and the event could have been produced by the subject's clinical state or other modes of therapy administered to the subject.

*Possible:*
The suspected adverse event may or may not follow a reasonable temporal sequence from study drug administration but seems to be the type of reaction that cannot be dismissed as unlikely. The event could have been produced or mimicked by the subject's clinical state or by other modes of therapy concomitantly administered to the subject.

*Probable*:
The suspected adverse event follows a reasonable temporal sequence from study drug administration, abates upon discontinuation of the drug, and cannot be reasonably explained by the known characteristics of the subject's clinical state.

*Definitely related*
It should be reserved for those events which have no uncertainty in their relationship to test drug administration: this means that a re-challenge was positive.

The **outcome** of each AE must be assessed according to the following classification:

- Completely recovered : the patient has fully recovered with no observable residual effects
- Not yet completely recovered : improvement in the patient's condition has occurred, but the patient still has some residual effects

- Deterioration : the patient's overall condition has worsened
- Permanent damage : the AE has resulted in a permanent impairment
- Death : the patient died due to the AE
- Ongoing : the AE has not resolved and remains the same as at onset
- Unknown : The outcome of the AE is not known because the patient did not return for follow-up (lost to follow-up)

### 7.3 Definition of Serious Adverse Event (SAE):

A SAE is an adverse event that:

- Results in death
- Is life threatening
- Requires hospitalization or prolongation of existing hospitalization
- Results in persistent or significant disability or incapacity or
- Consists of a congenital anomaly or birth defect

The Principal Investigators at each study site will be responsible for recording, management, and reporting of the AEs and SAEs. They must be recorded in the AE/SAE CRF with the following information:

- The severity grade (mild, moderate, severe)
- Relationship to the study drug(s) (suspected/not suspected)
- Duration (start and end dates or if continuing at final exam)

*Definition of Suspected Unexpected Serious Adverse Reaction (SUSAR):*
A serious adverse event (SAE) becomes a SUSAR (suspected unexpected serious adverse reaction) if the event is suspected (possibly, probably or definitely) to be related to the study drug and unexpected i.e. not previously documented in any of the product information or protocol.

All SAEs will be **notified within 48 hours to the sponsor (CRESIB) and the initial SAE report sent within 72 hours to the Data Safety Monitoring Board (DSMB). In addition, all SAEs forms will be sent to the Safety Working Group and to the independent Safety Panel of the global MiP consortium (with the secretariat based at the Liverpool School of Tropical Medicine)** and to the local ethics committee. For more information on SAEs reporting system refer to Appendix 9.

### 7.4 Monitoring of AEs

Close collaboration among all sites members will be necessary to evaluate study progress and respond to occurrences of toxicity in a timely manner. Rates of accrual, adherence, follow-up, and AE incidence will be monitored closely by the sponsor (FCRB) on a regular basis. The team will meet via conference calls during the period of study implementation. The site Investigator is responsible for continuous close monitoring of all AEs that occur among study participants, and for alerting the rest of the protocol team if unexpected concerns arise. All concerns then will be addressed according to protocol and standard operating procedures

### 7.5 Tolerability/acceptability

Tolerability of the preventive treatment regimens will be assessed by analyzing incidence rates of relevant adverse events (e.g. vomiting during treatment intake, nausea, dizziness, etc).

### 7.6 Data Safety Monitoring Board (DSMB)

A DSMB with independent members will be appointed. The role and responsibilities of the DSMB are defined in a specific SOP. The main role of the DSMB will be to review and analyse clinical safety data collected during the trial and to assess unexpected or serious adverse events (SAEs).

The DSMB will review the implementation and progress of the study, providing advice on safety-related issues before the initiation of the study, during the study and at the close out of the study. Its advice will be based on the interpretation of study data with reference to the study protocol. The DSMB will be empowered to put the study on hold pending review of potential safety issues.

The DSMB will be informed by the investigators of all protocol amendments, informed consent changes or revisions of other documents originally submitted for review, of all subsequent protocol modifications (for information) and of new information that may adversely affect the safety of the subjects or the conduct of the study. The DSMB will also review and approve the Report and Analysis Plan and the DSMB statistician will be responsible of keeping the study code. The DSMB will perform any appropriate statistical calculations to support recommendations to the investigators. All documentation provided to members of the DSMB for information and review will be treated in a confidential manner.

# 8 Data management

A centralized data management and data analysis for the project will be conducted at the site in Manhiça (Mozambique) with the lead of a highly trained statistician. The data management team, with collaboration with all five sites, will develop case report forms (CRFs) and will provide training to all sites on data collection methodology, data quality control (QC), and quality assurance (QA). During the conduct of the study, the study sites will be responsible for management of CRFs and data cleaning, as well as for providing regular reports on recruitment, screening, and enrollment progress of the study.

## 8.1 Case Report Forms

Presentation of the CRF

The CRF to be used for the study consists of pages headed with the study code and other relevant information. It is composed of an introductive part for the selection and inclusion of patients in the study and special forms for the different evaluation times; at the end of the CRF are the forms for registration of possible adverse events and for any suspension of the study. Each page includes a header containing information for the identification on the study subject (Study number and Initials; to be completed by the Investigator) and the study code.

How to use the CRF

It is recommended that the CRF be filled out using a ballpoint pen with black ink. All requested information must be entered on the CRFs. If an item is not available or is not applicable, this fact should be indicated; there should be no blank spaces. A correction should be made by striking through the incorrect entry with a single line and by entering the correct information adjacent to it. The correction must be initialled, dated and explained if necessary by the Investigator or by a qualified individual specifically designated by the Investigator. Each completed Case Report Form must be reviewed, signed and dated by the Investigator.

## 8.2 Quality assurance and quality control

Implementation of this study will be directed by the protocol as well as a study specific SOPs. Quality assurance and quality control systems will be implemented and maintain with written SOPs to ensure that trials are conducted and data are generated, documented (recorded), and reported in compliance with the protocol, GCP, and the applicable regulatory requirement(s).

The SOPs will outline procedures for conducting study visits, data and forms processing, AE assessment, management and reporting, dispensing study drugs and documenting drugs accountability, and other study operations.

Close cooperation between the sponsor, investigators, biostatistician, data managers, and other protocol team members will be necessary in order to track study progress, respond to queries about proper study implementation, and address other issues in a timely manner. Assignment of all sponsor and investigator responsibilities for this project will be specified in a MiP Consortium Agreement.

### 8.3 Site monitoring

Before study initiation, at the site initiation visit or at the first investigator's meeting, the protocol and CRFs will be reviewed by all the investigators and their staff.

During the study, the clinical monitor will visit the site regularly to check the completeness of participants records, the accuracy of entries on the CRFs, the adherence to the protocol and to Good Clinical Practice, the progress of enrollment, and to ensure that study drugs are being stored, dispensed, and accounted for according to specifications. Key study personnel must be available to assist the site monitor during these visits.

The site investigator will maintain source documents for each participant in the study, consisting of case and visit notes (hospital or clinic medical records) containing demographic and medical information, laboratory data and the results of any other tests or assessments. All information on CRFs must be traceable to these source documents in the patient's file. The investigator must also keep the original informed consent form signed by the participant (a signed copy is given to the patient).

The investigator will give the monitor access to all relevant source documents to confirm their consistency with the CRF entries. International monitoring standards require full verification for the presence of informed consent, adherence to the inclusion/exclusion criteria, documentation of SAEs, and the recording of data that will be used for all primary and safety variables. Additional checks of the consistency of the source data with the CRFs will be performed according to the study-specific monitoring plan. The monitor will also inspect study facilities and regulatory documentation. No information in source documents about the identity of the participants will be disclosed.

Following a monitoring visit the monitor will send a report to the sponsor and the study site. In the event that major violations are identified, the site will be asked to provide within 30 days a plan to correct deficiencies. If needed, a repeat site visit will be conducted.

A site visit log will be maintained at the study site to document all visits.

### 8.4 Data collection

Designated investigator staff must enter the information required by the protocol onto the CRFs that are printed on 3-part, non-carbon-required paper. The CRF should not be used as a Source Document. Each completed CRF must be reviewed, signed and dated by the site investigator.

Site monitors will also review the CRFs for completeness and accuracy and instruct site personnel to make any required corrections or additions. The CRFs will be forwarded to the Centro de Investigaçao em Saúde da Manhiça (CISM) in Mozambique by site monitors or by the designated staff at the site, one copy of CRF being retained at the study site. Once the CRFs are received at the CISM, their receipt will be recorded, the original copy placed in Central Files, and the non-carbon-required copy is forwarded to the responsible CISM data management staff for processing.

### 8.5 Database management

Data from the CRFs will be double entered into the study database at the CISM by data clerk Mozambican staff, following internal standard operating procedures.

Subsequently, the entered data are systematically checked by Data Management staff, using error messages printed from validation programs and database listings. Obvious errors are corrected by Data Management personnel. Other errors or omissions are entered on Data Query Forms, which are returned to the study site for resolution. The signed original and resolved Data Query Forms are kept with the CRFs at the study site, and a copy is sent to the CISM so the resolutions can be entered into the database. Quality control audits of all key safety and efficacy data in the database are made prior to locking the database.

Concomitant medications entered into the database will be coded using the WHO Drug Reference List, which employs the Anatomical Therapeutic Chemical classification system. Medical history/current medical conditions and adverse events will be coded using the Medical dictionary for regulatory activities (MedDRA) terminology.

## 9 Data analysis

Data analysis will be conducted at the CISM (Mozambique) by a team of statisticians lead by the Biostatistics Unit from CRESIB. The final data analysis plan will be developed in collaboration with all study sites **through regular meetings of a statistics working group.**

### 9.1 Populations for analysis

The assignment of participants into analysis populations will be performed prior to database lock and any data analysis. The following populations are defined:

*Intention-to-treat (ITT)* – all randomized women.

Following the intention-to-treat principle, patients will be analyzed according to the preventive treatment they were assigned to at randomization.

*According to protocol (ATP)* - all women who fulfill all the inclusion-exclusion criteria and took the two IPT doses, received the LLITN and from whom data is available for the analysis.

*Safety* – all patients that received at least one dose of IPT and had at least one post-baseline safety assessment. Patients will be analyzed according to preventive treatment received.

### 9.2 Trial Profile

A trial profile will be done documenting the following information:

- number of screened women
- number of women giving informed consent (and therefore randomized)
- number of women giving an ITN
- numbers randomized to each IPT group
- number of registered deliveries
- number of registered visits after delivery
- number completing follow-up until 2 months after delivery
- number of withdrawals and deaths up to 1$^{st}$ dose of IPT
- number of withdrawals between doses 1 and 2
- number of withdrawals between 2$^{nd}$ dose until delivery
- delivery outcomes

### 9.3 Patient demographics/other baseline characteristics

All data will be listed and will consist of data collected/measured prior to randomization. Background and demographic characteristics of patients will be summarized for each IPT group using all randomized patients.

Variables included in this analysis will be:

- Maternal age
- Gestational Age (mean and by category, at recruitment)
  - First trimester 0-12 weeks
  - Second trimester 13-24 weeks
  - Third trimester > or = 25 weeks
- Parity (mean and by category)
  - Primigravidae (no previous pregnancy)
  - 1 to 3 previous pregnancies
  - 4 or more previous pregnancies
- Region/Country
- HIV status
- CD4 count (in HIV-infected women)
- Viral load (in HIV-infected women)
- MUAC index
- Literacy (read and/or write)
- Previous visits to the ANC before the recruitment
- Height (cm)
- RPR results at 1st antenatal clinic
- Hb results at 1st antenatal clinic (g/dl)

Continuous variables will be summarized by sample size, mean, median, standard deviation, minimum and maximum. Discrete variables will be summarized by frequencies and percentages.

In assessing comparability between groups, more emphasis will be given to the size of any differences than to statistical significance, as well as to the relationship with the outcome, since this is what affects the degree of confounding.

Descriptive statistics such as frequency counts and percentages of patients who use concomitant therapies will be provided by IPTp group.

### 9.4 Statistical models and methods of analysis:

**Given the long half life of MQ, it has been assumed that MQ efficacy to prevent LBW will be equal in the full dose and split dose groups.**

**The statistical analysis will follow a sequential approach. First, hypotheses of non-inferiority between the proportion of LBW in the MQ groups and the SP group will be tested assuming a 25% reduction in LBW prevalence. If non-inferiority between MQ and SP is achieved, then an analysis for superiority will be conducted comparing the groups.**

**The primary analysis will be adjusted by gravidity and by covariates associated with LBW, and will be done in the per protocol cohort.**

A crude table with the number of subjects that have at least one malaria episode and the time at risk by IPTp group will be present as well as tables stratified by parity and baseline variables.

The effect of the MQ will be evaluated using the Protective Effect (PE) estimated as (1-HR)*100. The Hazard Ratio (HR) will be estimated using Cox proportional regression models.

The effect of time on efficacy since 1st dose of IPTp will be estimated using time versus treatment interaction in a Cox time-dependent model.

In the secondary analyses, crude and adjusted prevalence will be calculated. Likelihood Ratio tests will be calculated to evaluate the interaction between the intervention group and parity. Differences in prevalence will be estimated with the $\mathcal{X}^2$ test and proportions with the Fischer's exact test **and adjusted by covariates using regression models.** Continuous values will be evaluated with the non-parametic Wilcoxon test. Data analysis will be performed using Stata (Stata Corporation, College Station, TX,USA).

The final data analysis plan will be developed in collaboration with all study sites.

### 9.5 Safety

All analyses of safety will be based on the safety population.

Adverse events will be summarized by presenting, for each IPTp group, the number and percentage of paticipants having any AE. Any other information (e.g. severity or relatedness to study treatment) will be listed, as appropriate. Severe AE's (including deaths) will be listed by IPTp group.

Any statistical tests performed to explore the safety data will be used only to highlight any interesting comparisons that may warrant further consideration.

### 9.6 Tolerability

Tolerability will mainly be assessed by displaying and contrasting the number of patients that have AE's that either lead to drug interruption or have AE's that lead to early discontinuation. **The incidence of side effects (mainly vomiting and dizziness) in the MQ single intake and MQ split dose groups will be compared.**

## 10 Investigator responsibility

The clinical trial is constituted by collaborators from the 5 sub-Saharan sites involved in the project. The partners have been carefully selected to assure the ability to carry out the project with rigorous, standardised methodologies and adequate sample sizes.

The responsibilities of the site principal investigator will be:

- To ensure that approval(s) from the relevant approved local ethics committee are obtained
- To read and accept the relevant information package developed by the sponsor for clinical studies
- To have good knowledge of the protocol, related documents and the regulatory requirements of the regulatory authority (ies) and other relevant legislation
- To read, understand and agree to work according to the protocol, the Declaration of Helsinki, ICH Guidelines for Good Clinical Practice and other relevant legislation and to ensure that the study follows GCP guidelines
- To undertake the use of the investigational and comparator drug (s) only for the purposes of the study as described in the protocol
- To take responsibility for accountability of the investigational drug (s)
- To document clearly the sequences of events to be followed in the conduct of the clinical trial, including timeframes, roles and responsibilities
- To ensure the availability of all necessary facilities, equipment, and finance to conduct the trial
- To develop proper mechanisms to ethically obtain informed consent of participants

- To accept the involvement of monitors to review and verify the quality control procedures and conduct data verification
- To accept the possibility of audit and/or inspection by an independent auditor appointed by the sponsor, regulatory authority or ethics committee
- To lead and oversee all study activities
- To ensure coordination with the project Coordinator and the Project Manager at FCRB, Barcelona
- To oversee and supervise all study staff and provide regular verbal and written feedback to staff members regarding their performance
- To communicate with all study staff regularly at scheduled meetings as well as informally
- To review budgets and approve expenditures charged to the site
- To respond to queries, concerns and issues raised by the sponsor and the study participants, the community, the project staff, and other parties involved
- To participate in hiring personnel for the study
- To ensure that all project activities are carried out according to the protocol and that deadlines are met.
- To ensure that participant privacy and confidentiality are maintained
- To report activities, problems, and needs regularly to the project manager
- To ensure proper safety reporting procedures: report AEs and SAEs to the sponsor (FCRB) in the time defined in the protocol and SOPs
- To assist the study coordinator in developing and maintaining a Community Advisory Group (if applicable) for the study and ensure that community concerns are addressed by the study staff and leadership.
- To ensure that correct information about the study is relayed to the study participants and the general community in a timely manner.
- To maintain close professional relationships with the Provincial Medical Officer, District Medical Officer, Municipal Medical Officer and other officials within the Ministry of Health (if applicable)
- To work with study coordinator to ensure that study specific SOPs are complete and regularly updated
- To prepare and to present study at scientific meetings
- To work in a professional and ethical manner with competence, accountability, and integrity

# 11 Ethical considerations

This clinical study was designed and shall be implemented and reported in accordance with the ICH Harmonized Tripartite Guidelines for Good Clinical Practice, with applicable local regulations and with the ethical principles laid down in the Declaration of Helsinki (Appendix 11).

## 11.1 Informed consent

Informed consent is a process that is initiated prior to the individual's agreeing to participate in the study and continuing throughout the individual's study participation. Consent form will be IRB approved and the women will be asked to read (or will be read to them) and review the document.

The consent form for the study has been designed to provide information on the following aspects: general malaria education, information about one's rights as a study participant, what samples are collected, what the samples will be used for, the schedule of follow-up visits, confidentiality of personal data collected and potential risks and benefits of the study. It has also been specified that participants have the right to leave the study whenever they want and that this does not affect the clinical care the patient will receive thereafter (see Appendixes 2 and 7). The form is thorough but

easily understood. Consent forms will be translated into in the local language and the information within the consent form will be modified according to the needs of each study site.

Both oral and written communication will be used to provide information to pregnant women who are interested in participating in the study. If a woman is interested in participating, she will be informed about the objective and procedures of the study and about the risks and benefits of enrolling herself in the study. A study staff, specifically trained, will review the informed consent form with her. Ample time will be given for consideration of the consent forms, and any questions will be directed to the investigator before the consent is signed.

The informed consent will only be signed after the woman has been able to answer an oral comprehension test with questions on the main study aspects. If the woman is not able to answer the main questions, these will be re-explained to her until she is able to answer the oral comprehension test correctly. Women who will not be able to answer the oral comprehension test will not be enrolled into the study.

For those women who are illiterate and unable to provide a signature, a thumbprint will be utilized as an alternative. In all cases, informed consent may be documented only by the dated signature (or thumbprint) of the volunteer and signed by an independent witness. This signature (or thumbprint) provides documentation that the information about the study presented to the volunteer has been comprehended. If the woman is under 18 years of age, national guidelines regarding participation of minors in clinical trials will be followed. The informed consent will cover the woman and the new born infant.

Two identical copies of the consent form will be signed/thumbprinted. One of the copies will be provided to the volunteer and the other copy will be kept on file by the investigator on site.

### 11.2 Participant confidentiality and data protection

Data will be collected prospectively as part of the study into the specific standardised questionnaires (CRFs). Laboratory samples will be identified by a unique identification number and will not contain the name of the patient, ensuring confidentiality in the handling of blood samples. Questionnaires containing both the names of participants and the samples' identification numbers will be kept in locked file cabines, separate from those handling the samples. Questionnaires will be entered electronically into a database.

The system is designed to protect the confidentiality (all personal data is automatically encrypted) and integrity of the data and includes authorization, authentication, auditing and availability features to safeguard the access and usage of the data. Physical questionnaires containing the names and personal data of participants will then be stored in a secure place and only investigators and designated study staff will have access to them.

There is also risk of loss of confidentiality during the tracing of participants for follow up. We will minimize risk by using only those methods of contact that are acceptable and applicable to participants. Outreach workers will avoid discussing any study specifics when trying to locate participants at home, and to approach contacts in neutral terms. We will speak only to people – including family members – to whom participants have granted us permission to speak.

Participants' study information will not be released without the written permission of the participant, except as necessary for sponsor, site monitoring, and other regulatory authorities.

### 11.3 Confidentiality for HIV-seropositive participants

After accepting and attending HIV voluntary counseling and testing (VCT), women will give their informed consent to participate in the study. Confidentiality is ensured by a VCT identification number that only allows the testing and counseling center to link the VCT number and the study number.

The investigators will have the study number on the questionnaire which does not include the subject's name. With regards to breastfeeding, HIV seropositive mothers will be counseled according to the national recommendations.

In countries with limited resources, guidelines regarding available HIV/AIDS treatment are constantly evolving. The current protocol will take into account changes and integrate national guideline modifications and administer new treatments as they are approved by the national governments.

With regards to prevention of HIV transmission, participants will receive counseling and information about HIV prevention in the voluntary testing and counseling center.

### 11.4 Ethics Committee review

This protocol, the associated informed consent documents, recruitment materials, and other requested documents will be reviewed and approved by the Ethics Committee of the Hospital Clínic in Barcelona, the IRB from the CDC and local ethical review bodies responsible for oversight of research conducted at the study sites with respect to scientific merit and compliance with all research and human subject regulations.

The study protocol is planned to be submitted to all the regulatory authorities and ethics committees between October to December 2008.

In the event of significant changes are requested by one IRB amended protocols will be submitted to all participating ethics committees so that only one version of the protocol exists in all sites and that version is approved.

## 12 Timeframe

The recruitment phase is expected to extend at least over 30 months. Follow-up of the study cohorts will have to last at least until the infant is one year-old. The field work of the study is expected to extend over two and a half years.

## 13 Administrative procedures

### 13.1 Consortium agreement

Prior to the start of the trial a consortium agreement will be signed between all the consortium partners. The consortium agreement will be the main contractual document for the project.

Each Consortium partner will be required to accede to it and it will cover such issues as intellectual property, liability, insurance and indemnity and processes for dealing with default in the delivery of objective/meeting milestone (as well as other contractual requirements).

### 13.2 Insurance

A liability insurance has been taken and will cover all study participants.

### 13.3 Dissemination of results

A project communication plan will be developed in order to ensure timely, accurate, and effective communication among partners and key stakeholders. The plan will outline responsibilities for data management and sharing and communication with the external community, as well as a publication strategy. Agreement will be sought on how and to whom information will be shared to best meet the project and public awareness goals.

After concluding the trial analysis of all relevant data, results will be made available to all partners and key stakeholders. The project members will actively promote sharing and dissemination of results within the scientific community and beyond. The project members will disseminate information to the scientific community through reports, scientific publications in international journals, presentations at scientific forums.

## 14 References

[1] World Health Organization (WHO). A strategic framework for malaria prevention and control during pregnancy in the African region: report AFR/MAL/04/01. Brazzaville, Democratic Republic of Congo: WHO Regional Office for Africa,2004.

[2] Steketee RW, Nahlen BL, Parise ME, Menendez C. The burden of malaria in pregnancy in malaria-endemic areas. Am J Trop Med Hyg 2001; 64(1-2):28-35.

[3] Nosten F, Rogerson SJ, Beeson JG et al. Malaria in pregnancy and the endemicity spectrum: what can we learn? Trends Parasitol. 20(9), 425-432 (2004).

[4] Menendez C. Malaria during pregnancy. Curr Mol Med 2006; 6(2):269-273.

[5] Greenwood BM, Greenwood AM, Snow RW, Byass P, Bennett S, Hatibnjie AB. The Effects of Malaria Chemoprophylaxis Given by Traditional Birth Attendants on the Course and Outcome of Pregnancy. Trans R Soc Trop Med Hyg 1989; 83(5):589-594.

[6] DAlessandro U, Langerock P, Bennett S, Frances N, Cham K, Greenwood BM. The impact of a national impregnated bed net programme on the outcome of pregnancy in primigravidae in The Gambia. Trans R Soc Trop Med Hyg 1996; 90(5):487-492.

[7] Menendez C, Ordi J, Ismail MR, Ventura PJ, Aponte JJ, Kahigwa E et al. The impact of placental malaria on gestational age and birth weight. J Infect Dis 2000; 181(5):1740-1745.

[8] Brabin B. An Assessment of Low-Birth-Weight Risk in Primiparae As An Indicator of Malaria Control in Pregnancy. Int J Epidemiol 1991; 20(1):276-283.

[9] Granja AC, Machungo F, Gomes A, Bergstrom S, Brabin B. Malaria-related maternal mortality in urban Mozambique. Ann Trop Med Parasitol 1998; 92(3):257-263.

[10] Menendez C, Romagosa C, Ismail MR, et al. An autopsic study of maternal mortality in Mozambique: the contribution of infectious diseases. Plos Med 2008; 5(2): e44.

[11] Bardaji A, Sigauque B, Bruni L, Romagosa C, Sanz S, Mabunda S, Mandomando I, Aponte J, Sevene E, Alonso PL, Menendez C. Clinical malaria in African pregnant women. Malar J 2008;7:27.

[12] Shulman CE, Dorman EK, Cutts F, Kawuondo K, Bulmer JN, Peshu N et al. Intermittent sulphadoxine-pyrimethamine to prevent severe anaemia secondary to malaria in pregnancy: a randomised placebo-controlled trial. Lancet 1999;353(9153):632-6.

[13] Schultz LJ, Steketee RW, Macheso A, Kazembe P, Chitsulo L, Wirima JJ. The efficacy of antimalarial regimens containing sulfadoxine-pyrimethamine and/or chloroquine in preventing peripheral and placental Plasmodium falciparum infection among pregnant women in Malawi. Am.J.Trop.Med.Hyg. 1994;51(5):515-22.

[14] Gamble C, Ekwaru JP, Ter Kuile FO. Insecticide-treated nets for preventing malaria in pregnancy. Cochrane.Database.Syst.Rev. 2006(2):CD003755.

[15] WHO expert consultation on cotrimoxazol prophylaxis in HIV infection. Geneva 2006.

[16] Njagi JK, Magnussen P, Estambale B, Ouma J, Mugo B. Prevention of anaemia in pregnancy using insecticide-treated bednets and sulfadoxine-pyrimethamine in a highly malarious area of Kenya: a randomized controlled trial. Trans R Soc Trop Med Hyg 2003; 97: 277-82.

[17] Menendez C, Bardají A, Sigauque B, Romagosa C, Sanz S, Serra-Casas D, et al. A randomized placebo-controlled trial of intermittent preventive treatment in pregnant women in the context of insecticide treated nets delivered through the antenatal clinic. PLoS ONE 2008;3(4):e1934.

[18] Daramola OO, Alonso PL, O'Dempsey TJ, Twumasi P, McArdle TF, Greenwood BM. Sensitivity of Plasmodium falciparum in The Gambia to co-trimoxazole. Trans.R.Soc.Trop.Med.Hyg. 1991;85(3):345-8.

[19] Mermin J, Lule JR, Ekwaru JP, Pitter C. Should cotrimoxazole prophylaxis be taken by all adults with HIV in Africa? Aids 2005;19(8):845-6.

[20] ter Kuile FO, Parise ME, Verhoeff FH, Udhayakumar V, Newman RD, van Eijk AM et al. The burden of co-infection with human immunodeficiency virus type 1 and malaria in pregnant women in sub-saharan Africa. Am J Trop Med Hyg 2004;71(2 Suppl):41-54.

[21] Oduola A, et al. Innate resistance to new antimalarial drugs in *Plasmodium falciparum* from Nigeria. Trans R Soc Trop Med Hyg 1992; 86:123-6.

[22] Oduola A, et al. Reduced in vitro susceptibility to mefloquine in West-African isolates of *Plasmodium falciparum*. Lancet 1987; ii:1304-5.

[23] MacArthur J et al. Efficacy of mefloquine and sulfadoxine-pyrimethamine for the treatment of uncomplicated *Plasmodium falciparum* infection in Machinga District, Malawi, 1998. Am J Trop Med Hyg 2001; 65:679-84.

[24] Aubouy A, Fievet N, Bertin G, Sagbo JC, Kossou H, Kinde-Gazard D, Kiniffo R, Massougbodji A, Deloron P. Dramatically decreased therapeutic efficacy of chloroquine and sulfadoxine-pyrimethamine, but not mefloquine, in southern Benin. Trop Med Int Health 2007; 12(7):886-94.

[25] Adam I, Ali DA, Alwaseila A, Kheir MM, Elbashir MI. Mefloquine in the treatment of falciparum malaria during pregnancy in Eastern Sudan. Saudi Med J. 2004 Oct;25(10):1400-2.

[26] Matteelli A, Saleri N, Bisoffi Z, et al. Mefloquine vs. Quinine plus sulphalene-pyrimethamine (Metakelfin) for treatment of uncomplicated imported falciparum malaria acquired in Africa. Antimicrobiol Agents Chemotherapy 2005; 49:663-7.

[27] Centers for Disease Control and Prevention. Health Information for International Travel 2005-2006. Atlanta: US Department of Health and Human Services, Public Health Service, 2005.

[28] World Health Organization. International travel and health, 2005. Geneva, Switzerland.

[29] Nosten F, Vincenti M, Simpson J, Yei P, Thwai KL, de Vries A et al. The effects of mefloquine treatment in pregnancy. Clin Infect Dis 1999;28(4):808-15.

[30] Steketee RW, Wirima JJ, Slutsker L, Roberts JM, Khoromana CO, Heymann DL et al. Malaria parasite infection during pregnancy and at delivery in mother, placenta, and newborn: efficacy of chloroquine and mefloquine in rural Malawi. Am J Trop Med Hyg 1996;55(1):24-32.

[31] ter Kuile FO, Nosten F, Thieren M, Luxemburger C, Edstein MD, Chongsuphajaisiddhi T et al. High-dose mefloquine in the treatment of multidrug-resistant falciparum malaria. J Infect Dis 1992;166(6):1393-400.

[32] ter Kuile FO, Nosten F, Luxemburger C, Kyle D, Teja-Isavatharm P, Phaipun L et al. Mefloquine treatment of acute falciparum malaria: a prospective study of non-serious adverse effects in 3673 patients. Bull.World Health Organ 1995;73(5):631-42.

[33] Denoeud L, Fievet N, Aubouy A, Ayemonna P, Kiniffo R, Massougbodji A, Cot M: Is chloroquine chemoprophylaxis still effective to prevent low birth weight? Results of a study in Benin. Malar J 2007, 6:27

[34] Briand V, Denoeud L, Massougbodji A, Cot M. Efficacy of chloroquine prophylaxis versus intermittent preventive treatment to prevent malaria during pregnancy in Benin. J Infect Dis 2008, 198(4):594-601.

[35] Ashley EA, Lwin KM, McGready R, Simon WH, Phaiphun L, Proux S, Wangseang N, Taylor W, Stepniewska K, Nawamaneerat W, Thwai KL, Barends M, Leowattana W, Olliaro P, Singhasivanon P, White NJ, Nosten F. An open label randomized comparison of mefloquine-artesunate as separate tablets vs. a co-formulated combination for the treatment of uncomplicated multidrug-resistant falciparum malaria in Thailand. Trop Med Int Health. 2006 Nov;11(11):1653-60.

[36] Simpson JA, Price R, ter Kuile F, Teja-Isavatharm P, Nosten F, Chongsuphajaisiddhi T, Looareesuwan S, Aarons L, White NJ. Population pharmacokinetics of mefloquine in patients with acute falciparum malaria. Clin Pharmacol Ther. 1999 Nov;66(5):472-84.

[37] Greenwood AM, Armstrong JR, Byass P, Snow RW, Greenwood BM. Malaria chemoprophylaxis, birth weight and child survival. Trans R Soc Trop Med Hyg. 1992 Sep-Oct;86(5):483-5.

[38]UNAIDS/ World Health Organization. Epidemiological Fact Sheets on HIV/AIDS and STIs. Update 2006. Available at http://www.who.int/GlobalAtlas/predefinedReports/EFS2006/EFS_PDFs/EFS2006_KE.pdf

[39] Rasheed FN, et al. Isolation of maternal mononuclear cells from placentas for use in in vitro functional assays. J Immunolo Meth 1992; 146: 185-193.

[40] Ordi J, et al. Massive chronic intervellositis of the placenta associated with malaria infection. Am J Surg Pathol 1998; 22: 1006-11.

[41] Jani, V., G. Janossy, A. Iqbal, F. S. Mhalu, E. F. Lyamuya, G. Biberfeld, D. K. Glencross, L. Scott, J. T. Reilly, V. Granger, and D. Barnett. 2001. Affordable CD4+ T cell counts by flow cytometry. II. The use of fixed whole blood in resource-poor settings. J Immunol Methods 257:145.

[42] Glencross, D., L. E. Scott, I. V. Jani, D. Barnett, and G. Janossy. 2002. CD45-assisted PanLeucogating for accurate, cost-effective dual-platform CD4+ T-cell enumeration. Cytometry 50:69.

[43] ICH. Guidance for Industry: E6 Good Clinical Practice: Consolidated guidance. April 1996. [Cited 5 August 2008]. Accessed from: http://www.fda.gov/cder/guidance/959fnl.pdf .

# 15 Appendices

## Appendix 1. Additional Information on Study drugs (WHO/HTM/MAL/2006.1108)

### 1. Sulfadoxine-pryimethamine

**Sulfadoxine**

Molecular weight: 310.3

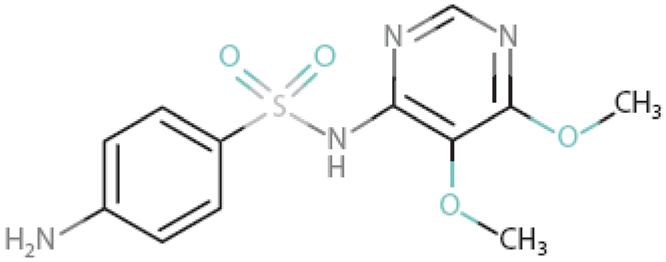


Sulfadoxine is a slowly eliminated sulfonamide. It is very slightly soluble in water. Sulfonamides are structural analogues and competitive antagonists of p-aminobenzoic acid. They are competitive inhibitors of dihydropteroate synthase, the bacterial enzyme responsible for the incorporation of paminobenzoic acid in the synthesis of folic acid.

*Formulations*
Sulfadoxine is used in a fixed-dose combination of 20 parts sulfadoxine with 1 part pyrimethamine and may be administered orally or by the intramuscular route:

- Tablets containing 500 mg of sulfadoxine and 25 mg of pyrimethamine.
- Ampoules containing 500 mg of sulfadoxine and 25 mg of pyrimethamine in 2.5 ml of injectable solution for intramuscular use.

*Pharmacokinetics*
Sulfadoxine is readily absorbed from the gastrointestinal tract. Peak blood concentrations occur about 4 h after an oral dose. The terminal elimination half-life is 4–9 days. Around 90–95% is bound to plasma proteins. It is widely distributed to body tissues and fluids, passes into the fetal circulation and is detectable in breast milk. The drug is excreted in urine, primarily unchanged.

*Toxicity*
Sulfadoxine shares the adverse effect profile of other sulfonamides, although allergic reactions can be severe because of its slow elimination. Nausea, vomiting, anorexia and diarrhoea may occur. Crystalluria causing lumbar pain, haematuria and oliguria is rare compared with more rapidly eliminated sulphonamides. Hypersensitivity reactions may effect different organ system. Cutaneous manifestations can be severe and include pruritus, photosensitivity reactions, exfoliative dermatitis, erythema nodosum, toxic epidermal necrolysis and Stevens-Johnson syndrome[1]. Treatment with sulfadoxine should be stopped in any patient developing a rash because of the risk of severe allergic reactions[2]. Hypersensitivity to sulfadoxine may also cause interstitial nephritis, lumbar pain, haematuria and oliguria. This is due to crystal formation in the

---

[1] Miller KD et al. Severe cutaneous reactions among American travelers using pyrimethamine- sulfadoxine (Fansidar) for malaria prophylaxis. *American Journal of Tropical Medicine and Hygiene*, 1986, 35:451–458.
[2] Bjorkman A, Phillips-Howard PA. Adverse reactions to sulfa drugs: implications for malaria chemotherapy. *Bulletin of the World Health Organization*, 1991, 69:297–304.

urine (crystalluria) and may be avoided by keeping the patient well hydrated to maintain a high urine output. Alkalinization of the urine will also make the crystals more soluble. Blood disorders that have been reported include agranulocytosis, aplastic anaemia, thrombocytopenia, leukopenia and hypoprothrombinaemia. Acute haemolytic anaemia is a rare complication, which may be antibody mediated or associated with glucose-6-phosphate dehydrogenase (G6PD) deficiency. Other adverse effects, which may be manifestations of a generalized hypersensitivity reaction include fever, interstitial nephritis, a syndrome resembling serum sickness, hepatitis, myocarditis, pulmonary eosinophilia, fibrosing alveolitis, peripheral neuropathy and systemic vasculitis, including polyarteritis nodosa. Anaphylaxis has been reported only rarely. Other adverse reactions that have been reported include hypoglycaemia, jaundice in neonates, aseptic meningitis, drowsiness, fatigue, headache, ataxia, dizziness, drowsiness, convulsions, neuropathies, psychosis and pseudomembranous colitis.

**Pyrimethamine**

Molecular weight: 248.7

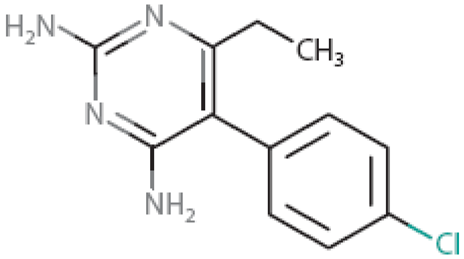


Pyrimethamine is a diaminopyrimidine used in combination with a sulfonamide, usually sulfadoxine or dapsone. It exerts its antimalarial activity by inhibiting plasmodial dihydrofolate reductase thus indirectly blocking the synthesis of nucleic acids in the malaria parasite. It is a slow-acting blood schizontocide and is also possibly active against pre-erythrocytic forms of the malaria parasite and inhibits sporozoite development in the mosquito vector. It is effective against all four human malarials, although resistance has emerged rapidly. Pyrimethamine is also used in the treatment of toxoplasmosis, and isosporiasis and as prophylaxis against Pneumocystis carinii pneumonia. Pyrimethamine is no longer used alone as an antimalarial, only in synergistic combination with slowly eliminated sulfonamides for treatment (sulfadoxine, sulfalene) or with dapsone for prophylaxis.

*Formulations*
Pyrimethamine is currently used mainly in a fixed-dose combination with slowly eliminated sulfonamides, either of 20 parts sulfadoxine with 1 part pyrimethamine for which there are oral and parenteral formulations:

- Tablets containing 500 mg of sulfadoxine and 25 mg of pyrimethamine.
- Ampoules containing 500 mg of sulfadoxine and 25 mg of pyrimethamine in 2.5 ml of injectable solution for intramuscular use.

*Pharmacokinetics*
Pyrimethamine is almost completely absorbed from the gastrointestinal tract and peak plasma concentrations occur 2–6 h after an oral dose. It is mainly concentrated in the kidneys, lungs, liver and spleen, and about 80–90% is bound to plasma proteins. It is metabolized in the liver and slowly excreted via the kidneys. The plasma half-life is around 4 days. Pyrimethamine crosses the blood-brain barrier and the placenta and is detectable in breast milk. Absorption of

the intramuscular preparation is incomplete and insufficiently reliable for this formulation to be recommended[3].

*Toxicity*
Pyrimethamine is generally very well tolerated. Administration for prolonged periods may cause depression of haematopoiesis due to interference with folic acid metabolism. Skin rashes and hypersensitivity reactions also occur. Larger doses may cause gastrointestinal symptoms such as atrophic glossitis, abdominal pain and vomiting, haematological effects including megaloblastic anaemia, leukopenia, thrombocytopenia and pancytopenia, and central nervous system effects such as headache and dizziness. Acute overdosage of pyrimethamine can cause gastrointestinal effects and stimulation of the central nervous system with vomiting, excitability and convulsions. Tachycardia, respiratory depression, circulatory collapse and death may follow. Treatment of overdosage is supportive.

*Drug interactions*
Administration of pyrimethamine with other folate antagonists such as cotrimoxazole, trimethoprim, methotrexate or with phenytoin may exacerbate bone marrow depression. Given with some benzodiazepines, there is a risk of hepatotoxicity.

## 2. **Mefloquine**

Molecular weight: 378.3

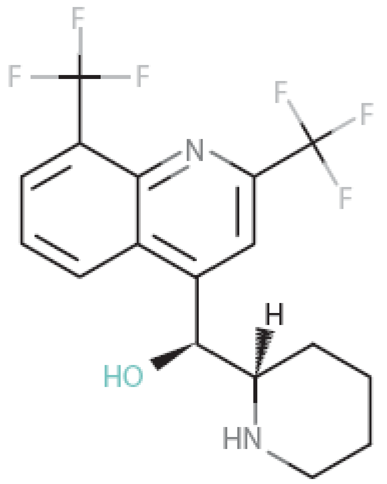


Mefloquine is a 4-methanolquinoline and is related to quinine. It is soluble in alcohol but only very slightly soluble in water. It should be protected from light. The drug is effective against all forms of malaria.

*Formulations*
Mefloquine is administered by mouth as the hydrochloride salt (250 mg base equivalent to 274 mg hydrochloride salt):
• Tablets containing either 250 mg salt (United States of America) or 250 mg base (elsewhere).

*Pharmacokinetics*
Mefloquine is reasonably well absorbed from the gastrointestinal tract but there is marked interindividual variation in the time required to achieve peak plasma concentrations. Splitting

3 Winstanley PA et al. The disposition of oral and intramuscular pyrimethamine/sulphadoxine in Kenyan children with high parasitaemia but clinically non-severe falciparum malaria. *British Journal of Clinical Pharmacology*, 1992, 33:143–148.

the 25 mg/kg dose into two parts given at an interval of 6–24 h augments absorption and improves tolerability[4] . Mefloquine undergoes enterohepatic recycling. It is approximately 98% bound to plasma proteins and is widely distributed throughout the body. The pharmacokinetics of mefloquine may be altered by malaria infection with reduced absorption and accelerated clearance[5, 6]. When administered with artesunate, blood concentrations are increased, probably as an indirect effect of increased absorption resulting from more rapid resolution of symptoms[4]. Mefloquine is excreted in small amounts in breast milk. It has a long elimination half-life of around 21 days, which is shortened in malaria to about 14 days, possibly because of interrupted enterohepatic cycling[7,8,9]. Mefloquine is metabolized in the liver and excreted mainly in the bile and faeces. Its pharmacokinetics show enantioselectivity after administration of the racemic mixture, with higher peak plasma concentrations and area under the curve values, and lower volume of distribution and total clearance of the SR enantiomer than its RS antipode[10,11,12].

*Toxicity*

Minor adverse effects are common following mefloquine treatment, most frequently nausea, vomiting, abdominal pain, anorexia, diarrhoea, headache, dizziness, loss of balance, dysphoria, somnolence and sleep disorders, notably insomnia and abnormal dreams. Neuropsychiatric disturbances (seizures, encephalopathy, psychosis) occur in approximately 1 in 10 000 travellers receiving mefloquine prophylaxis, 1 in 1000 patients treated in Asia, 1 in 200 patients treated in Africa, and 1 in 20 patients following severe malaria[13,14,15,16]. Other side effects reported rarely include skin rashes, pruritus and urticaria, hair loss, muscle weakness, liver function disturbances and very rarely thrombocytopenia and leukopenia. Cardiovascular effects have included postural hypotension, bradycardia and, rarely, hypertension, tachycardia or palpitations and minor changes in the electrocardiogram. Fatalities have not been reported following overdosage, although cardiac, hepatic and neurological symptoms may be seen. Mefloquine should not be given with halofantrine because it exacerbates QT prolongation. There is no evidence of an adverse interaction with quinine[17].

*Drug interactions*

---

[4] Price R et al. Pharmacokinetics of mefloquine combined with artesunate in children with acute falciparum malaria. *Antimicrobial Agents and Chemotherapy*, 1999, 43:341–346.

[5] Krishna S, White NJ. Pharmacokinetics of quinine, chloroquine and amodiaquine. Clinical implications. *Clinical Pharmacokinetics*, 1996, 30:263–299

[6] Simpson JA et al. Population pharmacokinetics of mefloquine in patients with acute falciparum malaria. *Clinical Pharmacology and Therapeutics*, 1999, 66:472–484.

[7] Slutsker LM et al. Mefloquine therapy for *Plasmodium falciparum* malaria in children under 5 years of age in Malawi: in vivo/in vitro efficacy and correlation of drug concentration with parasitological outcome. *Bulletin of the World Health Organization*, 1990, 68:53–59.

[8] Karbwang J et al. Pharmacokinetics and pharmacodynamics of mefloquine in Thai patients with acute falciparum malaria. *Bulletin of the World Health Organization*, 1991, 69:207–212.

[9] Nosten F et al. Mefloquine pharmacokinetics and resistance in children with acute falciparum malaria. *British Journal of Clinical Pharmacology*, 1991, 31:556–559.

[10] Svensson US et al. Population pharmacokinetic and pharmacodynamic modelling of artemisinin and mefloquine enantiomers in patients with falciparum malaria. *European Journal of Clinical Pharmacology*, 2002, 58:339–351.

[11] Gimenez F et al. Stereoselective pharmacokinetics of mefloquine in healthy Caucasians after multiple doses. *Journal of Pharmaceutical Sciences*, 1994, 83:824–827.

[12] Bourahla A et al. Stereoselective pharmacokinetics of mefloquine in young children. *uropean Journal of Clinical Pharmacology*, 1996, 50:241–244.

[13] Bem JL, Kerr L, Stuerchler D. Mefloquine prophylaxis: an overview of spontaneous reports of severe psychiatric reactions and convulsions. *Journal ofTropical Medicine and Hygiene*, 1992, 95:167–179.

[14] ter Kuile FO et al. Mefloquine treatment of acute falciparum malaria: aprospective study of non-serious adverse effects in 3673 patients. *Bulletin of theWorld Health Organization*, 1995, 73:631–642.

[15] Phillips-Howard PA, ter Kuile FO. CNS adverse events associated with antimalarial agents. Fact or fiction? *Drug Safety*, 1995, 12:370–383.

[16] Mai NTH et al. Post-malaria neurological syndrome. *Lancet*, 1996, 348:917–921.

[17] Supanaranond W et al. Lack of a significant adverse cardiovascular effect of combined quinine and mefloquine therapy for uncomplicated malaria. *Transactions of the Royal Society of Tropical Medicine and Hygiene*, 1997, 91:694–696.

There is a possible increase in the risk of arrhythmias if mefloquine is given together with beta blockers, calcium channel blockers, amiodarone, pimozide, digoxin or antidepressants; there is also a possible increase in the risk of convulsions with chloroquine and quinine. Mefloquine concentrations are increased when given with ampicillin, tetracycline and metoclopramide.
Caution should be observed with alcohol.

## 3. Cotrimoxazole

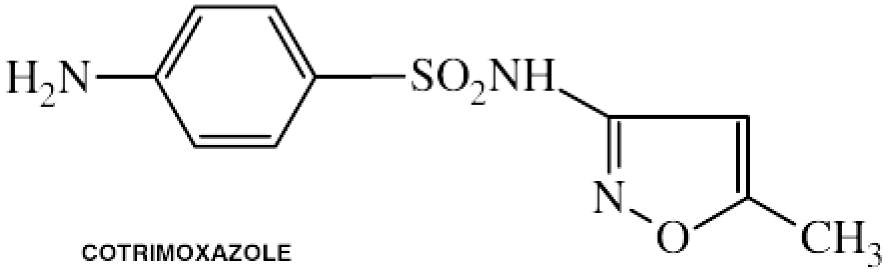


Cotrimoxazole (CTX), is a fixed combination of Sulfamethoxazole-Trimethoprim (SMX-TMP). CTX is a broadspectrum antimicrobial agent that targets a variety of aerobic Gram-positive and Gram-negative organisms and protozoa.

*Formulations*:
The drug is widely available in both syrup and solid formulations at low cost in most places, including resource-limited settings. CTX contains 400 mg of SMX and 80 mg of TMP.

Provision of CTX as primary or secondary prophylaxis for prevention of *Pneumocystis jiroveci* pneumonia (PCP) (formerly *Pneumocystis carinii* pneumonia) and toxoplasmosis has been part of the standard care in the management of HIV-infected individuals in developed countries since the early 1990s.

*Adverse Reactions:*
The potential adverse reactions to CTX include skin rash, haematological reactions, namely neutropaenia anaemia and liver toxicity. The available studies and data from programmes in lowand middle-income settings report low rates of adverse reactions. There was some suggestion that additional haematological adverse effects could be detected with availability of laboratory monitoring. Studies from resource-poor settings have, to date, documented fewer side effects in adults and in children as well.

The most commonly observed adverse reaction among 540 children enrolled in the CHAP study was mild, moderate rash, with no report of serious or life-threatening rash (grade 3 or 4)[18].

[18] Chintu C et al. Cotrimoxazole as prophylaxis against opportunistic infections as HIV-infected Zambian children (CHAP): a double-blind randomised placebo-controlled trial. *Lancet*, 2004, 364:1865-71.

# Appendix 2. Informed Consent form (Trial 1)

***Informed Consent / Assent / Guardian Consent for Enrollment***

**This form covers the woman and infant. It must be signed by the woman participating in the study (and in some countries by a legal guardian according to national policies).**

---

**Flesch-Kincaid grade level = 8.3**

**Title of the study**
Comparison of safety, tolerability and efficacy of mefloquine (MQ) to sulfadoxine-pyrimethamine (SP) as intermittent preventive treatment in pregnancy (IPTp) in the context of Insecticide Treated Nets (ITNs).

**Introduction**
The Barcelona Centre for International Health Research (CRESIB) in Spain is coordinating a study to evaluate new drugs to prevent malaria in pregnant women. The study will be carried out in 4 African countries. The study is coordinated by Professor Clara Menendez from CRESIB.

The study will be testing if a drug called mefloquine can prevent pregnant women from malaria while they are using treated mosquito nets. As you know, sometimes you may have malaria without feeling sick. Malaria may be hidden in the placenta and can cause baby to be born small and weak, even if you never feel sick. The baby may also be born too early.

Right now pregnant women get at least two doses of medicine called Sulfadoxine-Pyrimethamine (SP) to protect them from malaria. However, in some African countries these 2 doses of **SP** no longer work as well as they once did. That is why it is necessary to look for other anti-malaria drugs to prevent malaria in pregnant women.

Of all the current antimalarial drugs for pregnant women, mefloquine is the most promising. One of the reasons is that it can be given as a single dose, which makes it easy to take.

You are being asked to participate because the initial screening makes you eligible to join the study. Before you decide if you wish to be in this study, you will be informed about the study and about things that you will be asked to do if you agree to join.

**Purpose of the study and study groups**
The information coming from this study will help to prevent malaria in pregnant African women. The trial will compare mefloquine (MQ) to **SP** as prevention for malaria in pregnancy together with use of insecticide treated nets. There will be **4716** pregnant women from Mozambique, Tanzania, Benin, and Gabon enrolled in this study.

Some women in the study will be receiving mefloquine **as a full dose, other mefloquine as a split dose in 2 days and a third group will receive SP.** Neither the study team nor you can pick the study group as this could affect the study results. You will be put into one of the 3 groups by chance.

Participants from **the 3** groups will have the same study visits. Before you learn about the study, it is important that you know that your participation in this study is voluntary and you may decide not to participate, not to have the tests, or to withdraw from the study at any time.

**What happens during the study**

If you agree to be in this study, your first visit will continue today, after you read, discuss, and sign or put thumbprint on this form. You will be asked to come back to the clinic for a minimum of two visits before delivery. In addition, you must agree to deliver your baby at the study facility rather than at home.

If you agree to be in this study:

- We will first ask you some questions about yourself and your health.
- We will ask you to give information on where you live and how to keep in contact with you. The study staff will use this information to visit you at home to see how you are feeling and to remind you about your study visits.
- A study clinician will examine you and will check your pregnancy status
- You will also be asked to give a teaspoon of blood by finger prick at the first visit for tests of your blood
- At the first study visit at the clinic, in the presence of the study nurse, you will take either **MQ (full dose or split dose)** or **SP** (assigned by chance)
- The second dose of MQ or **SP** will be given to you at the next ANC visit at least one month apart from the previous dose.
- In case you will be unwell with malaria or other infection, you will have additional blood tests done and you will be given medicine, if needed, and asked to come back here as scheduled by study staff
- At enrolment you will receive a long lasting insecticide treated net and will be told how to use it
- You and your baby will receive a unique identification number (ID) and identification study card, which you will be requested to present to the study staff at every visit
- You will also be asked to give us a permission to take a photo of you and your baby after your baby is born so we can easy identify you and your baby at your clinic visits
- Even though you will receive drug for malaria prevention it is possible that you may still get sick. Therefore you will be asked to come to the clinic whenever you feel unwell, get fever or any other symptoms.
- At delivery you will be visited in the labor ward and you and your newborn baby will be examined by the study personnel
- In addition to venous blood being collected from you, also a sample of cord blood will be taken to be tested for malaria
- Your placenta will be examine at the study laboratory and also tested for malaria
- Blood sample will be taken from your baby for malaria tests
- You must agree to deliver at the health facility but in case you deliver at home, the study staff will visit you as soon as possible but not later than one week after delivery and will ask you questions about your delivery and about health of your infant. At this visit you and your infant will be examined by the study personnel. Blood sample will be taken from you and your baby for tests of malaria
- You will be asked to come back with your newborn to the study clinic around 1 month of delivery. If you agree, at that time a picture of you with your baby will be taken.
- You will return with your baby to the clinic two more times within a year after delivery
- During these visits we will exam your baby to know how mature she/he is
- If your child has signs of malaria, blood will be taken for tests and appropriate treatment given
- Study staff will visit you at home a few times after delivery to exam your baby

**Unscheduled visits**

- Throughout the **first year** of your baby's life she/he will be attended by the study staff when you bring her/him to the clinic. During those visits study nurses will exam your child and only if necessary take a sample of blood and provide treatment.
- You can come to the clinic at any time during this study. If you feel discomfort or are in pain, you should call the study staff or come to the clinic.

- You can also ask any questions at any point during the study, even during time other than your schedule visit

**Alternatives to joining the study**
If you choose not to participate in this study or to leave the study after enrollment you are encouraged to come to this ANC for your routine visits and for any questions or concerns you may have related to your pregnancy. You will receive standard ANC care as before. We will refer you to another doctor if necessary.

**Risks or discomforts** (mother and infant)
*Risks from blood draws*
You will feel slight pain when we take blood from your finger or vein and your baby will feel slight pain if we take blood from the baby's heel. There will be no other risks to your newborn baby. Sometimes you may feel little dizzy or your head may feel light. There may be a small swelling of the skin where the needle went in. Those will go away in short time and the study personnel will examine you and your baby for those symptoms.
*Risks from study drugs*
Mefloquine is well tolerated when used to prevent malaria. Sometimes side effects are: dizziness, gastrointestinal symptoms, bad dreams, and difficulty sleeping.

**Benefits to you and your infant**
By participating in the study, you may get better diagnosis of malaria because of increased number of tests for malaria. You and your baby will be regularly seen by clinical staff and in case of any symptoms or abnormal test results you and your baby will be either treated here or referred to another clinic for medical care.

**Reasons for taking you out of the study without your consent**
You may be removed from the study without your consent for the following reasons:

- You are found to not be eligible for the study
- The research study is stopped or canceled
- The study staff feels that staying in the study would be harmful to you
- You are not able to attend visits or complete the study tests
- Other administrative reasons that will be made clear to you

**Costs to you**
There is no cost to you for participating in the study. Treatments available to you from the study for malaria will be given to you free of charge.

**Your records will be private**
Efforts will be made to keep your personal information as confidential as it is possible and allowed by the law. You and your baby will be identified by a study participant ID number and personal information from your and your baby records will not be released without your written permission. You and your baby will not be personally identified in any publication about this study. Your records may be reviewed by: study monitors, study staff, study auditors, sponsors, and Ethics Committees.

To ensure your medical safety, study staff may verify, from time to time, that you are not enrolled in any other research studies. In addition, if needed, we will request permission to access non-study medical records related to any of your illness. All copies of your records will be kept in a locked file cabinet. Only study staff will have access to this cabinet.

**New findings**
You will be told of any new information learned during the study that might cause you to change your mind about staying in the study.

**Injury because of being in the study**
Based upon what we know, it is unlikely that you will be injured as a result of being in this study. It is important that you tell the study staff if you feel that you have been injured because of taking part in this study. If you are injured as a result of being in this study, the study clinic will give you immediate necessary treatment for your injuries. The cost of this treatment will not be charged to you. You will then be told where you may receive additional treatment for injuries.

When you sign this consent form, you do not give up your rights to care for injury caused by being in the study.

**Contact information**
You will be given a copy of this form to take with you.
If you ever have questions about this study or in case you are injured as a result of participation in this study, you should contact: Principal Investigator, ***[each site will provide name and phone number of their site PI]*** or Project Coordinator, Professor Clara Menendez at phone number: 3493 2275400

**Your rights as a study participant**
This research has been reviewed and approved by the Ethics Committee of the Hospital Clinic of Barcelona, Spain and the local IRB in your country. These committees have reviewed this study in order to help protect participants.

**Photograph**
We will ask to take your and your baby's picture. This will help us make sure that no one else can get the study care that is meant for you. It will also help us find you in case of emergency. This picture will not have your name on it, only your study identification number. It will be kept in a secure place with your records. At the end of the study the picture will be given to you if you want it, or it will be destroyed. If you do not want your picture taken, we will not take it. If you chose not to have your picture taken, it will not affect your ability to take part in the study in any way.

- ☐ I give consent to have my picture taken
- ☐ I DO NOT give consent to have my picture taken

**Storage of blood samples**
While you are in this study there may be some blood samples taken from you that might be useful for future research. You are being asked if you would agree to the storage of these samples. If you agree to the storage of your samples there will be no additional samples taken from you for storage. After all the tests are done for this research study, there may be some left over samples. If you agree, left over samples will be kept and used for future research. If you allow us to store your samples, you may change your mind at any time while the study is ongoing. Any samples that are not stored or that are removed from storage will be destroyed.

Permission statement: sign or thumbprint on the appropriate line. [*If participant does not permit storage of blood, this box should not be signed.*]

- ☐ I give consent to have my specimens to be stored for further research

  ____________________

*Volunteer's Signature or Thumbprint*

(if cannot write)

☐ I **DO NOT** give consent to have my specimens stored for further research

**If you accept to participate in this study please answer to the following questions:**

1. What kind of treatments can you receive during the study?
2. In addition to the dugs, what else will you receive to prevent malaria?
3. Can you recall how many times we will ask you to give a blood sample? And how many times your child will be finger-pricked?
4. Can you recall how many times in the presence of study nurse you will take mefloquine or placebo to prevent malaria?
5. When do you have to bring your child to the study clinic after birth?
6. Can you get malaria even if you participate in the study? And your baby?
7. Where do you have to go if you or baby has fever?

**STATEMENT of CONSENT AND SIGNATURE**

Participant and newborn approval:

The consent form has been explained to me and I agree to take part in this study. **I also agree to let my newborn baby take part in this study.** I understand that I am free to choose to be in the study and that saying "No" will not affect the treatment I get in this clinic, now and in future.

**NOTE:** You are not giving up any of your legal rights by signing this informed consent document.

If you agree circle YES

| | | |
|---|---|---|
| **Volunteer's Name** *(print)* | ***Volunteer's Signature or Thumbprint*** (if cannot write) | ***Date*** |

| | | |
|---|---|---|
| **Volunteer's Legal Guardian or Representative** (as per country policy) *(print)* | ***Legal Guardian's Signature*** | ***Date*** |

| | | |
|---|---|---|
| **Witness's Name** (if participant illiterate) *(print)* | ***Witness's Signature*** | ***Date*** |

I have explained the purpose of this study to the volunteer. To the best of my knowledge, she understands the purpose, procedures, risks and benefits of this study.

| | | |
|---|---|---|
| **Investigator/Designee Name** *(print)* | ***Investigator/Designee Signature*** | **Date** |

**NOTE:** This consent form with original signatures must be retained on file by the principal investigator. A copy must be given to the volunteer.

If the woman refuses to take her copy of the consent with her, she states so below and signs and dates her decline statement.

## Appendix 3. National Guidelines for Malaria Treatment in Pregnant women

| Country | Site | 1st Trimester Uncomplicated Malaria | 2nd 3rd Trimester Uncomplicated Malaria | Severe Malaria |
|---|---|---|---|---|
| Benin | Allada | Quinine | Artemether- Lumefantrine (AL) | Quinine |
| Gabon | Lambaréné | Quinine | ACT (AL or AS+Amodiaquine) | Quinine |
| Kenya | Kisumu | Quinine | Artemether- Lumefantrine (AL) | Quinine |
| Mozambique | Manhiça | Quinine | Quinine | Quinine |
| Tanzania | Ifakara | Quinine | Artemether-Lumefantrine (AL) | Quinine |

# Appendix 4. Ballard Examination

**Birth order (please circle):** First-born Second- born

**NEUROMUSCULAR MATURITY**

| NEUROMUSCULAR MATURITY SIGN | SCORE | | | | | | | RECORD SCORE HERE |
|---|---|---|---|---|---|---|---|---|
| | -1 | 0 | 1 | 2 | 3 | 4 | 5 | |
| POSTURE | 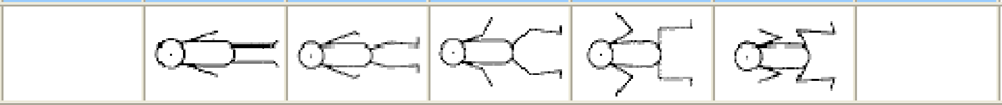 | | | | | | | |
| SQUARE WINDOW (Wrist) | 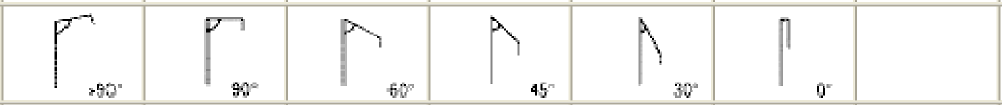  | | | | | | | |
| ARM RECOIL | 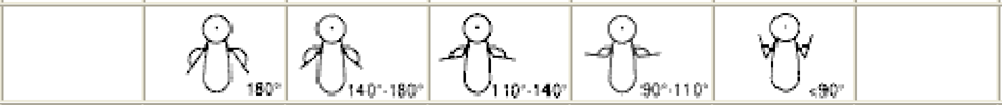  | | | | | | | |
| POPLITEAL ANGLE | 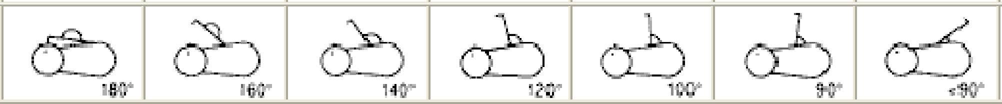  | | | | | | | |
| SCARF SIGN | 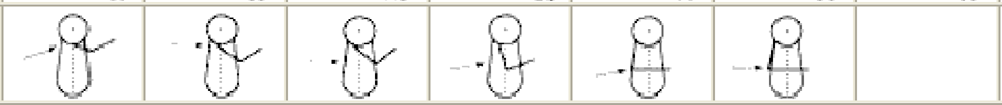 | | | | | | | |
| HEEL TO EAR | 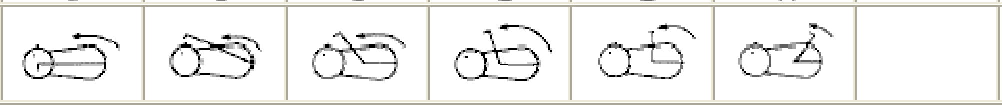 | | | | | | | |
| TOTAL NEUROMUSCULAR MATURITY SCORE | | | | | | | | |

**PHYSICAL MATURITY**

| PHYSICAL MATURITY SIGN | SCORE | | | | | | | RECORD SCORE HERE |
|---|---|---|---|---|---|---|---|---|
| | -1 | 0 | 1 | 2 | 3 | 4 | 5 | |
| SKIN | sticky, friable, transparent | gelatinous, red, translucent | smooth pink, visible veins | superficial peeling &/or rash, few veins | cracking, pale areas, rare veins | parchment, deep cracking, no vessels | leathery, cracked, wrinkled | |
| LANUGO | None | Sparse | Abundant | Thinning | bald areas | mostly bald | | |
| PLANTAR SURFACE | heel-toe 40-50 mm: -1 <40 mm: -2 | >50 mm no crease | faint red marks | anterior transverse crease only | creases ant. 2/3 | creases over entire sole | | |
| BREAST | Imperceptible | barely perceptible | flat areola no bud | stippled areola 1-2 mm bud | raised areola 3-4 mm bud | full areola 5-10 mm bud | | |
| EYE / EAR | lids fused loosely: -1 tightly: -2 | lids open pinna flat stays folded | sl. curved pinna; soft; slow recoil | well-curved pinna; soft but ready recoil | formed & firm instant recoil | thick cartilage ear stiff | | |
| GENITALS (Male) | scrotum flat, smooth | scrotum empty, faint rugae | testes in upper canal, rare rugae | testes descending, few rugae | testes down, good rugae | testes pendulous, deep rugae | | |
| GENITALS (Female) | clitoris prominent & labia flat | prominent clitoris & small labia minora | prominent clitoris & enlarging minora | majora & minora equally prominent | majora large, minora small | majora cover clitoris & minora | | |
| TOTAL PHYSICAL MATURITY SCORE | | | | | | | | |

# Appendix 5. Psychomotor Development test

## 1 Newborn: assessments at birth

a) Figure 1

| | Expected findings include: |
|---|---|
| Gross motor skills | Symmetric and anti-gravitational movements of the 4 extremities (figures 2 and 3). Normal muscle tone |
| Language | Crying |
| Social skills | Response to pinch |
| Audition | Quiet with the voice; frighten by great noises |
| Vision | Looks at the face; responses to light |

b) Figure 2

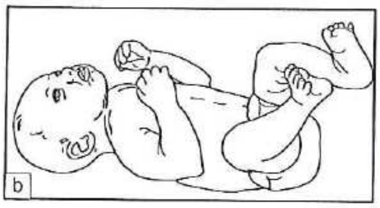


Dorsal decubitus:
Symmetric position, bended extremities

c) Figure 3

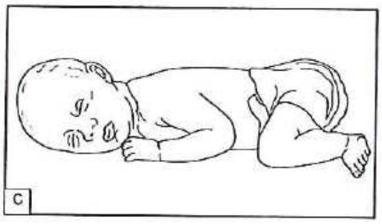


Ventral decubitus:
Flexion position, bended knees

## 2 Infants 6-8 weeks of age

a) Figure 1

| | Expected findings include: |
|---|---|
| Gross motor skills | Symmetric movements of the 4 extremities (figures 2 and 3). Normal muscle tone |
| Fine motor skills | Eyes follow objects (6 weeks) (figure 4); conjugated movement is observed; no strabismus or nistagmus |
| Language/Audition | Normal crying; responses to sounds |
| Social skills | Response to smiles (figure 4) |
| Physical examination | Weight, height, to rule out congenital malformations |

b) Figure 2

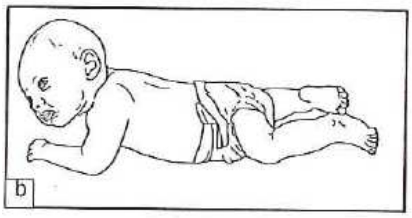


Ventral decubitus:
The head stands at 45º

c) Figure 3

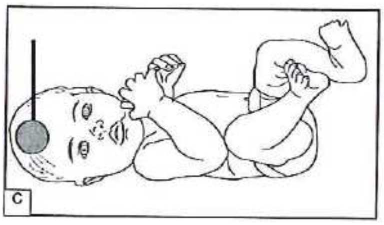


Dorsal decubitus:
Follows an object in movement turning the head

d) Figure 4

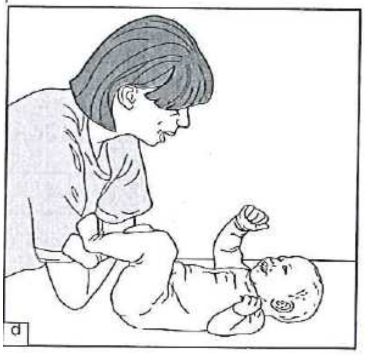


Smiles at some stimuli

## 3 Assessments at 6-9 months of age

a) Figure 1

| | Expected findings include: |
|---|---|
| Gross motor skills | Seats without leaning (six months) (figure 2)<br>Can stand up (seven months)<br>Tractions to stand up (nine months) |
| Fine motor skills /vision | Tries to catch small objects, holds them by palm grasp (figure 3) and passes them from one hand to the other (six months)<br>Can bang objects holding them in different hands<br>No strabismus |
| Language/Audition | Turns at someone's voice (seven months)<br>Babbles ; says "daddy", "mammy" (ten months) at random |
| Social skills | Can bring solid food to his/her mouth (six months) |
| Physical examination | Weight, height |

b) Figure 2

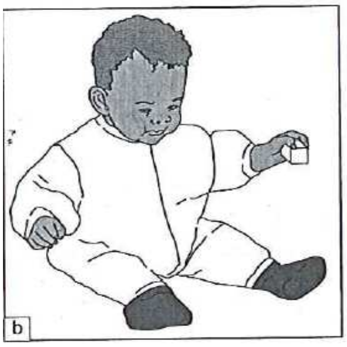


Seats without leaning

c) Figure 3

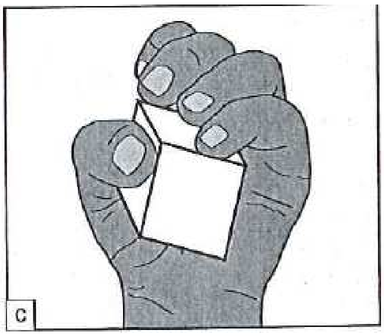


Palm grasp

## 4 Assessments at 12 months of age

a) Figure 1

| | Expected findings include: |
|---|---|
| Gross motor skills | Walks leaning on furniture (nine months)<br>Can walk with help (figure 2) and does some steps without leaning (twelve months) |
| Fine motor skills /vision | Efficacious pincer grasping between the thumb and forefinger (figure 3) of small objects (10,5 months)<br>No observed strabismus, neither according to parents |
| Language/Audition | Understands orders like “no”, “bring”; correctly says “daddy”, “mammy” and a few more words (thirteeen months) |
| Social skills | Drinks from a glass (twelve months)<br>Eats with his/her hands or with a spoon<br>Says “goodbye” with his/her hand<br>Social response: is scared of strangers |

b) Figure 2

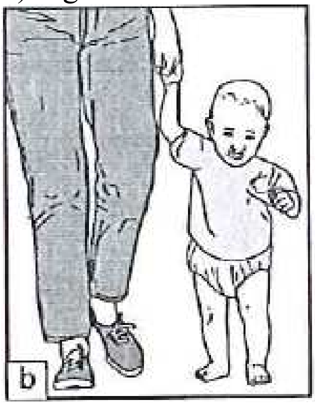


Walks with help

c) Figure 3

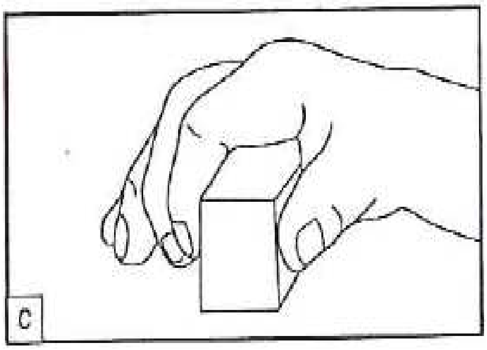


Pincer grasp

………………………………………………

*Adapted from Lissauer T. Anamnesis and physical examination. In: Lissauer T, Clayden G. Illustrated textbook of paediatrics. First edition. Mosby-Year Book. 1999.*

## Appendix 6. National Guidelines of Malaria treatment for Infants

| Country | Site | Uncomplicated Malaria | Severe Malaria |
|---|---|---|---|
| Benin | Allada | ACT | Quinine |
| Gabon | Lambaréné | ACT<br>(AL or AS+ Amodiaquine) | Quinine |
| Kenya | Kisumu | <5kg→ Quinine<br>≥5kg → AL | Quinine |
| Mozambique | Manhiça | < 5g → Quinine<br>≥ 5 kg:<br>1st line: SP + AS<br>2nd line: AL<br>3rd line: Quinine | Quinine |
| Tanzania | Ifakara | <5kg→ Quinine<br>≥5kg → AL | Quinine |

# Appendix 7. Informed Consent Form (Trial 2)

*Informed Consent / Assent / Guardian Consent for Enrollment*

**This form covers the woman and infant. It must be signed by the woman participating in the study (and in some countries by a legal guardian according to national policies).**

---

**Flesch-Kincaid grade level = 8.3**

**Title of the study**
Evaluation of the safety and efficacy of mefloquine (MQ) as intermittent preventive treatment in pregnancy (IPTp) in women receiving cotrimoxazole (Septrin®) prophylaxis and in the context of Insecticide Treated Nets (ITNs).

**Introduction**
The Barcelona Centre for International Health Research (CRESIB) in Spain is coordinating a study to evaluate new drugs to prevent malaria in pregnant women. The study will be carried out in 3 African countries. The study is coordinated by Professor Clara Menendez from CRESIB.

The study will be testing if a drug called mefloquine can prevent pregnant women receiving Septrin® (cotrimoxazole) from malaria while they are using treated mosquito nets. As you know, sometimes you may have malaria without feeling sick. Malaria may be hidden in the placenta and can cause baby to be born small and weak, even if you never feel sick. The baby may also be born too early. If the woman is infected with HIV, this increases even more her chances of getting malaria and makes it difficult to treat.

Right now pregnant women who are HIV positive receive Septrin® to prevent infections but we do not know if Septrin® prevents malaria. Also it is complicated for those women to take anti-malaria drugs because the medicines can have interaction with each other. That is why it is necessary to look for other anti-malaria drugs to prevent malaria in pregnant women.

Of all the current antimalarial drugs for pregnant women, mefloquine is the most promising. One of the reasons is that it can be given as a single dose, which makes it easy to take.

You are being asked to participate because the initial screening makes you eligible to join the study. Before you decide if you wish to be in this study, you will be informed about the study and about things that you will be asked to do if you agree to join.

**Purpose of the study and study groups**
The information coming from this study will help to prevent malaria in African women infected with HIV.

The trial will compare mefloquine (MQ) to placebo (a substance similar to MQ but without any effect) as prevention for malaria in pregnancy together with using Septrin® and insecticide treated mosquito nets. There will be 1070 pregnant women from Mozambique, Kenya, and Tanzania enrolled in this study.

Some women in the study will be receiving mefloquine and other placebo. Also you will be given Septrin® to take with you home and administer one tablet every day to prevent any infection. Neither the study team nor you can pick the study group as this could affect the study results. You will be put into one of the two groups by chance.

Participants from both groups will have the same study visits. Before you learn about the study, it is important that you know that your participation in this study is voluntary and you may decide not to participate, not to have the tests, or to withdraw from the study at any time.
Let me explain to you what we mean by placebo. The placebo is a tablet that looks like mefloquine tablet but it does not have the ingredients that the mefloquine has and it will not prevent against malaria. You will receive either mefloquine or placebo by chance.

**What happens during the study**
If you agree to be in this study, your first visit will continue today, after you read, discuss, and sign or put thumbprint on this form.

You will be asked to come back to the clinic for a minimum of three visits before delivery. In addition, you must agree to deliver your baby at the study facility rather than at home. Because you have HIV virus, you will be offered drugs for the prevention of mother to child transmission of HIV as per routine antenatal care and will be followed up as usual.

If you agree to be in this study:

- We will first ask you some questions about yourself and your health
- We will ask you to give information on where you live and how to keep in contact with you. The study staff will use this information to visit you at home to see how you are feeling and to remind you about your study visits.
- A study clinician will examine you and will check your pregnancy status
- You will also be asked to give a teaspoon of blood by finger prick at the first visit for tests of your blood
- At the first study visit at the clinic, in the presence of the study nurse, you will take either mefloquine or placebo (assigned by chance)
- The second and the third doses of MQ or placebo will be given to you at the next ANC visits at least one month apart
- You will also receive Septrin® to take home and take it once a day as per routine ANC care
- In case you will be unwell with malaria or other infection, you will have additional blood tests done and if needed you will be given medicine and asked to come back here as scheduled by study staff
- At enrolment you will receive a long lasting insecticide treated net and will be told how to use it
- You and your baby will receive a unique identification number (ID) and identification study card, which you will be requested to present to the study staff at every visit
- Even though you will receive drug for malaria prevention (if in mefloquine group), it is possible that you may still get sick. Therefore you will be asked to come to the clinic whenever you feel unwell, get fever or any other symptoms.
- At delivery you will be visited during in the labor ward and you and your newborn baby will be examined by the study personnel.
- In addition to venous blood being collected from you, also a sample of cord blood will be taken to be tested for malaria
- A piece of placenta will be examined at the study laboratory and also tested for malaria
- Blood sample will be taken from your baby for malaria tests
- As per ANC routine you will be instructed to take a drug (ARV) when contractions begin and your infant will also receive HIV preventive treatment right after birth
- You must agree to deliver at the health facility but in case you deliver at home, the study staff will visit you as soon as possible but not later than one week after delivery and will ask you questions about your delivery and about health of your infant. At this visit you and your infant will be examined by the study personnel. Blood sample will be taken from you and your baby for tests of malaria

- You will be asked to come back with your newborn to the study clinic around 1 month and 2 months after delivery. If you agree, at that time a picture of you with your baby will be taken.
- When your baby is born, your child will be followed up until he/she is two months old
- One month after birth, your baby will be tested for HIV
- During these visits we will exam your baby to see if your baby is growing well
- If your child has signs of malaria, blood will be taken for tests and appropriate treatment given
- Study staff will visit you at home a few times after delivery to exam your baby

Unscheduled visits

- Throughout the two months of your baby's life she/he will be attended by the study staff when you bring her/him to the clinic. During those visits study nurses will exam your child and only if necessary take a sample of blood and provide treatment.
- You can come to the clinic at any time during this study. If you feel discomfort or are in pain, you should call the study staff or come to the clinic.
- You can also ask any questions at any point during the study, even during time other than your scheduled visit

**Alternatives to joining the study**
If you choose not to participate in this study or to leave the study after enrollment you are encouraged to come to this ANC for your routine visits and for any questions or concerns you may have related to your pregnancy. You will receive standard ANC care as before. We will refer you to another doctor if necessary.

**Risks or discomforts** (mother and infant)
*Risks from blood draws*
You will feel slight pain when we take blood from your finger or vein and your baby will feel slight pain if we take blood from the baby's heel. There will be no other risks to your newborn baby. Sometimes you may feel little dizzy or your head may feel light. There may be a small swelling of the skin where the needle went in. Those will go away in short time and the study personnel will examine you and your baby for those symptoms.
*Risks from study drugs*
Mefloquine is well tolerated when used to prevent malaria. Sometimes side effects are: dizziness, gastrointestinal symptoms, bad dreams, and difficulty sleeping.

**Benefits to you and your infant**
By participating in the study, you may get better diagnosis of malaria because of increased number of tests for malaria. You and your baby will be regularly seen by clinical staff and in case of any symptoms or abnormal test results you and your baby will be either treated here or referred to another clinic for medical care.

**Reasons for taking you out of the study without your consent**
You may be removed from the study without your consent for the following reasons:

- You are found to not be eligible for the study
- The research study is stopped or canceled
- The study staff feels that staying in the study would be harmful to you
- You are not able to attend visits or complete the study tests
- Other administrative reasons that will be made clear to you

**Costs to you**
There is no cost to you for participating in the study. Treatments available to you from the study for malaria will be given to you free of charge.

**Your records will be private**
Efforts will be made to keep your personal information as confidential as it is possible and allowed by the law. You and your baby will be identified by a study participant ID number and personal information from your and your baby records will not be released without your written permission. You and your baby will not be personally identified in any publication about this study. Your records may be reviewed by: study monitors, study staff, study auditors, sponsors, and Ethics Committees.
To ensure your medical safety, study staff may verify, from time to time, that you are not enrolled in any other research studies. In addition, if needed, we will request permission to access non-study medical records related to any of your illness. All copies of your records will be kept in a locked file cabinet. Only study staff will have access to this cabinet.

**New findings**
You will be told of any new information learned during the study that might cause you to change your mind about staying in the study.

**Injury because of being in the study**
Based upon what we know, it is unlikely that you will be injured as a result of being in this study. It is important that you tell the study staff if you feel that you have been injured because of taking part in this study. If you are injured as a result of being in this study, the study clinic will give you immediate necessary treatment for your injuries. The cost of this treatment will not be charged to you. You will then be told where you may receive additional treatment for injuries.

When you sign this consent form, you do not give up your rights to care for injury caused by being in the study.

**Contact information**
You will be given a copy of this form to take with you.

If you ever have questions about this study or in case you are injured as a result of participation in this study, you should contact: **Principal Investigator**, ***[each site will provide name and phone number of their site PI]*** Project Coordinator, Professor Clara Menendez at phone number: 3493 2275400

**Your rights as a study participant**
This research has been reviewed and approved by the Ethics Committee of the Hospital Clinic of Barcelona, Spain and the local IRB in your country. These committees have reviewed this study in order to help protect participants.

**Photograph**
We will ask to take your and your baby's picture. This will help us make sure that no one else can get the study care that is meant for you. It will also help us find you in case of emergency. This picture will not have your name on it, only your study identification number. It will be kept in a secure place with your records. At the end of the study the picture will be given to you if you want it, or it will be destroyed. If you do not want your picture taken, we will not take it. If you choose not to have your picture taken, it will not affect your ability to take part in the study in any way.

- ☐ I give consent to have my picture taken
- ☐ I DO NOT give consent to have my picture taken

**Storage of blood samples**
While you are in this study there may be some blood samples taken from you that might be useful for future research. You are being asked if you would agree to the storage of these samples. If you agree to the storage of your samples there will be no additional samples taken

from you for storage. After all the tests are done for this research study, there may be some left over samples. If you agree, left over samples will be kept and used for future research. If you allow us to store your samples, you may change your mind at any time while the study is ongoing. Any samples that are not stored or that are removed from storage will be destroyed.

Permission statement: sign or thumbprint on the appropriate line. [*If participant does not permit storage of blood, this box should not be signed.*]

- ☐ I give consent to have my specimens to be stored for further research

____________________

*Volunteer's Signature or Thumbprint*

(If cannot write)

- ☐ I **DO NOT** give consent to have my specimens stored for further research

**If you accept to participate in this study please answer to the following questions:**

1. What kind of treatments can you receive during the study?
2. In addition to the dugs, what else will you receive to prevent malaria?
3. Can you recall how many times we will ask you to give a blood sample? And how many times your child will be finger-pricked?
4. Can you recall how many times in the presence of study nurse you will take mefloquine or placebo to prevent malaria?
5. When do you have to bring your child to the study clinic after birth?
6. Can you get malaria even if you participate in the study? And your baby?
7. Where do you have to go if you or baby has fever?

**STATEMENT of CONSENT AND SIGNATURE**

Participant and newborn approval:

The consent form has been explained to me and I agree to take part in this study. **I also agree to let my newborn baby take part in this study.** I understand that I am free to choose to be in the study and that saying "No" will not affect the treatment I get in this clinic, now and in future.

**NOTE:** You are not giving up any of your legal rights by signing this informed consent document.

If you agree circle YES

| ____________________ | ____________________ | ____________________ |
|---|---|---|
| **Volunteer's Name**<br>*(print)* | ***Volunteer's Signature or Thumbprint*** (if cannot write) | ***Date*** |

| ____________________ | ____________________ | ____________________ |
|---|---|---|
| **Volunteer's Legal Guardian or Representative**<br>(as per country policy)<br>*(print)* | ***Legal Guardian's Signature*** | ***Date*** |

| ____________________ | ____________________ | ____________________ |
|---|---|---|
| **Witness's Name**<br>(if participant illiterate)<br>*(print)* | ***Witness's Signature*** | ***Date*** |

I have explained the purpose of this study to the volunteer. To the best of my knowledge, she understands the purpose, procedures, risks and benefits of this study.

| ____________________ | ____________________ | ____________________ |
|---|---|---|
| **Investigator/Designee Name**<br>*(print)* | ***Investigator/Designee Signature*** | **Date** |

**NOTE:** This consent form with original signatures must be retained on file by the principal investigator. A copy must be given to the volunteer.

If the woman refuses to take her copy of the consent with her, she states so below and signs and dates her decline statement.

## Appendix 8. National polities for prevention of HIV MTCT

| Country | Site | Women | Newborn |
|---|---|---|---|
| Benin | Allada | ARV | ARV |
| Gabon | Lambaréné | ARV | ARV |
| Kenya | Kisumu | • **If CD4≥350→ AZT from week 28 and 3TC at labour**<br>• **If CD4< 350 → HAART** | **AZT until 1 month after birth** |
| Mozambique | Manhiça | • **Zidovudine (AZT) from week 28**<br>• **Nevirapine + Lamivudine (3TC) + AZT at labour**<br>• **3TC+AZT daily after delivery for 7 days** | **AZT+ NVP until 1 month after birth** |
| Tanzania | Ifakara | Zidovudine from week 28<br>Lamivudine and Nevirapine at labour | ARV<br>(AZT+ 3TC+ NVP) |

*AZT: Zidovudine*
*3TC: Lamivudine*

# Appendix 9. SAEs reporting system

The Investigator or designee will assess the relationship of all AEs to the study drug based on the study specific SOPs and his/her clinical judgment. Site staff also will report all AEs that meet serious adverse event (SAE) reporting requirements according to the procedures and time frames set forth below and in the specific SOPs.

Information on all AEs will be included in reports to applicable government and regulatory authorities. Site staff will report information on all AEs and SAEs to their EC in accordance with all applicable regulations and local EC requirements.

Additionally, all serious adverse events that may be associated with the study product and adverse events of special interest will be reviewed by the DSMB, with follow-up through resolution. The DSMB will investigate those events considered serious and unexpected.

Study participants will be provided a 24-hour telephone number and instructed to contact the study clinician to report any AEs they may experience, except for life-threatening events, for which they will be instructed to seek immediate emergency care.

Depending on the severity of the event, the clinician will instruct the participant to present to the study site (for more mild events) or to an emergency room (for more serious events) for immediate evaluation. Where feasible and medically appropriate, participants will be encouraged to seek medical care where the study clinician is based, and to request that the clinician be paged or otherwise contacted upon their arrival. With appropriate permission of the participant, records from all non-study medical providers related to AEs will be obtained and required data elements will be recorded on study case report forms.

All participants reporting an AE will be followed clinically until the AE resolves (returns to baseline) or stabilizes.

*AE/SAE reporting requirements*
All AE/SAEs including local and systemic reactions not meeting the criteria for "serious adverse events" should be:

- recorded on the appropriate adverse event case report form
- followed through resolution by a study clinician
- reviewed by a study clinician

Study site staff will document on the adverse event CRF all AEs reported by or observed in enrolled study participants regardless of severity and presumed relationship to study drug. Information to be collected includes event description, time of onset, Investigator's assessment of severity, relationship to study product, and time of resolution/stabilization of the event. All AEs will be defined as described in the study specific SOP. All AEs will be graded using the toxicity table that will be developed as SOP.

Any medical condition that is present at the time that the patient is screened should be considered as baseline and not reported as an AE. However, if it deteriorates at any time during the study it should be recorded as an AE.

Any AE considered serious by the Principal Investigator or which meets the aforementioned criteria must be submitted on an SAE form to the sponsor (FCRB) using email address that will be provided. The study clinician will complete a Serious Adverse Event Form within the following timelines:

- All deaths and immediately life threatening events, whether related or unrelated, will be recorded on the SAE CRF and sent by email or fax **within 48 hours** of site awareness of the event.

- SAEs other than death, regardless of relationship, will be reported via email or fax by the site **within 72 hours** of becoming aware of the event.

Other supporting documentation of the event may be requested and should be provided as soon as possible. All SAEs will be followed until satisfactory resolution or until the Principal Investigator deems the event to be chronic or the patient to be stable.

If the event is determined to be serious, unanticipated, and the relationship is anything except "unrelated", then the sponsor (**CRESIB**) will report the SAE to the Hospital Clínic Ethics Committee within 2 working days of being notified, and to the Data Safety Monitoring Board (DSMB). Simultaneously the site PI will notify the local IRB. Otherwise, site staff will report information on all AEs and SAEs to their EC in accordance with all applicable regulations and local EC requirements.

# Appendix 10. Definitions

*Severe maternal anaemia by PCV*:
Haematocrit below 21% or Hb below 7g/dl during pregnancy[19].

*Overall anaemia by PCV*: Haematocrit less than 33% or Hb below 11g/dl, in both mother and infant[20].

*Severe anaemia in the infant by PCV*: Haematocrit less than 25%.

*Foetal Anaemia by PCV*: Haematocrit less than 37% in cord blood and/or Htc<42% in newborn peripheral blood.

*Congenital P falciparum infection*: the presence of asexual *P falciparum* parasites of any density in a blood smear or detected by PCR in a filter paper sample in cord or newborn peripheral blood until the 7th day of life.

*P falciparum infection*: the presence of asexual *P falciparum* parasites of any density in a blood smear or detected by PCR in a filter paper sample.

*Placental infection*:
According to the histological evaluation[21]:

Acute infection: parasites present, with absent or minimal pigment deposition within fibrin or cells within fibrin or any amount of fibrin in free macrophages (any amount of pigment on free macrophages)
Chronic infection: presence of parasites and a significant amount of pigment deposition in fibrin or cells within fibrin
Past infection: presence of pigment with absence of parasites.

All cases with parasites (acute and chronic) will be called active infections.

*Low birth weight*: less than 2500g (up to and including 2499g)[22].

*Premature birth*: birth before the beginning of the 37th week[23].

*Fever*: an axillary temperature equal or greater than 37.5ºC.

*Maternal clinical malaria*: *P falciparum* infection plus any signs and/or symptoms suggestive of malaria disease as referred history of fever in the last 24 hours, axillary fever (Tº>=37.5 ºC), pallor, arthromyalgias, headache and history of convulsions.

*Maternal severe malaria*: positive peripheral parasitaemia and at least one of the following signs or symptoms:

- severe anaemia (as defined in definition 1),
- cerebral malaria: Glasgow Coma Scale<15, convulsions, and/or coma,
- hypoglycaemia: glycaemia < 2.6 mmol/L
- respiratory distress: respiratory rate > 40 br/min
- jaundice
- haemoglobinuria: presence of red blood cells on the urine

---

[19] WHO. The prevalence of anaemia in women. 1992. WHO/MCH/MSM.92.2 Geneva.
[20] Fleming. Iron deficiency in the Tropics. Clinics in Haematology.1982;11,2.
[21] Mamudo R. et al, Human Pathology, 2000; 31:85-93.
[22] WHO. Low birthweight. Country, regional and global estimates. WHO. Unicef. 2004
[23] Berkowitz GS, Epidemiologic Reviews. 1993; 15: 414-43.

- vomiting
- high parasitaemia will be considered > or = 20.000 parasites/nlitre

*Maternal non Severe malaria*: a *P falciparum* infection episode without any of the clinical criteria described in the above definition

*According to protocol (ATP)* - all women who fulfill all the inclusion-exclusion criteria and took the two IPT doses, received the LLITN and from whom data is available for the analysis.

*Intention to treat analysis (ITT)*: the analysis includes all randomized women.

*Not infected placenta*: no evidence of parasites or pigment can be identified by histological assessment

MUAC: Mid-upper arm circumference. Normal range: plus than 22 cm. Indirect index of malnutrition in adults.

RPR: Rapid Plasma Reagin test; non treponemal test for syphilis infection.

# Appendix 11. World Medical Declaration of Helsinki

**WORLD MEDICAL ASSOCIATION DECLARATION OF HELSINKI**
**Ethical Principles for Medical Research Involving Human Subjects**
Adopted by the 18th WMA General Assembly, Helsinki, Finland, June 1964, and amended by the:
29th WMA General Assembly, Tokyo, Japan, October 1975
35th WMA General Assembly, Venice, Italy, October 1983
41st WMA General Assembly, Hong Kong, September 1989
48th WMA General Assembly, Somerset West, Republic of South Africa, October 1996
52nd WMA General Assembly, Edinburgh, Scotland, October 2000
53th WMA General Assembly, Washington 2002 (Note of Clarification on paragraph 29 added)
55th WMA General Assembly, Tokyo 2004 (Note of Clarification on Paragraph 30 added)
**59th WMA General Assembly, Seoul, October 2008**

## A. INTRODUCTION

1. The World Medical Association (WMA) has developed the Declaration of Helsinki as a statement of ethical principles for medical research involving human subjects, including research on identifiable human material and data. The Declaration is intended to be read as a whole and each of its constituent paragraphs should not be applied without consideration of all other relevant paragraphs.

2. Although the Declaration is addressed primarily to physicians, the WMA encourages other participants in medical research involving human subjects to adopt these principles.

3. It is the duty of the physician to promote and safeguard the health of patients, including those who are involved in medical research. The physician's knowledge and conscience are dedicated to the fulfilment of this duty.

4. The Declaration of Geneva of the WMA binds the physician with the words, "The health of my patient will be my first consideration," and the International Code of Medical Ethics declares that, "A physician shall act in the patient's best interest when providing medical care."

5. Medical progress is based on research that ultimately must include studies involving human subjects. Populations that are underrepresented in medical research should be provided appropriate access to participation in research.

6. In medical research involving human subjects, the well-being of the individual research subject must take precedence over all other interests.

7. The primary purpose of medical research involving human subjects is to understand the causes, development and effects of diseases and improve preventive, diagnostic and therapeutic interventions (methods, procedures and treatments). Even the best current interventions must be evaluated continually through research for their safety, effectiveness, efficiency, accessibility and quality.

8. In medical practice and in medical research, most interventions involve risks and burdens.

9. Medical research is subject to ethical standards that promote respect for all human subjects and protect their health and rights. Some research populations are particularly vulnerable and need special protection. These include those who cannot give or refuse consent for themselves and those who may be vulnerable to coercion or undue influence.

10. Physicians should consider the ethical, legal and regulatory norms and standards for research involving human subjects in their own countries as well as applicable international norms and standards. No national or international ethical, legal or regulatory requirement should reduce or eliminate any of the protections for research subjects set forth in this Declaration.

## B. PRINCIPLES FOR ALL MEDICAL RESEARCH

11. It is the duty of physicians who participate in medical research to protect the life, health, dignity, integrity, right to self-determination, privacy, and confidentiality of personal information of research subjects.

12. Medical research involving human subjects must conform to generally accepted scientific principles, be based on a thorough knowledge of the scientific literature, other relevant sources of information, and adequate laboratory and, as appropriate, animal experimentation. The welfare of animals used for research must be respected.

13. Appropriate caution must be exercised in the conduct of medical research that may harm the environment.

14. The design and performance of each research study involving human subjects must be clearly described in a research protocol. The protocol should contain a statement of the ethical considerations involved and should indicate how the principles in this Declaration have been addressed. The protocol should include information regarding funding, sponsors, institutional affiliations, other potential conflicts of interest, incentives for subjects and provisions for treating and/or compensating subjects who are harmed as a consequence of participation in the research study. The protocol should describe arrangements for post-study access by study subjects to interventions identified as beneficial in the study or access to other appropriate care or benefits.

15. The research protocol must be submitted for consideration, comment, guidance and approval to a research ethics committee before the study begins. This committee must be independent of the researcher, the sponsor and any other undue influence. It must take into consideration the laws and regulations of the country or countries in which the research is to be performed as well as applicable international norms and standards but these must not be allowed to reduce or eliminate any of the protections for research subjects set forth in this Declaration. The committee must have the right to monitor ongoing studies. The researcher must provide monitoring information to the committee, especially information about any serious adverse events. No change to the protocol may be made without consideration and approval by the committee.

16. Medical research involving human subjects must be conducted only by individuals with the appropriate scientific training and qualifications. Research on patients or healthy volunteers requires the supervision of a competent and appropriately qualified physician or other health care professional. The responsibility for the protection of research subjects must always rest with the physician or other health care professional and never the research subjects, even though they have given consent.

17. Medical research involving a disadvantaged or vulnerable population or community is only justified if the research is responsive to the health needs and priorities of this population or community and if there is a reasonable likelihood that this population or community stands to benefit from the results of the research.

18. Every medical research study involving human subjects must be preceded by careful assessment of predictable risks and burdens to the individuals and communities involved in the

research in comparison with foreseeable benefits to them and to other individuals or communities affected by the condition under investigation.

19. Every clinical trial must be registered in a publicly accessible database before recruitment of the first subject.

20. Physicians may not participate in a research study involving human subjects unless they are confident that the risks involved have been adequately assessed and can be satisfactorily managed. Physicians must immediately stop a study when the risks are found to outweigh the potential benefits or when there is conclusive proof of positive and beneficial results.

21. Medical research involving human subjects may only be conducted if the importance of the objective outweighs the inherent risks and burdens to the research subjects.

22. Participation by competent individuals as subjects in medical research must be voluntary. Although it may be appropriate to consult family members or community leaders, no competent individual may be enrolled in a research study unless he or she freely agrees.

23. Every precaution must be taken to protect the privacy of research subjects and the confidentiality of their personal information and to minimize the impact of the study on their physical, mental and social integrity.

24. In medical research involving competent human subjects, each potential subject must be adequately informed of the aims, methods, sources of funding, any possible conflicts of interest, institutional affiliations of the researcher, the anticipated benefits and potential risks of the study and the discomfort it may entail, and any other relevant aspects of the study. The potential subject must be informed of the right to refuse to participate in the study or to withdraw consent to participate at any time without reprisal. Special attention should be given to the specific information needs of individual potential subjects as well as to the methods used to deliver the information. After ensuring that the potential subject has understood the information, the physician or another appropriately qualified individual must then seek the potential subject's freely-given informed consent, preferably in writing. If the consent cannot be expressed in writing, the non-written consent must be formally documented and witnessed.

25. For medical research using identifiable human material or data, physicians must normally seek consent for the collection, analysis, storage and/or reuse. There may be situations where consent would be impossible or impractical to obtain for such research or would pose a threat to the validity of the research. In such situations the research may be done only after consideration and approval of a research ethics committee.

26. When seeking informed consent for participation in a research study the physician should be particularly cautious if the potential subject is in a dependent relationship with the physician or may consent under duress. In such situations the informed consent should be sought by an appropriately qualified individual who is completely independent of this relationship.

27. For a potential research subject who is incompetent, the physician must seek informed consent from the legally authorized representative. These individuals must not be included in a research study that has no likelihood of benefit for them unless it is intended to promote the health of the population represented by the potential subject, the research cannot instead be performed with competent persons, and the research entails only minimal risk and minimal burden.

28. When a potential research subject who is deemed incompetent is able to give assent to decisions about participation in research, the physician must seek that assent in addition to the

consent of the legally authorized representative. The potential subject's dissent should be respected.

29. Research involving subjects who are physically or mentally incapable of giving consent, for example, unconscious patients, may be done only if the physical or mental condition that prevents giving informed consent is a necessary characteristic of the research population. In such circumstances the physician should seek informed consent from the legally authorized representative. If no such representative is available and if the research cannot be delayed, the study may proceed without informed consent provided that the specific reasons for involving subjects with a condition that renders them unable to give informed consent have been stated in the research protocol and the study has been approved by a research ethics committee. Consent to remain in the research should be obtained as soon as possible from the subject or a legally authorized representative.

30. Authors, editors and publishers all have ethical obligations with regard to the publication of the results of research. Authors have a duty to make publicly available the results of their research on human subjects and are accountable for the completeness and accuracy of their reports. They should adhere to accepted guidelines for ethical reporting. Negative and inconclusive as well as positive results should be published or otherwise made publicly available. Sources of funding, institutional affiliations and conflicts of interest should be declared in the publication. Reports of research not in accordance with the principles of this Declaration should not be accepted for publication.

## C. ADDITIONAL PRINCIPLES FOR MEDICAL RESEARCH COMBINED WITH MEDICAL CARE

31. The physician may combine medical research with medical care only to the extent that the research is justified by its potential preventive, diagnostic or therapeutic value and if the physician has good reason to believe that participation in the research study will not adversely affect the health of the patients who serve as research subjects.

32. The benefits, risks, burdens and effectiveness of a new intervention must be tested against those of the best current proven intervention, except in the following circumstances:

- The use of placebo, or no treatment, is acceptable in studies where no current proven intervention exists; or
- Where for compelling and scientifically sound methodological reasons the use of placebo is necessary to determine the efficacy or safety of an intervention and the patients who receive placebo or no treatment will not be subject to any risk of serious or irreversible harm. Extreme care must be taken to avoid abuse of this option.

33. At the conclusion of the study, patients entered into the study are entitled to be informed about the outcome of the study and to share any benefits that result from it, for example, access to interventions identified as beneficial in the study or to other appropriate care or benefits.

34. The physician must fully inform the patient which aspects of the care are related to the research. The refusal of a patient to participate in a study or the patient's decision to withdraw from the study must never interfere with the patient-physician relationship.

35. In the treatment of a patient, where proven interventions do not exist or have been ineffective, the physician, after seeking expert advice, with informed consent from the patient or a legally authorized representative, may use an unproven intervention if in the physician's judgement it offers hope of saving life, re-establishing health or alleviating suffering. Where possible, this intervention should be made the object of research, designed to evaluate its safety and efficacy. In all cases, new information should be recorded and, where appropriate, made publicly available.

# Appendix 12. Trials Flow

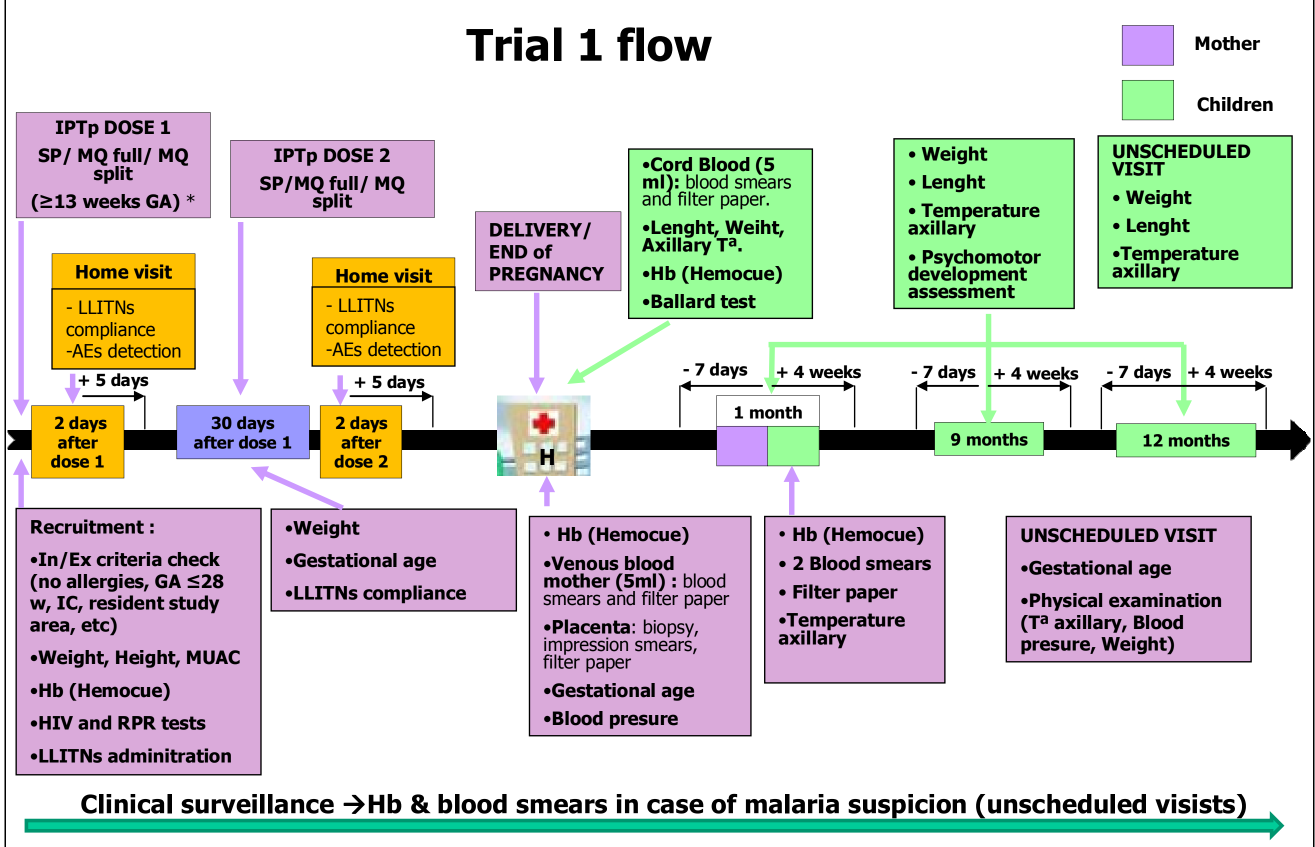


* Recruitment and IPTp dose 1 administration is taken place the same day only if gestational age is > 13 weeks GA, if it´s <13 weeks invite to adminitration when GA is reached.
.Blood smear and Hb: in all women passively reporting sick AND presenting with malaria related sintomatology or in children if Tª is > 37´5.
•Assessment of compliance with LLITNs use and Record of concomitant medication/Adverse events at all visits schedules visits

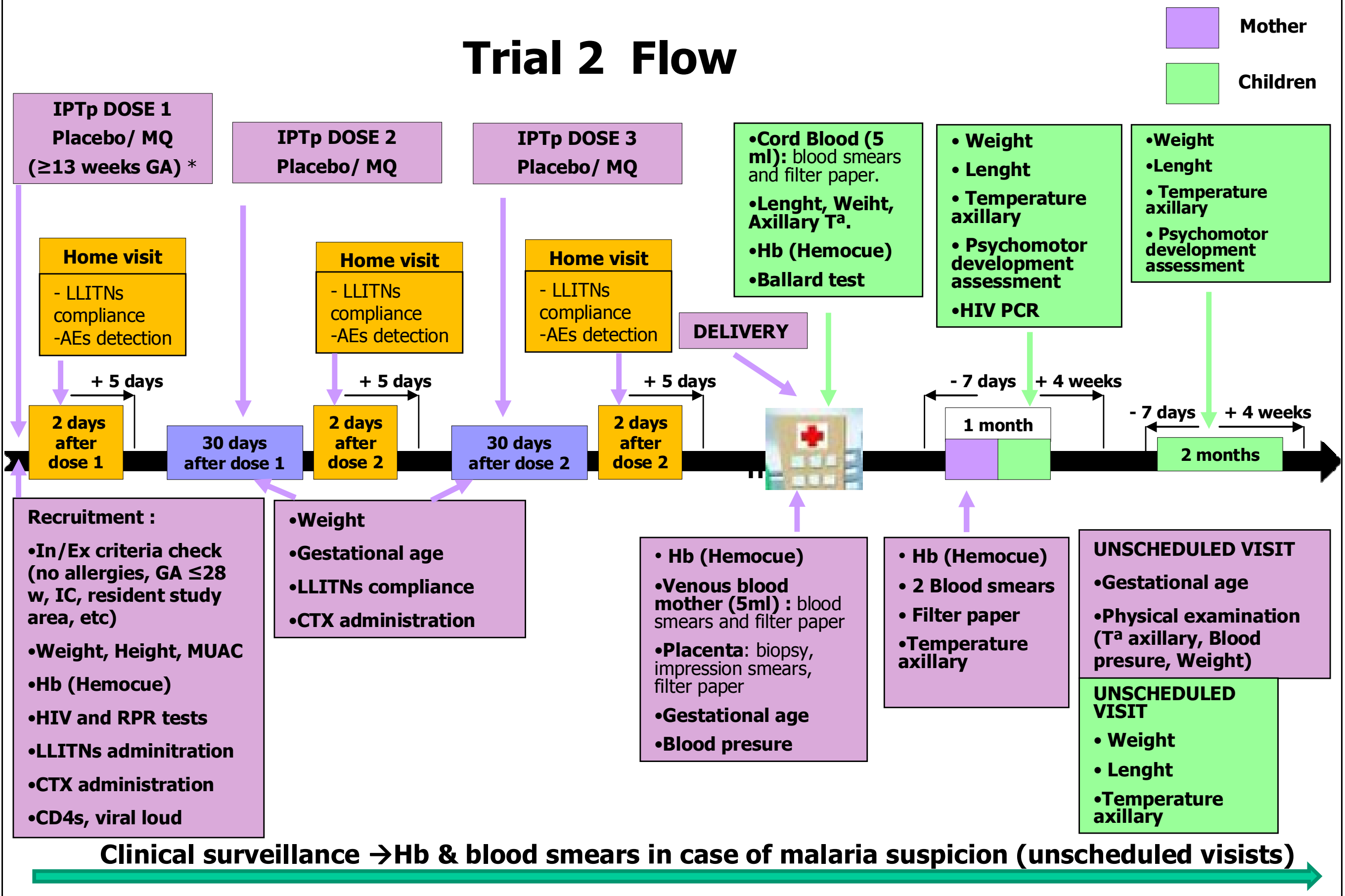


* Recruitment and IPTp dose 1 administration is taken place the same day only if gestational age is > 13 weeks GA, if it´s <13 weeks invite to adminitration when GA is reached.
.Blood smear and Hb: in all women passively reporting sick AND presenting with malaria related sintomatology or in children if Tª is ≥ 37´5.
•Assessment of compliance with LLITNs use and Record of concomitant medication/Adverse events at all visits schedules visits
