# Supplementary material for: Intermittent Preventive Treatment of Malaria in Pregnancy with Mefloquine in HIV-Negative Women: A Multicentre Randomized Controlled Trial
Source: PLoS Med. 2014 Sep 23;11(9):e1001733. doi: 10.1371/journal.pmed.1001733 (PMC4172436; doi:10.1371/journal.pmed.1001733)
Supplement: Text S4 — ClinicalTrials.gov registration receipt. (PDF) [file pmed.1001733.s013.pdf]

Protocol Registration Receipt
02/04/2009

Evaluation of Alternative Antimalarial Drugs for Malaria in Pregnancy (MiPPAD)

This study is not yet open for participant recruitment.
Verified by Hospital Clinic of Barcelona, January 2009

Sponsored by:	Hospital Clinic of Barcelona<br>Centre de Recerca en Salut Internacional de Barcelona (CRESIB). Spain<br>Institute of Tropical Medicine, University of Tübingen, Germany<br>Institut de Recherche pour le Développement (IRD), France<br>Faculté des Sciences de la Santé (FSS), Université d'Abomey Calavi, Cotonou, Benin<br>Medical Research Unit (MRU), Albert Schweitzer Hospital. Lambaréné, Gabon<br>Kenya Medical Research Institute<br>Ifakara Health Institute (IHI). Ifakara, Tanzania<br>Centro de Investigaçao em Saúde da Manhiça (CISM). Manhiça, Mozambique.<br>Vienna School of Clinical Research (VSCR), Austria.<br>US Centers for Disease Control and Prevention<br>Malaria in Pregnancy (MiP) Consortium
Information provided by:	Hospital Clinic of Barcelona
ClinicalTrials.gov Identifier:	NCT00811421

Purpose

The study aims at comparing the safety, tolerability and efficacy of Mefloquine (MQ) to Sulfadoxine-Pyrimethamine (SP) as Interment Preventive Treatment in pregnancy (IPTp) for the prevention of malaria effects on the mother and her infant.

Condition	Intervention	Phase
Pregnancy<br>Malaria Prevention<br>HIV Infections	Drug: Sulphadoxine-pyrimethamine<br>Drug: Mefloquine<br>Drug: Placebo	Phase 4

Study Type: Interventional

Study Design: Prevention, Parallel Assignment, Double Blind (Subject, Investigator), Randomized, Safety/Efficacy Study

Official Title: Evaluation of the Safety and Efficacy of Mefloquine as Intermittent Preventive Treatment of Malaria in Pregnancy

Further study details as provided by Hospital Clinic of Barcelona:

Primary Outcome Measure:

- Trial 1 (IPTp MQ vs IPTp SP): Low birth weight.   [Time Frame: 0 day birth] [Designated as safety issue: No]
- Trial 2 (CTX+IPTp MQ vs CTX+IPTp placebo): Peripheral parasitaemia   [Time Frame: 0 day delivery] [Designated as safety issue: No]

Secondary Outcome Measures:

- Trial 1:Prevalence of placental P. falciparum infection.Prevalence of moderate maternal anaemia at delivery.   [Time Frame: 0 day delivery] [Designated as safety issue: No]
- Trial 2:Prevalence of placental P. falciparum infection.Prevalence of low birth weight babies (<2500 g)   [Time Frame: 0 day birth] [Designated as safety issue: No]

Estimated Enrollment: 5330

Arms	Assigned Interventions
Active Comparator: Trial 1: IPTp-SP+LLITNs<br>HIV-negative pregnant women receiving 2 doses of IPTp (500mg of sulfadoxine and 25 mg of pyrimethamine) in the context of long lasting Insecticide Treated Nets (LLITNs)	Drug: Sulphadoxine-pyrimethamine<br>SP oral administration (500mg sulphadoxine and 25mg pyrimethamine) as IPTp at the 1st and 2nd Antenatal Clinic visit
Experimental: Trial 1: IPTp-MQ+ LLITNs<br>HIV-negative pregnant women receiving 2 doses of IPTp (15 mg/Kg) in the context of long lasting Insecticide Treated Nets (LLITNs)	Drug: Mefloquine<br>MQ oral administration (15 mg/Kg) at the 1st and 2nd Antenatal Clinic visit as IPTp
Placebo Comparator: Trial 2: CTX+IPTp-Placebo+LLITNs<br>HIV-positive pregnant women receiving 3	Drug: Placebo<br>MQ-placebo oral administration at the 1st, 2nd and 3rd Antenatal Clinic visit as IPTp

Arms	Assigned Interventions
doses of IPTp (placebo) in the context of long lasting Insecticide Treated Nets (LLITNs)	
Experimental: Trial 2: CTX + IPTp-MQ+ LLITNs<br>HIV-positive pregnant women receiving 3 doses of IPTp (15 mg/Kg) in the context of long lasting Insecticide Treated Nets (LLITNs)	Drug: Mefloquine<br>MQ oral administration (15 mg/Kg) at the 1st and 2nd Antenatal Clinic visit as IPTp

The current recommendation by the World Health Organization (WHO) to prevent malaria infection in pregnancy in areas of stable malaria transmission relies on:

- Prompt and effective case management of malaria illness
- The use of intermittent preventive treatment (IPTp) with at least 2 treatment doses of sulfadoxine-pyrimethamine (SP) and
- The use of insecticide treated nets (ITNs).

However, the spread of parasite resistance to SP, particularly in eastern Africa, and the significant overlap in some regions of malaria transmission and high prevalence of HIV infection, have raised concerns about the medium and long-term use of SP for IPTp.

HIV infection increases susceptibility to malaria and may reduce the efficacy of interventions. The evaluation of alternative antimalarials for IPTp is thus urgently needed also involving HIV infected women.

Of all the current available alternative antimalarial drugs, mefloquine (MQ) is the one that offers the most comparative advantages to SP.

The protocol includes two trials:

Trial 1. Randomized open-label trial comparing the safety and efficacy of SP versus MQ as IPTp in the context of ITNs (n= 4260). Five African countries participate in this trial: Benin, Gabon, Kenya, Mozambique and Tanzania.

Trial 2. Randomized double-blinded trial comparing MQ-IPTp to placebo IPTp in HIV pregnant women receiving Cotrimoxazole prophylaxis and in the context of ITNs (n= 1070). Three African counties participate in this trial: Kenya, Mozambique and Tanzania.

Eligibility

Genders Eligible for Study: Female
Accepts healthy volunteers.

Inclusion Criteria:

Trial 1:

- Permanent resident in the area
- Gestational age at the first antenatal visit ≤ 28 weeks
- Signed informed consent
- Agreement to deliver in the study site's maternity(ies) wards

Trial 2:

- Permanent resident in the area.
- Gestational age at the first antenatal visit ≤ 28 weeks
- HIV seropositive (after voluntary counseling and testing)
- Indication to receive CTX prophylaxis (according to the national guidelines)
- Signed informed consent
- Agreement to deliver in the study site's maternity(ies) wards.

Exclusion Criteria:

Trial 1:

- Residence outside the study area or planning to move out in the following 18 months from enrollment
- Gestational age at the first antenatal visit > 28 weeks of pregnancy
- Known history of allergy to sulfa drugs or mefloquine
- Known history of severe renal, hepatic, psychiatric or neurological disease
- MQ or halofantrine treatment in the preceding 4 weeks
- HIV infection
- Participating in other studies

Trial 2:

- Residence outside the study area or planning to move out in the following 10 months from enrollment
- Gestational age at the first antenatal visit > 28 weeks of pregnancy
- Known history of allergy to CTX or MQ
- Known history of severe renal, hepatic, psychiatric or neurological disease
- MQ or halofantrine treatment in the preceding 4 weeks

Contacts and Locations

Contacts

Clara Menendez, MD, PhD	34 93 2275400	Menendez@clinic.ub.es
Raquel González, MD, MPH	34 93 2275400	ragonza@clinic.ub.es

Locations

Spain, Barcelona

Centre Recerca en Salut Internacional de Barcelona (CRESIB)

Barcelona, Barcelona, Spain, 08036

Contact: Clara Menendez, MD, PhD 34 93 2275400 menendez@clinic.ub.es

Investigators

Principal Investigator:	Clara Menendez, MD, PhD	CRESIB

More Information

Responsible Party:	Fundacio Clinic per la Recerca Biomedica (FCRB) Spain (Professor Clara Menendez Santos)
Study ID Numbers:	IP.07.31080.002
Health Authority:	Spain: Ethics Committee; Benin: Ethics Committee; Gabon:Ethics Committee; United States: Institutional Review Board; Kenya: Ethical Review Committee; Tanzania:Ethics Committee; Mozambique: Ethics Committee
